# Evaluation of Simpler Criteria for Diagnosing Allergic Bronchopulmonary Aspergillosis Complicating Asthma

**DOI:** 10.3389/fcimb.2022.861866

**Published:** 2022-03-25

**Authors:** Ritesh Agarwal, Puneet Saxena, Valliappan Muthu, Inderpaul Singh Sehgal, Sahajal Dhooria, Kuruswamy Thurai Prasad, Ashutosh Nath Aggarwal, Arunaloke Chakrabarti

**Affiliations:** ^1^Department of Pulmonary Medicine, Postgraduate Institute of Medical Education and Research (PGIMER), Chandigarh, India; ^2^Department of Medical Microbiology, Postgraduate Institute of Medical Education and Research (PGIMER), Chandigarh, India

**Keywords:** allergic bronchopulmonary mycosis (ABPM), *Aspergillus*, fungal sensitization, allergy, latent class analysis (LCA), bronchial asthma, cystic fibrosis

## Abstract

**Background:**

The modified International Society for Human and Animal Mycology (ISHAM) allergic bronchopulmonary aspergillosis (ABPA) working group (AWG) criteria lists up to five components for diagnosing ABPA in asthmatics. Whether eliminating specific components of the existing criteria would have the same diagnostic utility as the original remains unknown.

**Objective:**

To evaluate the performance of several simplified criteria for diagnosing ABPA.

**Methods:**

We compared the performance of seven new criteria (after excluding or modifying one or more of the components of the ISHAM-AWG criteria) with the modified ISHAM-AWG criteria in asthmatic subjects using latent class analysis (LCA). We also tested the performance of the newer criteria using accuracy measures against a multidisciplinary team (MDT) diagnosis of ABPA. We considered the diagnostic accuracy of the newer criteria to be acceptable if the correct classification and false-negative rates were >95% and <5%, respectively, on an MDT evaluation.

**Results:**

We analyzed data from 543 asthmatic subjects (58.8% women; mean age, 36.8 years). Using LCA, the sensitivity of the *A.fumigatus*-specific IgE-based criteria ranged from 92-99%, while the specificity varied between 92% and 100%. The MDT diagnosed ABPA in 106 (19.5%) subjects. Using MDT as the reference standard, the correct classification and false-negative rates were >95% for three of the seven and <5% for four of the seven newer criteria.

**Conclusions:**

We found several of the newly developed criteria to perform, like the modified ISHAM-AWG criteria, for diagnosing ABPA complicating asthma. A prospective study in current clinical algorithms is required for validating our observations.

## Introduction

Allergic bronchopulmonary aspergillosis (ABPA) is a pulmonary disorder caused by complex hypersensitivity reactions targeted against *Aspergillus fumigatus* colonizing the airways of patients with asthma (and cystic fibrosis) (Agarwal et al., 2022). ABPA patients typically present with difficult-to-control asthma, recurrent pulmonary opacities, and bronchiectasis (Kosmidis and Denning, 2015). Several criteria based on a composite of imaging and immunological parameters are available for diagnosing ABPA (Rosenberg et al., 1977; Agarwal et al., 2010; Agarwal et al., 2013; Agarwal et al., 2013; Dhooria and Agarwal, 2014; Asano et al., 2021; Saxena et al., 2021). Rosenberg et al. were the first to suggest a criterion for diagnosing ABPA (Rosenberg et al., 1977). Subsequently, several modifications to these criteria have been proposed to incorporate the advances in immunological tests.

In 2013, the ABPA working group (AWG) of the International Society for Human and Animal Mycology (ISHAM) had framed a criterion for diagnosing ABPA in asthmatics (Agarwal et al., 2013). The ISHAM-AWG criterion relies on certain thresholds of a combination of several immunological and other tests. Although this criterion is a refinement over previously available diagnostic algorithms, it would be interesting to both clinicians and patients if the diagnostic standards could be made even easier. Recently, we proposed specific changes to the ISHAM-AWG criteria that made the criteria simpler and resulted in better sensitivity and specificity (Saxena et al., 2021). The current study aimed to evaluate the performance of several simpler criteria for diagnosing ABPA.

## Methods

We conducted a retrospective analysis of prospectively collected data (July 2017 and September 2018) at our institute’s chest clinic. The Institute Ethics Committee approved the study protocol. We obtained written informed consent from all the participants.

### Patients

We have published the detailed methodology and the patient details previously (Saxena et al., 2021). Briefly, we included consecutive cases of physician-diagnosed bronchial asthma. We classified the asthma severity based on the 2004 Global Initiative for Asthma (GINA) recommendations that adjusts for the effect of treatment on disease severity (Global Initiative for Asthma (GINA), 2004).

### Study Procedure

We performed spirometry, *Aspergillus* skin test (AST), *A.fumigatus*-specific IgE and IgG, serum total IgE, peripheral blood total eosinophil count (TEC), chest radiograph, and computed tomography (CT) of the chest in all the asthmatic subjects. We performed AST intradermally using the crude *A.fumigatus* antigen (Alcit India, New Delhi, India), and an immediate cutaneous hyperreactivity was considered a positive AST (Agarwal et al., 2013). Serum total IgE, *A.fumigatus*-specific IgE, and IgG were assayed using the commercially available fluorescent enzyme immunoassay (Phadia 100, Thermofisher Scientific, Uppsala, Sweden) (Agarwal et al., 2016; Agarwal et al., 2017). We classified the chest radiographic findings as transient or permanent (Agarwal et al., 2016). We obtained CT of the thorax using a multidetector CT scanner and defined bronchiectasis according to previously described criteria (Agarwal et al., 2011). We performed spirometry on an ultrasonic flow-sensing spirometer and interpreted the spirometric values, namely the forced expiratory volume in the first second (FEV1) and the forced vital capacity (FVC), using the Indian guidelines (Aggarwal et al., 2019).

#### Newer, Simpler Criteria

The modified ISHAM-AWG criterion requires up to 5 components for diagnosing ABPA in asthmatics (**Table 1**). We tested five criteria (criteria 1, 2, 5, 6, and 7) with only four components (eliminating TEC or *A.fumigatus*-specific IgG or CT) and two criteria (criteria 3 and 4) with only three components (eliminating both TEC and *A.fumigatus*-specific IgG). All the criteria included *A.fumigatus*-specific IgE except criteria 4 that replaced it with AST. Criteria 1 and 2 were like criteria 5 and 6, respectively, except for a higher threshold for *A.fumigatus*-specific IgE (0.5 kUA/L instead of 0.35 kUA/L). Criteria 4 was similar to criteria 3, except it replaced *A.fumigatus*-specific IgE with AST. Finally, criteria 7 was solely an immunological criterion, and we excluded CT chest.

**Table 1:** Various proposed simpler criteria for allergic bronchopulmonary aspergillosis (ABPA) evaluated in the current study.

Modified ISHAM-AWG criteria
Presence of both: (1) serum *Af*-specific IgE >0.35 kUA/L, and (2) serum total IgE >500 IU/mL AND two of the following: (1) serum *Af*-specific IgG >27 mgA/L; (2) bronchiectasis on CT chest; (3) TEC >500 cells/µL
**New criteria**
Criteria 1: Both: (1) serum *Af*-specific IgE >0.5 kUA/L, and (2) serum total IgE >500 IU/mL AND any of the following: (1) serum *Af*-specific IgG >27 mgA/L; (2) bronchiectasis on CT chest
Criteria 2: Both: (1) serum *Af*-specific IgE >0.5 kUA/L, and (2) serum total IgE >500 IU/mL AND any of the following: (1) bronchiectasis on CT chest; (2) TEC >500 cells/µL
Criteria 3: All the following: (1) serum *Af*-specific IgE >0.35 kUA/L; (2) serum total IgE >500 IU/mL; (3) bronchiectasis on CT chest
Criteria 4: All the following: (1) type 1 Aspergillus skin test positive; (2) serum total IgE >500 IU/mL; (3) bronchiectasis on CT chest
Criteria 5: Both: (1) serum *Af*-specific IgE >0.35 kUA/L, and (2) serum total IgE >500 IU/mL AND any of the following: (1) serum *Af*-specific IgG >27 mgA/L; (2) bronchiectasis on CT chest
Criteria 6: Both: (1) serum *Af*-specific IgE >0.35 kUA/L, and (2) serum total IgE >500 IU/mL AND any of the following: (1) bronchiectasis on CT chest; (2) TEC >500 cells/µL
Criteria 7: Both: (1) serum *Af*-specific IgE >0.35 kUA/L, and (2) serum total IgE >500 IU/mL AND any of the following: (1) serum *Af*-specific IgG >27 mgA/L; (2) TEC >500 cells/µL

Af, Aspergillus fumigatus; AWG, ABPA working group; CT, computed tomography; ISHAM, international society for human and animal mycology; TEC, peripheral blood total eosinophil count.

### Objectives

The study objectives were to (1) compare the diagnostic performance of seven simpler standards (**Table 1**) with the recently published modified ISHAM-AWG criteria for diagnosing ABPA, using latent class analysis (LCA); (2) evaluate the diagnostic performance of the different newer benchmarks for detecting ABPA against a multidisciplinary team (MDT) diagnosis of ABPA.

### Statistical Analysis

We analyzed data using the commercial statistical package SPSS version 22.0 (IBM SPSS Inc., Armonk NY, US) and present the data as number (percentage) or mean (standard deviation [SD]). We performed LCA using the R version of the TAGS software (Pouillot et al., 2002; Agarwal et al., 2013; Saxena et al., 2021). For performing LCA, we divided the asthma population into mild-to-moderate (consisting of mild intermittent, mild persistent, and moderate persistent asthma) or severe (comprising of severe persistent asthma), according to the 2004 GINA guidelines (Global Initiative for Asthma (GINA), 2004). We also analyzed the difference in LCA results when the study population is not divided into two groups (single group). We calculated the sensitivity and specificity (with corresponding 2.5-97.5% bootstrap confidence intervals [CI]), obtained by bootstrapping 5000 samples) using the maximum likelihood (ML) estimates. We used the Newton-Raphson and the expectation-maximization algorithms for deriving the ML estimates (Pouillot et al., 2002). We assumed conditional independence using the goodness-of-fit test and subsequently deduced the residual correlations between tests. A p-value >0.05 and the random distribution of the residuals near-zero indicated a good fit of the model. We used the DAG_stat application for calculating the sensitivity, specificity, correct classification rate (efficiency), and false-negative rate with 95% CI of the various criteria using MDT diagnosis as the reference standard (Mackinnon, 2000). We assumed the diagnostic accuracy of the newer criteria to be acceptable if the efficiency was >95% and the false-negative rate <5%.

## Results

We included 543 asthmatic subjects (58.8% women) with a mean (SD) age of 36.8 (13.9) years. There were 338 and 205 subjects with mild-to-moderate and severe asthma, respectively. The proportion of subjects with aspergillus skin test positivity, serum total IgE level >500 IU/mL, serum *A.fumigatus*-specific IgE values >0.35 and >0.5 kUA/L, serum *A.fumigatus*-specific IgG >27 mgA/L, and peripheral blood TEC >500 cells/µL were similar between the two groups (**Table 2**). We encountered bronchiectasis in 24.3% of the study subjects.

**Table 2:** Select baseline characteristics of the study population.

	Total (n=543)	Mild-to-moderate asthma (n=338)	Severe asthma (n=205)	P value
Age (years)	36.8 ± 13.9	34.9 ± 13.5	39.9 ± 13.8	0.0001
Female sex	319 (58.8)	186 (55.0)	133 (64.9)	0.024
Body mass index (Kg/m^2^)	23.6 ± 5.2	23.5 ± 5.2	23.8 ± 5.2	0.55
Asthma duration (years)	10.2 ± 9.5	9.0 ± 8.8	12.0 ± 10.3	0.0001
**Spirometry**				
FEV1 (% predicted)	73.5 ± 25.6	77.7 ± 24.0	66.6 ± 26.7	0.0001
FVC (% predicted)	82.7 ± 20.7	85.9 ± 19.8	77.2 ± 21.0	0.0001
**Immunologic investigations**				
Type 1 *Af* skin test positive	164 (30.2)	103 (30.5)	61 (29.8)	0.86
Serum *Af*-specific IgE >0.35 kUA/L	219 (40.5)	133 (39.5)	86 (42.2)	0.54
Serum *Af*-specific IgE >0.5 kUA/L	201 (37.0)	123 (36.4)	78 (33.0)	0.70
Serum *Af*-specific IgG >27 mgA/L	194 (35.7)	119 (35.2)	75 (36.6)	0.75
Peripheral blood eosinophil count >500 cells/µL	223 (41.1)	132 (39.1)	91 (44.4)	0.22
Serum total IgE level >500 IU/mL	300 (55.3)	186 (55)	114 (55.6)	0.90
**Bronchiectasis on CT chest**	132 (24.3)	73 (21.6)	59 (28.8)	0.059

All values are represented as mean ± SD or number (percentage) unless otherwise indicated.

Af, Aspergillus fumigatus; CT, computed tomography; FEV1, forced expiratory volume in the first second; FVC, forced vital capacity.

Mild-to-moderate asthma consisted of mild intermittent, mild persistent, and moderate persistent, while severe asthma comprised of severe persistent category as per the 2004 GINA guidelines.

### Objective 1

We compared seven newer criteria with the modified ISHAM-AWG criteria using LCA (**Table 3**). The sensitivity of the *A.fumigatus*-specific IgE-based criteria (criteria 1, 2, 3, 5, 6, and 7) ranged from 92-99%, while the specificity varied between 92% and 100%. The diagnostic performance of *A.fumigatus*-specific IgE dependent criteria was almost similar to the modified ISHAM-AWG criteria. The sensitivity was lowest for the new criteria 4 [also known as minimal diagnostic criteria for ABPA (Schwartz and Greenberger, 1991)]; however, the specificity was nearly 100%. When the skin test in the minimal essential criteria was replaced with *A.fumigatus*-specific IgE (**Table 3**, criteria 3), the sensitivity of the minimal criteria increased to 92% with no loss of specificity. The specificity was lowest for the new criteria 7 that excluded imaging, although the sensitivity was 93.5%. The ML estimate for the model was -857.8 (deviance, 631.8). The p-value for the goodness-of-fit test for conditional independence was 0.00001, indicating that the model does not fit. We found the results similar even if the population is kept as a single group (**Table 3**).

**Table 3:** Diagnostic performance of various criteria for diagnosing allergic bronchopulmonary aspergillosis (ABPA) using latent class analysis.

	Two populations		Single population	
	Sensitivity (95% CI)	Specificity (95% CI)	Sensitivity (95% CI)	Specificity (95% CI)
**Modified ISHAM-AWG criteria**	94.4 (89.7-98.3)	100 (100-100)	94.4 (89.7-98.3)	100 (100-100)
**New criteria**				
Criteria 1	96.3 (92.4-99.2)	97.7 (96.2-99.1)	93.5 (88.6-97.9)	97.7 (96.2-99.1)
Criteria 2	98.1 (95.2-100)	95.4 (93.3-97.3)	98.1 (95.0-100)	95.4 (93.4-97.3)
Criteria 3	91.7 (86.0-96.6)	100 (100-100)	91.6 (86.0-96.4)	100 (100-100)
Criteria 4	73.1 (64.4-81.3)	99.8 (99.3-100)	73.1 (64.7-81.3)	99.8 (99.3-100)
Criteria 5	99.1 (96.9-100)	97.7 (96.2-99.1)	99.1 (96.7-100)	97.7 (96.2-99.1)
Criteria 6	99.1 (96.9-100)	94.0 (91.7-96.1)	99.1 (96.7-100)	94.0 (91.7-96.2)
Criteria 7	93.5 (88.6-97.9)	91.7 (88.9-94.2)	93.5 (88.5-97.9)	91.7 (89.1-94.3)

The values in parentheses are confidence intervals that correspond to 2.5-97.5% bootstrap confidence intervals (CI) obtained by bootstrapping 5000 samples.

ISHAM-AWG, international society for human and animal mycology ABPA working group criteria

The p-value for goodness-of-fit for the models is <0.05, indicating that the model does not fit.

The details of the various criteria are provided in **Table 1**.

### Objective 2

We further compared the diagnostic performance of various criteria using an MDT diagnosis as the reference standard. The MDT adjudicated 106 cases to have ABPA (**Table 4**). The correct classification rate was >95% for criteria 1, 3, and 5 (**Table 4**). The false-negative rate was <5% for all criteria except for criteria 3 (11.3%), 4 (26.5%), and 7 (26.3%).

**Table 4:** Performance of various diagnostic criteria for allergic bronchopulmonary aspergillosis (ABPA) using the multidisciplinary team (MDT) evaluation as the reference standard.

	MDT, Not ABPA (n=437)		MDT, ABPA (n=106)		Sensitivity (95% CI)	Specificity (95% CI)	Correct classification rate (95% CI)	False-negative rate (95% CI)
Criteria	Present	Absent	Present	Absent				
**Modified ISHAM-AWG criteria**	0	437	102	4	96.2 (90.6-98.9)	100 (99.2-100)	99.3 (98.1-99.8)	3.8 (1.0-9.4)
**New criteria**								
Criteria 1	14	423	102	4	96.2 (90.6-98.9)	96.8 (94.7-98.2)	96.7 (94.8-98.0)	3.8 (1.0-9.4)
Criteria 2	24	413	102	4	96.2 (90.6-98.9)	94.5 (91.9-96.5)	94.8 (92.6-96.6)	3.8 (1.0-9.4)
Criteria 3	05	432	94	12	88.7 (81.1-94.0)	98.9 (97.4-99.6)	96.9 (95.0-98.2)	11.3 (5.9-18.9)
Criteria 4	02	435	78	28	73.6 (64.1-81.7)	99.5 (98.4-99.9)	94.5 (92.2-96.2)	26.5 (18.3-35.9)
Criteria 5	15	422	102	4	96.2 (90.6-98.9)	96.6 (94.4-98.1)	96.5 (94.6-97.9)	3.8 (1.0-9.4)
Criteria 6	31	406	102	4	96.2 (90.6-98.9)	92.9 (90.1-95.1)	93.6 (91.2-95.5)	3.8 (1.0-9.4)
Criteria 7	36	401	101	5	73.7 (65.5-80.9)	98.8 (97.2-99.6)	92.5 (89.9-64.5)	26.3 (19.1-34.4)

CI, confidence intervals; ISHAM-AWG, international society for human and animal mycology ABPA working group criteria.

The details of the various criteria are provided in **Table 1**.

## Discussion

We found several simpler criteria to perform reasonably well than the modified ISHAM-AWG criteria. An optimal diagnostic criterion should be valid, reliable, and straightforward. Moreover, the criterion should have a clearly defined diagnostic threshold. The modified ISHAM-AWG meets most of these attributes but involves several tests, and less complex criteria would be welcome. While minimal criteria have been proposed in the past (Schwartz and Greenberger, 1991), none have been evaluated for their performance. We designed seven criteria and compared them with the modified ISHAM-AWG criteria. We found no benefit on LCA and MDT evaluation by increasing the *A.fumigatus*-specific IgE threshold from 0.35 to 0.5 kUA/L (criteria 1 and 2 vs. 5 and 6). The minimal essential diagnostic criteria for ABPA (Aspergillus skin test positivity, raised serum total IgE, and bronchiectasis; criteria 4) (Schwartz and Greenberger, 1991) had a sensitivity of only about 74%, thus potentially missing many cases despite having a nearly 100% specificity. There was a considerable increase in the sensitivity of the minimal essential criteria once skin test positivity was replaced by *A.fumigatus*-specific IgE (criteria 3). Finally, criteria 7 based on immunological components alone had a reasonable performance on LCA but lower on the MDT evaluation due to the higher number of patients with bronchiectasis in the study.

We have previously used latent class analysis (LCA), a relatively modern computational approach for refining the diagnostic criteria for ABPA (Agarwal et al., 2013; Saxena et al., 2021). The LCA statistically calculates the relationship between certain user-defined input variables (for example, the various diagnostic criteria) that estimate a latent variable, namely the presence or absence of disease (ABPA) (Rindskopf and Rindskopf, 1986; Goetghebeur et al., 2000). Importantly, the LCA technique provides diagnostic accuracy measures without requiring a 2 x 2 matrix that is usually required when a reference standard is used. Conventionally, for performing LCA, we divide the asthma population into two groups. This procedure is complicated as each criterion (n=8) has to be applied in every subject. Further, the presence or absence of the criteria is reported in a binary fashion (0 or 1), and we have 256 different permutations in two groups (**Table S1**). We evaluated whether the results would be different if the asthma population is not divided into two groups (**Table S2**). We found the results similar whether or not the population is divided into two groups (**Table 3**).

What is the clinical relevance of our study? We evaluated seven simpler models for diagnosing ABPA. The *A.fumigatus*-specific IgE-based criteria performed better than skin test-based criteria. Of the seven, the combination of IgE (total and *A.fumigatus*-specific) and either an elevated *A.fumigatus*-specific IgG or bronchiectasis (criteria 5) could be a reasonable alternative to the modified ISHAM-AWG criteria. Also, the specific IgE-based minimal essential criteria (criteria 3) might be another alternative, especially in resource-constrained settings, as it includes only three components. The skin test-based minimal essential criteria (criteria 4) may be used to confirm ABPA (specificity >99%) in settings without access to immunoassays. However, criteria 4 cannot be used to rule out ABPA, given its poor sensitivity (73%). Lastly, our study results provide clinicians with evidence regarding the certainty of diagnosing ABPA when one or more of the components of the existing criterion are missing.

Finally, our study has a few limitations. Our study is a single-center study conducted at a tertiary care hospital. Also, a large proportion of patients in our study had bronchiectasis. The diagnostic performance of the criteria might be different in the milder asthmatics seen in the community or those with serological ABPA. Another weakness can be attributed to the statistical technique as the probabilistic estimates cannot be perfect and depend on the positivity and negativity rates of the variables included in the model. Also, the goodness-of-fit for the model indicated that conditional independence was not met. Thus, the diagnostic performance may be fallible. While an MDT comparison of the various diagnostic criteria was also performed to circumvent this flaw, the criteria need to be validated prospectively. The study results are also not immediately applicable to other predisposing conditions for ABPA, including cystic fibrosis and others.

In conclusion, we found that specific IgE-based simpler criteria may be good alternatives for diagnosing ABPA. Because of the monocentric nature of our study, a prospective multicenter investigation is required for validating our study results.

## Data Availability Statement

The raw data supporting the conclusions of this article will be made available by the authors, without undue reservation.

## Ethics Statement

The studies involving human participants were reviewed and approved by Institute Ethics Committe, PGIMER, Chandigarh. The patients/participants provided their written informed consent to participate in this study.

## Author Contributions

RA conceived the idea, involved in patient management, statistical analysis, drafted, and revised the manuscript for intellectual content. PS: involved in patient management and data collection, revised the manuscript for intellectual content. VM: involved in patient management, statistical analysis, revised the manuscript. IS: involved in patient management, revised the manuscript. SD: involved in patient management, revised the manuscript. KP: involved in patient management, revised the manuscript. AA: involved in patient management, revised the manuscript for intellectual content. AC: involved in patient management, revised the manuscript for intellectual content. RA: guarantor of the paper. All authors contributed to the article and approved the submitted version.

## Conflict of Interest

RA has received grant support from Cipla, India on research in ABPA PS.

The remaining authors declare that the research was conducted in the absence of any commercial or financial relationships that could be construed as a potential conflict of interest.

## Publisher’s Note

All claims expressed in this article are solely those of the authors and do not necessarily represent those of their affiliated organizations, or those of the publisher, the editors and the reviewers. Any product that may be evaluated in this article, or claim that may be made by its manufacturer, is not guaranteed or endorsed by the publisher.
