# Supplementary material for: Evaluation of Simpler Criteria for Diagnosing Allergic Bronchopulmonary Aspergillosis Complicating Asthma
Source: Front Cell Infect Microbiol. 2022 Mar 25;12:861866. doi: 10.3389/fcimb.2022.861866 (PMC8990730; doi:10.3389/fcimb.2022.861866)
Supplement: Supplementary file 1 [file Table_1.pdf]

R version 4.1.2 (2021-11-01) -- "Bird Hippie"
Copyright (C) 2021 The R Foundation for Statistical Computing
Platform: x86_64-w64-mingw32/x64 (64-bit)

R is free software and comes with ABSOLUTELY NO WARRANTY.
You are welcome to redistribute it under certain conditions.
Type 'license()' or 'licence()' for distribution details.

Natural language support but running in an English locale

R is a collaborative project with many contributors.
Type 'contributors()' for more information and
'citation()' on how to cite R or R packages in publications.

Type 'demo()' for some demos, 'help()' for on-line help, or
'help.start()' for an HTML browser interface to help.
Type 'q()' to quit R.

[Previously saved workspace restored]

```
> > source("D:\\Dropbox\\Documents\\TAGS.R")
> TAGS()
```

TAGS V.2.0 is a R program developed by
R. Pouillot and G. Gerbier, Agence Française de Sécurité Sanitaire des Aliments, France.
r.pouillot@afssa.fr

Its purpose is to evaluate diagnostic tests in the absence of a gold standard,
using Maximum Likelihood Estimation (Newton Raphson and Expectation Maximisation algorithms).

For further details: see
Pouillot R., Gerbier G. (2001)
'Tags' a program for validation of the diagnostic values of tests in the absence of a gold standard.
Proceedings of the Society for Veterinary Epidemiology and Preventive Medecine, Noordwijkerhout, The netherlands, 28th - 30th March 2001: 37-48

Reference(s) population(s) data may be used (population(s) with a known infection status)

Evaluation may be used as soon as df>=parameters
A goodness-of-fit test and residual correlations are then provided

Three sets of data may be used as examples :
Hui and Walter (Biometrics, 1980, 36:167-171): Enter hui
Saegerman et al (Vet Record, 1999, 145:214-8): Enter sae
Handelman's dentistry data (JADA, 1986, 113:751-754): Enter qu
If you want to enter new data: Enter new
If you want to use loaded data: Enter the name the program has already given to you

data Set ? new
Enter a name for your data : new_8_double

ENTER YOUR data
Number of tests (between 1 and 10) ? 8
Number of population without unknown status (between 1 and 10) ? 2

Do you have Reference Population(s) ?
No : Enter 0
Yes, Disease free : Enter 1
Yes, Infected : Enter 2
Yes, one Disease-free and one Infected : Enter 3
0

population with unknown status 1
Number of results 0 0 0 0 0 0 0 0 in population with unknown status 1 : 254
Number of results 1 0 0 0 0 0 0 0 in population with unknown status 1 : 0
Number of results 0 1 0 0 0 0 0 0 in population with unknown status 1 : 0
Number of results 1 1 0 0 0 0 0 0 in population with unknown status 1 : 0
Number of results 0 0 1 0 0 0 0 0 in population with unknown status 1 : 0
Number of results 1 0 1 0 0 0 0 0 in population with unknown status 1 : 0
Number of results 0 1 1 0 0 0 0 0 in population with unknown status 1 : 0
Number of results 1 1 1 0 0 0 0 0 in population with unknown status 1 : 0
Number of results 0 0 0 1 0 0 0 0 in population with unknown status 1 : 0
Number of results 1 0 0 1 0 0 0 0 in population with unknown status 1 : 0
Number of results 0 1 0 1 0 0 0 0 in population with unknown status 1 : 0
Number of results 1 1 0 1 0 0 0 0 in population with unknown status 1 : 0
Number of results 0 0 1 1 0 0 0 0 in population with unknown status 1 : 0
Number of results 1 0 1 1 0 0 0 0 in population with unknown status 1 : 0
Number of results 0 1 1 1 0 0 0 0 in population with unknown status 1 : 0
Number of results 1 1 1 1 0 0 0 0 in population with unknown status 1 : 0
Number of results 0 0 0 0 1 0 0 0 in population with unknown status 1 : 0
Number of results 1 0 0 0 1 0 0 0 in population with unknown status 1 : 0
Number of results 0 1 0 0 1 0 0 0 in population with unknown status 1 : 0
Number of results 1 1 0 0 1 0 0 0 in population with unknown status 1 : 0
Number of results 0 0 1 0 1 0 0 0 in population with unknown status 1 : 0

Number of results 1 0 1 0 1 0 0 0 in population with unknown status 1 : 0
Number of results 0 1 1 0 1 0 0 0 in population with unknown status 1 : 0
Number of results 1 1 1 0 1 0 0 0 in population with unknown status 1 : 1
Number of results 0 0 0 1 1 0 0 0 in population with unknown status 1 : 0
Number of results 1 0 0 1 1 0 0 0 in population with unknown status 1 : 0
Number of results 0 1 0 1 1 0 0 0 in population with unknown status 1 : 0
Number of results 1 1 0 1 1 0 0 0 in population with unknown status 1 : 0
Number of results 0 0 1 1 1 0 0 0 in population with unknown status 1 : 0
Number of results 1 0 1 1 1 0 0 0 in population with unknown status 1 : 0
Number of results 0 1 1 1 1 0 0 0 in population with unknown status 1 : 0
Number of results 1 1 1 1 1 0 0 0 in population with unknown status 1 : 0
Number of results 0 0 0 0 0 1 0 0 in population with unknown status 1 : 0
Number of results 1 0 0 0 0 1 0 0 in population with unknown status 1 : 0
Number of results 0 1 0 0 0 1 0 0 in population with unknown status 1 : 0
Number of results 1 1 0 0 0 1 0 0 in population with unknown status 1 : 0
Number of results 0 0 1 0 0 1 0 0 in population with unknown status 1 : 0
Number of results 1 0 1 0 0 1 0 0 in population with unknown status 1 : 0
Number of results 0 1 1 0 0 1 0 0 in population with unknown status 1 : 0
Number of results 1 1 1 0 0 1 0 0 in population with unknown status 1 : 0
Number of results 0 0 0 1 0 1 0 0 in population with unknown status 1 : 0
Number of results 1 0 0 1 0 1 0 0 in population with unknown status 1 : 0
Number of results 0 1 0 1 0 1 0 0 in population with unknown status 1 : 0
Number of results 1 1 0 1 0 1 0 0 in population with unknown status 1 : 0
Number of results 0 0 1 1 0 1 0 0 in population with unknown status 1 : 0
Number of results 1 0 1 1 0 1 0 0 in population with unknown status 1 : 0
Number of results 0 1 1 1 0 1 0 0 in population with unknown status 1 : 0
Number of results 1 1 1 1 0 1 0 0 in population with unknown status 1 : 0
Number of results 0 0 0 0 1 1 0 0 in population with unknown status 1 : 0
Number of results 1 0 0 0 1 1 0 0 in population with unknown status 1 : 0
Number of results 0 1 0 0 1 1 0 0 in population with unknown status 1 : 0
Number of results 1 1 0 0 1 1 0 0 in population with unknown status 1 : 0
Number of results 0 0 1 0 1 1 0 0 in population with unknown status 1 : 0
Number of results 1 0 1 0 1 1 0 0 in population with unknown status 1 : 0
Number of results 0 1 1 0 1 1 0 0 in population with unknown status 1 : 0
Number of results 1 1 1 0 1 1 0 0 in population with unknown status 1 : 0
Number of results 0 0 0 1 1 1 0 0 in population with unknown status 1 : 0
Number of results 1 0 0 1 1 1 0 0 in population with unknown status 1 : 0
Number of results 0 1 0 1 1 1 0 0 in population with unknown status 1 : 0
Number of results 1 1 0 1 1 1 0 0 in population with unknown status 1 : 0
Number of results 0 0 1 1 1 1 0 0 in population with unknown status 1 : 0
Number of results 1 0 1 1 1 1 0 0 in population with unknown status 1 : 0
Number of results 0 1 1 1 1 1 0 0 in population with unknown status 1 : 0
Number of results 1 1 1 1 1 1 0 0 in population with unknown status 1 : 0
Number of results 0 0 0 0 0 0 1 0 in population with unknown status 1 : 0

Number of results 1 0 0 0 0 0 1 0 in population with unknown status 1 : 0
Number of results 0 1 0 0 0 0 1 0 in population with unknown status 1 : 0
Number of results 1 1 0 0 0 0 1 0 in population with unknown status 1 : 0
Number of results 0 0 1 0 0 0 1 0 in population with unknown status 1 : 0
Number of results 1 0 1 0 0 0 1 0 in population with unknown status 1 : 0
Number of results 0 1 1 0 0 0 1 0 in population with unknown status 1 : 0
Number of results 1 1 1 0 0 0 1 0 in population with unknown status 1 : 0
Number of results 0 0 0 1 0 0 1 0 in population with unknown status 1 : 0
Number of results 1 0 0 1 0 0 1 0 in population with unknown status 1 : 0
Number of results 0 1 0 1 0 0 1 0 in population with unknown status 1 : 0
Number of results 1 1 0 1 0 0 1 0 in population with unknown status 1 : 0
Number of results 0 0 1 1 0 0 1 0 in population with unknown status 1 : 0
Number of results 1 0 1 1 0 0 1 0 in population with unknown status 1 : 0
Number of results 0 1 1 1 0 0 1 0 in population with unknown status 1 : 0
Number of results 1 1 1 1 0 0 1 0 in population with unknown status 1 : 0
Number of results 0 0 0 0 1 0 1 0 in population with unknown status 1 : 0
Number of results 1 0 0 0 1 0 1 0 in population with unknown status 1 : 0
Number of results 0 1 0 0 1 0 1 0 in population with unknown status 1 : 0
Number of results 1 1 0 0 1 0 1 0 in population with unknown status 1 : 0
Number of results 0 0 1 0 1 0 1 0 in population with unknown status 1 : 0
Number of results 1 0 1 0 1 0 1 0 in population with unknown status 1 : 0
Number of results 0 1 1 0 1 0 1 0 in population with unknown status 1 : 0
Number of results 1 1 1 0 1 0 1 0 in population with unknown status 1 : 0
Number of results 0 0 0 1 1 0 1 0 in population with unknown status 1 : 0
Number of results 1 0 0 1 1 0 1 0 in population with unknown status 1 : 0
Number of results 0 1 0 1 1 0 1 0 in population with unknown status 1 : 0
Number of results 1 1 0 1 1 0 1 0 in population with unknown status 1 : 0
Number of results 0 0 1 1 1 0 1 0 in population with unknown status 1 : 0
Number of results 1 0 1 1 1 0 1 0 in population with unknown status 1 : 0
Number of results 0 1 1 1 1 0 1 0 in population with unknown status 1 : 0
Number of results 1 1 1 1 1 0 1 0 in population with unknown status 1 : 0
Number of results 0 0 0 0 0 1 1 0 in population with unknown status 1 : 0
Number of results 1 0 0 0 0 1 1 0 in population with unknown status 1 : 0
Number of results 0 1 0 0 0 1 1 0 in population with unknown status 1 : 0
Number of results 1 1 0 0 0 1 1 0 in population with unknown status 1 : 0
Number of results 0 0 1 0 0 1 1 0 in population with unknown status 1 : 0
Number of results 1 0 1 0 0 1 1 0 in population with unknown status 1 : 0
Number of results 0 1 1 0 0 1 1 0 in population with unknown status 1 : 0
Number of results 1 1 1 0 0 1 1 0 in population with unknown status 1 : 0
Number of results 0 0 0 1 0 1 1 0 in population with unknown status 1 : 0
Number of results 1 0 0 1 0 1 1 0 in population with unknown status 1 : 0
Number of results 0 1 0 1 0 1 1 0 in population with unknown status 1 : 0
Number of results 1 1 0 1 0 1 1 0 in population with unknown status 1 : 0
Number of results 0 0 1 1 0 1 1 0 in population with unknown status 1 : 0

Number of results 1 0 1 1 0 1 1 0 in population with unknown status 1 : 0
Number of results 0 1 1 1 0 1 1 0 in population with unknown status 1 : 1
Number of results 1 1 1 1 0 1 1 0 in population with unknown status 1 : 0
Number of results 0 0 0 0 1 1 1 0 in population with unknown status 1 : 0
Number of results 1 0 0 0 1 1 1 0 in population with unknown status 1 : 0
Number of results 0 1 0 0 1 1 1 0 in population with unknown status 1 : 0
Number of results 1 1 0 0 1 1 1 0 in population with unknown status 1 : 0
Number of results 0 0 1 0 1 1 1 0 in population with unknown status 1 : 0
Number of results 1 0 1 0 1 1 1 0 in population with unknown status 1 : 0
Number of results 0 1 1 0 1 1 1 0 in population with unknown status 1 : 0
Number of results 1 1 1 0 1 1 1 0 in population with unknown status 1 : 0
Number of results 0 0 0 1 1 1 1 0 in population with unknown status 1 : 1
Number of results 1 0 0 1 1 1 1 0 in population with unknown status 1 : 0
Number of results 0 1 0 1 1 1 1 0 in population with unknown status 1 : 0
Number of results 1 1 0 1 1 1 1 0 in population with unknown status 1 : 0
Number of results 0 0 1 1 1 1 1 0 in population with unknown status 1 : 0
Number of results 1 0 1 1 1 1 1 0 in population with unknown status 1 : 0
Number of results 0 1 1 1 1 1 1 0 in population with unknown status 1 : 2
Number of results 1 1 1 1 1 1 1 0 in population with unknown status 1 : 0
Number of results 0 0 0 0 0 0 0 1 in population with unknown status 1 : 0
Number of results 1 0 0 0 0 0 0 1 in population with unknown status 1 : 0
Number of results 0 1 0 0 0 0 0 1 in population with unknown status 1 : 0
Number of results 1 1 0 0 0 0 0 1 in population with unknown status 1 : 0
Number of results 0 0 1 0 0 0 0 1 in population with unknown status 1 : 0
Number of results 1 0 1 0 0 0 0 1 in population with unknown status 1 : 0
Number of results 0 1 1 0 0 0 0 1 in population with unknown status 1 : 0
Number of results 1 1 1 0 0 0 0 1 in population with unknown status 1 : 0
Number of results 0 0 0 1 0 0 0 1 in population with unknown status 1 : 0
Number of results 1 0 0 1 0 0 0 1 in population with unknown status 1 : 0
Number of results 0 1 0 1 0 0 0 1 in population with unknown status 1 : 0
Number of results 1 1 0 1 0 0 0 1 in population with unknown status 1 : 0
Number of results 0 0 1 1 0 0 0 1 in population with unknown status 1 : 0
Number of results 1 0 1 1 0 0 0 1 in population with unknown status 1 : 0
Number of results 0 1 1 1 0 0 0 1 in population with unknown status 1 : 0
Number of results 1 1 1 1 0 0 0 1 in population with unknown status 1 : 0
Number of results 0 0 0 0 1 0 0 1 in population with unknown status 1 : 0
Number of results 1 0 0 0 1 0 0 1 in population with unknown status 1 : 0
Number of results 0 1 0 0 1 0 0 1 in population with unknown status 1 : 0
Number of results 1 1 0 0 1 0 0 1 in population with unknown status 1 : 0
Number of results 0 0 1 0 1 0 0 1 in population with unknown status 1 : 0
Number of results 1 0 1 0 1 0 0 1 in population with unknown status 1 : 0
Number of results 0 1 1 0 1 0 0 1 in population with unknown status 1 : 0
Number of results 1 1 1 0 1 0 0 1 in population with unknown status 1 : 0
Number of results 0 0 0 1 1 0 0 1 in population with unknown status 1 : 0

Number of results 1 0 0 1 1 0 0 1 in population with unknown status 1 : 0
Number of results 0 1 0 1 1 0 0 1 in population with unknown status 1 : 0
Number of results 1 1 0 1 1 0 0 1 in population with unknown status 1 : 0
Number of results 0 0 1 1 1 0 0 1 in population with unknown status 1 : 0
Number of results 1 0 1 1 1 0 0 1 in population with unknown status 1 : 0
Number of results 0 1 1 1 1 0 0 1 in population with unknown status 1 : 0
Number of results 1 1 1 1 1 0 0 1 in population with unknown status 1 : 0
Number of results 0 0 0 0 0 1 0 1 in population with unknown status 1 : 0
Number of results 1 0 0 0 0 1 0 1 in population with unknown status 1 : 0
Number of results 0 1 0 0 0 1 0 1 in population with unknown status 1 : 6
Number of results 1 1 0 0 0 1 0 1 in population with unknown status 1 : 0
Number of results 0 0 1 0 0 1 0 1 in population with unknown status 1 : 0
Number of results 1 0 1 0 0 1 0 1 in population with unknown status 1 : 0
Number of results 0 1 1 0 0 1 0 1 in population with unknown status 1 : 0
Number of results 1 1 1 0 0 1 0 1 in population with unknown status 1 : 0
Number of results 0 0 0 1 0 1 0 1 in population with unknown status 1 : 0
Number of results 1 0 0 1 0 1 0 1 in population with unknown status 1 : 0
Number of results 0 1 0 1 0 1 0 1 in population with unknown status 1 : 0
Number of results 1 1 0 1 0 1 0 1 in population with unknown status 1 : 0
Number of results 0 0 1 1 0 1 0 1 in population with unknown status 1 : 0
Number of results 1 0 1 1 0 1 0 1 in population with unknown status 1 : 0
Number of results 0 1 1 1 0 1 0 1 in population with unknown status 1 : 0
Number of results 1 1 1 1 0 1 0 1 in population with unknown status 1 : 0
Number of results 0 0 0 0 1 1 0 1 in population with unknown status 1 : 0
Number of results 1 0 0 0 1 1 0 1 in population with unknown status 1 : 0
Number of results 0 1 0 0 1 1 0 1 in population with unknown status 1 : 0
Number of results 1 1 0 0 1 1 0 1 in population with unknown status 1 : 0
Number of results 0 0 1 0 1 1 0 1 in population with unknown status 1 : 0
Number of results 1 0 1 0 1 1 0 1 in population with unknown status 1 : 0
Number of results 0 1 1 0 1 1 0 1 in population with unknown status 1 : 0
Number of results 1 1 1 0 1 1 0 1 in population with unknown status 1 : 0
Number of results 0 0 0 1 1 1 0 1 in population with unknown status 1 : 0
Number of results 1 0 0 1 1 1 0 1 in population with unknown status 1 : 0
Number of results 0 1 0 1 1 1 0 1 in population with unknown status 1 : 0
Number of results 1 1 0 1 1 1 0 1 in population with unknown status 1 : 0
Number of results 0 0 1 1 1 1 0 1 in population with unknown status 1 : 0
Number of results 1 0 1 1 1 1 0 1 in population with unknown status 1 : 0
Number of results 0 1 1 1 1 1 0 1 in population with unknown status 1 : 0
Number of results 1 1 1 1 1 1 0 1 in population with unknown status 1 : 0
Number of results 0 0 0 0 0 0 1 1 in population with unknown status 1 : 4
Number of results 1 0 0 0 0 0 1 1 in population with unknown status 1 : 0
Number of results 0 1 0 0 0 0 1 1 in population with unknown status 1 : 0
Number of results 1 1 0 0 0 0 1 1 in population with unknown status 1 : 0
Number of results 0 0 1 0 0 0 1 1 in population with unknown status 1 : 11

Number of results 1 0 1 0 0 0 1 1 in population with unknown status 1 : 0
Number of results 0 1 1 0 0 0 1 1 in population with unknown status 1 : 0
Number of results 1 1 1 0 0 0 1 1 in population with unknown status 1 : 0
Number of results 0 0 0 1 0 0 1 1 in population with unknown status 1 : 0
Number of results 1 0 0 1 0 0 1 1 in population with unknown status 1 : 0
Number of results 0 1 0 1 0 0 1 1 in population with unknown status 1 : 0
Number of results 1 1 0 1 0 0 1 1 in population with unknown status 1 : 0
Number of results 0 0 1 1 0 0 1 1 in population with unknown status 1 : 0
Number of results 1 0 1 1 0 0 1 1 in population with unknown status 1 : 0
Number of results 0 1 1 1 0 0 1 1 in population with unknown status 1 : 0
Number of results 1 1 1 1 0 0 1 1 in population with unknown status 1 : 0
Number of results 0 0 0 0 1 0 1 1 in population with unknown status 1 : 0
Number of results 1 0 0 0 1 0 1 1 in population with unknown status 1 : 0
Number of results 0 1 0 0 1 0 1 1 in population with unknown status 1 : 0
Number of results 1 1 0 0 1 0 1 1 in population with unknown status 1 : 0
Number of results 0 0 1 0 1 0 1 1 in population with unknown status 1 : 0
Number of results 1 0 1 0 1 0 1 1 in population with unknown status 1 : 0
Number of results 0 1 1 0 1 0 1 1 in population with unknown status 1 : 0
Number of results 1 1 1 0 1 0 1 1 in population with unknown status 1 : 0
Number of results 0 0 0 1 1 0 1 1 in population with unknown status 1 : 0
Number of results 1 0 0 1 1 0 1 1 in population with unknown status 1 : 0
Number of results 0 1 0 1 1 0 1 1 in population with unknown status 1 : 0
Number of results 1 1 0 1 1 0 1 1 in population with unknown status 1 : 0
Number of results 0 0 1 1 1 0 1 1 in population with unknown status 1 : 0
Number of results 1 0 1 1 1 0 1 1 in population with unknown status 1 : 0
Number of results 0 1 1 1 1 0 1 1 in population with unknown status 1 : 0
Number of results 1 1 1 1 1 0 1 1 in population with unknown status 1 : 0
Number of results 0 0 0 0 0 1 1 1 in population with unknown status 1 : 0
Number of results 1 0 0 0 0 1 1 1 in population with unknown status 1 : 0
Number of results 0 1 0 0 0 1 1 1 in population with unknown status 1 : 0
Number of results 1 1 0 0 0 1 1 1 in population with unknown status 1 : 0
Number of results 0 0 1 0 0 1 1 1 in population with unknown status 1 : 0
Number of results 1 0 1 0 0 1 1 1 in population with unknown status 1 : 0
Number of results 0 1 1 0 0 1 1 1 in population with unknown status 1 : 0
Number of results 1 1 1 0 0 1 1 1 in population with unknown status 1 : 7
Number of results 0 0 0 1 0 1 1 1 in population with unknown status 1 : 0
Number of results 1 0 0 1 0 1 1 1 in population with unknown status 1 : 0
Number of results 0 1 0 1 0 1 1 1 in population with unknown status 1 : 0
Number of results 1 1 0 1 0 1 1 1 in population with unknown status 1 : 0
Number of results 0 0 1 1 0 1 1 1 in population with unknown status 1 : 0
Number of results 1 0 1 1 0 1 1 1 in population with unknown status 1 : 0
Number of results 0 1 1 1 0 1 1 1 in population with unknown status 1 : 0
Number of results 1 1 1 1 0 1 1 1 in population with unknown status 1 : 7
Number of results 0 0 0 0 1 1 1 1 in population with unknown status 1 : 0

Number of results 1 0 0 0 1 1 1 1 in population with unknown status 1 : 0
Number of results 0 1 0 0 1 1 1 1 in population with unknown status 1 : 0
Number of results 1 1 0 0 1 1 1 1 in population with unknown status 1 : 0
Number of results 0 0 1 0 1 1 1 1 in population with unknown status 1 : 0
Number of results 1 0 1 0 1 1 1 1 in population with unknown status 1 : 0
Number of results 0 1 1 0 1 1 1 1 in population with unknown status 1 : 0
Number of results 1 1 1 0 1 1 1 1 in population with unknown status 1 : 0
Number of results 0 0 0 1 1 1 1 1 in population with unknown status 1 : 0
Number of results 1 0 0 1 1 1 1 1 in population with unknown status 1 : 0
Number of results 0 1 0 1 1 1 1 1 in population with unknown status 1 : 0
Number of results 1 1 0 1 1 1 1 1 in population with unknown status 1 : 0
Number of results 0 0 1 1 1 1 1 1 in population with unknown status 1 : 0
Number of results 1 0 1 1 1 1 1 1 in population with unknown status 1 : 0
Number of results 0 1 1 1 1 1 1 1 in population with unknown status 1 : 0
Number of results 1 1 1 1 1 1 1 1 in population with unknown status 1 : 44

population with unknown status 2
Number of results 0 0 0 0 0 0 0 0 in population with unknown status 2 : 144
Number of results 1 0 0 0 0 0 0 0 in population with unknown status 2 : 0
Number of results 0 1 0 0 0 0 0 0 in population with unknown status 2 : 0
Number of results 1 1 0 0 0 0 0 0 in population with unknown status 2 : 0
Number of results 0 0 1 0 0 0 0 0 in population with unknown status 2 : 0
Number of results 1 0 1 0 0 0 0 0 in population with unknown status 2 : 0
Number of results 0 1 1 0 0 0 0 0 in population with unknown status 2 : 0
Number of results 1 1 1 0 0 0 0 0 in population with unknown status 2 : 0
Number of results 0 0 0 1 0 0 0 0 in population with unknown status 2 : 0
Number of results 1 0 0 1 0 0 0 0 in population with unknown status 2 : 0
Number of results 0 1 0 1 0 0 0 0 in population with unknown status 2 : 0
Number of results 1 1 0 1 0 0 0 0 in population with unknown status 2 : 0
Number of results 0 0 1 1 0 0 0 0 in population with unknown status 2 : 0
Number of results 1 0 1 1 0 0 0 0 in population with unknown status 2 : 0
Number of results 0 1 1 1 0 0 0 0 in population with unknown status 2 : 0
Number of results 1 1 1 1 0 0 0 0 in population with unknown status 2 : 0
Number of results 0 0 0 0 1 0 0 0 in population with unknown status 2 : 1
Number of results 1 0 0 0 1 0 0 0 in population with unknown status 2 : 0
Number of results 0 1 0 0 1 0 0 0 in population with unknown status 2 : 0
Number of results 1 1 0 0 1 0 0 0 in population with unknown status 2 : 0
Number of results 0 0 1 0 1 0 0 0 in population with unknown status 2 : 0
Number of results 1 0 1 0 1 0 0 0 in population with unknown status 2 : 0
Number of results 0 1 1 0 1 0 0 0 in population with unknown status 2 : 0
Number of results 1 1 1 0 1 0 0 0 in population with unknown status 2 : 0
Number of results 0 0 0 1 1 0 0 0 in population with unknown status 2 : 0
Number of results 1 0 0 1 1 0 0 0 in population with unknown status 2 : 0
Number of results 0 1 0 1 1 0 0 0 in population with unknown status 2 : 0

Number of results 1 1 0 1 1 0 0 0 in population with unknown status 2 : 0
Number of results 0 0 1 1 1 0 0 0 in population with unknown status 2 : 0
Number of results 1 0 1 1 1 0 0 0 in population with unknown status 2 : 0
Number of results 0 1 1 1 1 0 0 0 in population with unknown status 2 : 0
Number of results 1 1 1 1 1 0 0 0 in population with unknown status 2 : 0
Number of results 0 0 0 0 0 1 0 0 in population with unknown status 2 : 0
Number of results 1 0 0 0 0 1 0 0 in population with unknown status 2 : 0
Number of results 0 1 0 0 0 1 0 0 in population with unknown status 2 : 0
Number of results 1 1 0 0 0 1 0 0 in population with unknown status 2 : 0
Number of results 0 0 1 0 0 1 0 0 in population with unknown status 2 : 0
Number of results 1 0 1 0 0 1 0 0 in population with unknown status 2 : 0
Number of results 0 1 1 0 0 1 0 0 in population with unknown status 2 : 0
Number of results 1 1 1 0 0 1 0 0 in population with unknown status 2 : 0
Number of results 0 0 0 1 0 1 0 0 in population with unknown status 2 : 0
Number of results 1 0 0 1 0 1 0 0 in population with unknown status 2 : 0
Number of results 0 1 0 1 0 1 0 0 in population with unknown status 2 : 0
Number of results 1 1 0 1 0 1 0 0 in population with unknown status 2 : 0
Number of results 0 0 1 1 0 1 0 0 in population with unknown status 2 : 0
Number of results 1 0 1 1 0 1 0 0 in population with unknown status 2 : 0
Number of results 0 1 1 1 0 1 0 0 in population with unknown status 2 : 0
Number of results 1 1 1 1 0 1 0 0 in population with unknown status 2 : 0
Number of results 0 0 0 0 1 1 0 0 in population with unknown status 2 : 0
Number of results 1 0 0 0 1 1 0 0 in population with unknown status 2 : 0
Number of results 0 1 0 0 1 1 0 0 in population with unknown status 2 : 0
Number of results 1 1 0 0 1 1 0 0 in population with unknown status 2 : 0
Number of results 0 0 1 0 1 1 0 0 in population with unknown status 2 : 0
Number of results 1 0 1 0 1 1 0 0 in population with unknown status 2 : 0
Number of results 0 1 1 0 1 1 0 0 in population with unknown status 2 : 0
Number of results 1 1 1 0 1 1 0 0 in population with unknown status 2 : 0
Number of results 0 0 0 1 1 1 0 0 in population with unknown status 2 : 0
Number of results 1 0 0 1 1 1 0 0 in population with unknown status 2 : 0
Number of results 0 1 0 1 1 1 0 0 in population with unknown status 2 : 0
Number of results 1 1 0 1 1 1 0 0 in population with unknown status 2 : 0
Number of results 0 0 1 1 1 1 0 0 in population with unknown status 2 : 0
Number of results 1 0 1 1 1 1 0 0 in population with unknown status 2 : 0
Number of results 0 1 1 1 1 1 0 0 in population with unknown status 2 : 0
Number of results 1 1 1 1 1 1 0 0 in population with unknown status 2 : 0
Number of results 0 0 0 0 0 0 1 0 in population with unknown status 2 : 0
Number of results 1 0 0 0 0 0 1 0 in population with unknown status 2 : 0
Number of results 0 1 0 0 0 0 1 0 in population with unknown status 2 : 0
Number of results 1 1 0 0 0 0 1 0 in population with unknown status 2 : 0
Number of results 0 0 1 0 0 0 1 0 in population with unknown status 2 : 0
Number of results 1 0 1 0 0 0 1 0 in population with unknown status 2 : 0
Number of results 0 1 1 0 0 0 1 0 in population with unknown status 2 : 0

Number of results 1 1 1 0 0 0 1 0 in population with unknown status 2 : 0
Number of results 0 0 0 1 0 0 1 0 in population with unknown status 2 : 0
Number of results 1 0 0 1 0 0 1 0 in population with unknown status 2 : 0
Number of results 0 1 0 1 0 0 1 0 in population with unknown status 2 : 0
Number of results 1 1 0 1 0 0 1 0 in population with unknown status 2 : 0
Number of results 0 0 1 1 0 0 1 0 in population with unknown status 2 : 0
Number of results 1 0 1 1 0 0 1 0 in population with unknown status 2 : 0
Number of results 0 1 1 1 0 0 1 0 in population with unknown status 2 : 0
Number of results 1 1 1 1 0 0 1 0 in population with unknown status 2 : 0
Number of results 0 0 0 0 1 0 1 0 in population with unknown status 2 : 0
Number of results 1 0 0 0 1 0 1 0 in population with unknown status 2 : 0
Number of results 0 1 0 0 1 0 1 0 in population with unknown status 2 : 0
Number of results 1 1 0 0 1 0 1 0 in population with unknown status 2 : 0
Number of results 0 0 1 0 1 0 1 0 in population with unknown status 2 : 0
Number of results 1 0 1 0 1 0 1 0 in population with unknown status 2 : 0
Number of results 0 1 1 0 1 0 1 0 in population with unknown status 2 : 0
Number of results 1 1 1 0 1 0 1 0 in population with unknown status 2 : 0
Number of results 0 0 0 1 1 0 1 0 in population with unknown status 2 : 0
Number of results 1 0 0 1 1 0 1 0 in population with unknown status 2 : 0
Number of results 0 1 0 1 1 0 1 0 in population with unknown status 2 : 0
Number of results 1 1 0 1 1 0 1 0 in population with unknown status 2 : 0
Number of results 0 0 1 1 1 0 1 0 in population with unknown status 2 : 0
Number of results 1 0 1 1 1 0 1 0 in population with unknown status 2 : 0
Number of results 0 1 1 1 1 0 1 0 in population with unknown status 2 : 0
Number of results 1 1 1 1 1 0 1 0 in population with unknown status 2 : 0
Number of results 0 0 0 0 0 1 1 0 in population with unknown status 2 : 0
Number of results 1 0 0 0 0 1 1 0 in population with unknown status 2 : 0
Number of results 0 1 0 0 0 1 1 0 in population with unknown status 2 : 0
Number of results 1 1 0 0 0 1 1 0 in population with unknown status 2 : 0
Number of results 0 0 1 0 0 1 1 0 in population with unknown status 2 : 0
Number of results 1 0 1 0 0 1 1 0 in population with unknown status 2 : 0
Number of results 0 1 1 0 0 1 1 0 in population with unknown status 2 : 0
Number of results 1 1 1 0 0 1 1 0 in population with unknown status 2 : 0
Number of results 0 0 0 1 0 1 1 0 in population with unknown status 2 : 0
Number of results 1 0 0 1 0 1 1 0 in population with unknown status 2 : 0
Number of results 0 1 0 1 0 1 1 0 in population with unknown status 2 : 0
Number of results 1 1 0 1 0 1 1 0 in population with unknown status 2 : 0
Number of results 0 0 1 1 0 1 1 0 in population with unknown status 2 : 2
Number of results 1 0 1 1 0 1 1 0 in population with unknown status 2 : 0
Number of results 0 1 1 1 0 1 1 0 in population with unknown status 2 : 0
Number of results 1 1 1 1 0 1 1 0 in population with unknown status 2 : 0
Number of results 0 0 0 0 1 1 1 0 in population with unknown status 2 : 0
Number of results 1 0 0 0 1 1 1 0 in population with unknown status 2 : 0
Number of results 0 1 0 0 1 1 1 0 in population with unknown status 2 : 0

Number of results 1 1 0 0 1 1 1 0 in population with unknown status 2 : 0
Number of results 0 0 1 0 1 1 1 0 in population with unknown status 2 : 0
Number of results 1 0 1 0 1 1 1 0 in population with unknown status 2 : 0
Number of results 0 1 1 0 1 1 1 0 in population with unknown status 2 : 0
Number of results 1 1 1 0 1 1 1 0 in population with unknown status 2 : 0
Number of results 0 0 0 1 1 1 1 0 in population with unknown status 2 : 0
Number of results 1 0 0 1 1 1 1 0 in population with unknown status 2 : 0
Number of results 0 1 0 1 1 1 1 0 in population with unknown status 2 : 0
Number of results 1 1 0 1 1 1 1 0 in population with unknown status 2 : 0
Number of results 0 0 1 1 1 1 1 0 in population with unknown status 2 : 0
Number of results 1 0 1 1 1 1 1 0 in population with unknown status 2 : 0
Number of results 0 1 1 1 1 1 1 0 in population with unknown status 2 : 0
Number of results 1 1 1 1 1 1 1 0 in population with unknown status 2 : 0
Number of results 0 0 0 0 0 0 0 1 in population with unknown status 2 : 0
Number of results 1 0 0 0 0 0 0 1 in population with unknown status 2 : 0
Number of results 0 1 0 0 0 0 0 1 in population with unknown status 2 : 0
Number of results 1 1 0 0 0 0 0 1 in population with unknown status 2 : 0
Number of results 0 0 1 0 0 0 0 1 in population with unknown status 2 : 0
Number of results 1 0 1 0 0 0 0 1 in population with unknown status 2 : 0
Number of results 0 1 1 0 0 0 0 1 in population with unknown status 2 : 0
Number of results 1 1 1 0 0 0 0 1 in population with unknown status 2 : 0
Number of results 0 0 0 1 0 0 0 1 in population with unknown status 2 : 0
Number of results 1 0 0 1 0 0 0 1 in population with unknown status 2 : 0
Number of results 0 1 0 1 0 0 0 1 in population with unknown status 2 : 0
Number of results 1 1 0 1 0 0 0 1 in population with unknown status 2 : 0
Number of results 0 0 1 1 0 0 0 1 in population with unknown status 2 : 0
Number of results 1 0 1 1 0 0 0 1 in population with unknown status 2 : 0
Number of results 0 1 1 1 0 0 0 1 in population with unknown status 2 : 0
Number of results 1 1 1 1 0 0 0 1 in population with unknown status 2 : 0
Number of results 0 0 0 0 1 0 0 1 in population with unknown status 2 : 0
Number of results 1 0 0 0 1 0 0 1 in population with unknown status 2 : 0
Number of results 0 1 0 0 1 0 0 1 in population with unknown status 2 : 0
Number of results 1 1 0 0 1 0 0 1 in population with unknown status 2 : 0
Number of results 0 0 1 0 1 0 0 1 in population with unknown status 2 : 0
Number of results 1 0 1 0 1 0 0 1 in population with unknown status 2 : 0
Number of results 0 1 1 0 1 0 0 1 in population with unknown status 2 : 0
Number of results 1 1 1 0 1 0 0 1 in population with unknown status 2 : 0
Number of results 0 0 0 1 1 0 0 1 in population with unknown status 2 : 0
Number of results 1 0 0 1 1 0 0 1 in population with unknown status 2 : 0
Number of results 0 1 0 1 1 0 0 1 in population with unknown status 2 : 0
Number of results 1 1 0 1 1 0 0 1 in population with unknown status 2 : 0
Number of results 0 0 1 1 1 0 0 1 in population with unknown status 2 : 0
Number of results 1 0 1 1 1 0 0 1 in population with unknown status 2 : 0
Number of results 0 1 1 1 1 0 0 1 in population with unknown status 2 : 0

Number of results 1 1 1 1 1 0 0 1 in population with unknown status 2 : 0
Number of results 0 0 0 0 0 1 0 1 in population with unknown status 2 : 0
Number of results 1 0 0 0 0 1 0 1 in population with unknown status 2 : 0
Number of results 0 1 0 0 0 1 0 1 in population with unknown status 2 : 4
Number of results 1 1 0 0 0 1 0 1 in population with unknown status 2 : 0
Number of results 0 0 1 0 0 1 0 1 in population with unknown status 2 : 0
Number of results 1 0 1 0 0 1 0 1 in population with unknown status 2 : 0
Number of results 0 1 1 0 0 1 0 1 in population with unknown status 2 : 0
Number of results 1 1 1 0 0 1 0 1 in population with unknown status 2 : 0
Number of results 0 0 0 1 0 1 0 1 in population with unknown status 2 : 0
Number of results 1 0 0 1 0 1 0 1 in population with unknown status 2 : 0
Number of results 0 1 0 1 0 1 0 1 in population with unknown status 2 : 0
Number of results 1 1 0 1 0 1 0 1 in population with unknown status 2 : 0
Number of results 0 0 1 1 0 1 0 1 in population with unknown status 2 : 0
Number of results 1 0 1 1 0 1 0 1 in population with unknown status 2 : 0
Number of results 0 1 1 1 0 1 0 1 in population with unknown status 2 : 0
Number of results 1 1 1 1 0 1 0 1 in population with unknown status 2 : 0
Number of results 0 0 0 0 1 1 0 1 in population with unknown status 2 : 0
Number of results 1 0 0 0 1 1 0 1 in population with unknown status 2 : 0
Number of results 0 1 0 0 1 1 0 1 in population with unknown status 2 : 0
Number of results 1 1 0 0 1 1 0 1 in population with unknown status 2 : 0
Number of results 0 0 1 0 1 1 0 1 in population with unknown status 2 : 0
Number of results 1 0 1 0 1 1 0 1 in population with unknown status 2 : 0
Number of results 0 1 1 0 1 1 0 1 in population with unknown status 2 : 0
Number of results 1 1 1 0 1 1 0 1 in population with unknown status 2 : 0
Number of results 0 0 0 1 1 1 0 1 in population with unknown status 2 : 0
Number of results 1 0 0 1 1 1 0 1 in population with unknown status 2 : 0
Number of results 0 1 0 1 1 1 0 1 in population with unknown status 2 : 0
Number of results 1 1 0 1 1 1 0 1 in population with unknown status 2 : 0
Number of results 0 0 1 1 1 1 0 1 in population with unknown status 2 : 0
Number of results 1 0 1 1 1 1 0 1 in population with unknown status 2 : 0
Number of results 0 1 1 1 1 1 0 1 in population with unknown status 2 : 0
Number of results 1 1 1 1 1 1 0 1 in population with unknown status 2 : 0
Number of results 0 0 0 0 0 0 1 1 in population with unknown status 2 : 2
Number of results 1 0 0 0 0 0 1 1 in population with unknown status 2 : 0
Number of results 0 1 0 0 0 0 1 1 in population with unknown status 2 : 0
Number of results 1 1 0 0 0 0 1 1 in population with unknown status 2 : 0
Number of results 0 0 1 0 0 0 1 1 in population with unknown status 2 : 9
Number of results 1 0 1 0 0 0 1 1 in population with unknown status 2 : 0
Number of results 0 1 1 0 0 0 1 1 in population with unknown status 2 : 0
Number of results 1 1 1 0 0 0 1 1 in population with unknown status 2 : 0
Number of results 0 0 0 1 0 0 1 1 in population with unknown status 2 : 0
Number of results 1 0 0 1 0 0 1 1 in population with unknown status 2 : 0
Number of results 0 1 0 1 0 0 1 1 in population with unknown status 2 : 0

Number of results 1 1 0 1 0 0 1 1 in population with unknown status 2 : 0
Number of results 0 0 1 1 0 0 1 1 in population with unknown status 2 : 0
Number of results 1 0 1 1 0 0 1 1 in population with unknown status 2 : 0
Number of results 0 1 1 1 0 0 1 1 in population with unknown status 2 : 0
Number of results 1 1 1 1 0 0 1 1 in population with unknown status 2 : 0
Number of results 0 0 0 0 1 0 1 1 in population with unknown status 2 : 0
Number of results 1 0 0 0 1 0 1 1 in population with unknown status 2 : 0
Number of results 0 1 0 0 1 0 1 1 in population with unknown status 2 : 0
Number of results 1 1 0 0 1 0 1 1 in population with unknown status 2 : 0
Number of results 0 0 1 0 1 0 1 1 in population with unknown status 2 : 0
Number of results 1 0 1 0 1 0 1 1 in population with unknown status 2 : 0
Number of results 0 1 1 0 1 0 1 1 in population with unknown status 2 : 0
Number of results 1 1 1 0 1 0 1 1 in population with unknown status 2 : 0
Number of results 0 0 0 1 1 0 1 1 in population with unknown status 2 : 0
Number of results 1 0 0 1 1 0 1 1 in population with unknown status 2 : 0
Number of results 0 1 0 1 1 0 1 1 in population with unknown status 2 : 0
Number of results 1 1 0 1 1 0 1 1 in population with unknown status 2 : 0
Number of results 0 0 1 1 1 0 1 1 in population with unknown status 2 : 0
Number of results 1 0 1 1 1 0 1 1 in population with unknown status 2 : 0
Number of results 0 1 1 1 1 0 1 1 in population with unknown status 2 : 0
Number of results 1 1 1 1 1 0 1 1 in population with unknown status 2 : 0
Number of results 0 0 0 0 0 1 1 1 in population with unknown status 2 : 0
Number of results 1 0 0 0 0 1 1 1 in population with unknown status 2 : 0
Number of results 0 1 0 0 0 1 1 1 in population with unknown status 2 : 0
Number of results 1 1 0 0 0 1 1 1 in population with unknown status 2 : 0
Number of results 0 0 1 0 0 1 1 1 in population with unknown status 2 : 0
Number of results 1 0 1 0 0 1 1 1 in population with unknown status 2 : 0
Number of results 0 1 1 0 0 1 1 1 in population with unknown status 2 : 0
Number of results 1 1 1 0 0 1 1 1 in population with unknown status 2 : 1
Number of results 0 0 0 1 0 1 1 1 in population with unknown status 2 : 0
Number of results 1 0 0 1 0 1 1 1 in population with unknown status 2 : 1
Number of results 0 1 0 1 0 1 1 1 in population with unknown status 2 : 0
Number of results 1 1 0 1 0 1 1 1 in population with unknown status 2 : 0
Number of results 0 0 1 1 0 1 1 1 in population with unknown status 2 : 0
Number of results 1 0 1 1 0 1 1 1 in population with unknown status 2 : 0
Number of results 0 1 1 1 0 1 1 1 in population with unknown status 2 : 0
Number of results 1 1 1 1 0 1 1 1 in population with unknown status 2 : 10
Number of results 0 0 0 0 1 1 1 1 in population with unknown status 2 : 0
Number of results 1 0 0 0 1 1 1 1 in population with unknown status 2 : 0
Number of results 0 1 0 0 1 1 1 1 in population with unknown status 2 : 0
Number of results 1 1 0 0 1 1 1 1 in population with unknown status 2 : 0
Number of results 0 0 1 0 1 1 1 1 in population with unknown status 2 : 0
Number of results 1 0 1 0 1 1 1 1 in population with unknown status 2 : 0
Number of results 0 1 1 0 1 1 1 1 in population with unknown status 2 : 0

Number of results 1 1 1 0 1 1 1 1 in population with unknown status 2 : 0
Number of results 0 0 0 1 1 1 1 1 in population with unknown status 2 : 0
Number of results 1 0 0 1 1 1 1 1 in population with unknown status 2 : 0
Number of results 0 1 0 1 1 1 1 1 in population with unknown status 2 : 0
Number of results 1 1 0 1 1 1 1 1 in population with unknown status 2 : 0
Number of results 0 0 1 1 1 1 1 1 in population with unknown status 2 : 0
Number of results 1 0 1 1 1 1 1 1 in population with unknown status 2 : 0
Number of results 0 1 1 1 1 1 1 1 in population with unknown status 2 : 0
Number of results 1 1 1 1 1 1 1 1 in population with unknown status 2 : 31

DATA SUMMARY
2 Population(s); 8 Tests; 0 Reference Population(s)

df: 510 ; parameters: 18

	test1	test2	test3	test4	test5	test6	test7	test8	pop1	pop2	RefInd	RefInf
1	0	0	0	0	0	0	0	0	254	144	0	0
2	1	0	0	0	0	0	0	0	0	0	0	0
3	0	1	0	0	0	0	0	0	0	0	0	0
4	1	1	0	0	0	0	0	0	0	0	0	0
5	0	0	1	0	0	0	0	0	0	0	0	0
6	1	0	1	0	0	0	0	0	0	0	0	0
7	0	1	1	0	0	0	0	0	0	0	0	0
8	1	1	1	0	0	0	0	0	0	0	0	0
9	0	0	0	1	0	0	0	0	0	0	0	0
10	1	0	0	1	0	0	0	0	0	0	0	0
11	0	1	0	1	0	0	0	0	0	0	0	0
12	1	1	0	1	0	0	0	0	0	0	0	0
13	0	0	1	1	0	0	0	0	0	0	0	0
14	1	0	1	1	0	0	0	0	0	0	0	0
15	0	1	1	1	0	0	0	0	0	0	0	0
16	1	1	1	1	0	0	0	0	0	0	0	0
17	0	0	0	0	1	0	0	0	0	1	0	0
18	1	0	0	0	1	0	0	0	0	0	0	0
19	0	1	0	0	1	0	0	0	0	0	0	0
20	1	1	0	0	1	0	0	0	0	0	0	0
21	0	0	1	0	1	0	0	0	0	0	0	0
22	1	0	1	0	1	0	0	0	0	0	0	0
23	0	1	1	0	1	0	0	0	0	0	0	0
24	1	1	1	0	1	0	0	0	1	0	0	0
25	0	0	0	1	1	0	0	0	0	0	0	0
26	1	0	0	1	1	0	0	0	0	0	0	0
27	0	1	0	1	1	0	0	0	0	0	0	0

28	1	1	0	1	1	0	0	0	0	0	0	0
29	0	0	1	1	1	0	0	0	0	0	0	0
30	1	0	1	1	1	0	0	0	0	0	0	0
31	0	1	1	1	1	0	0	0	0	0	0	0
32	1	1	1	1	1	0	0	0	0	0	0	0
33	0	0	0	0	0	1	0	0	0	0	0	0
34	1	0	0	0	0	1	0	0	0	0	0	0
35	0	1	0	0	0	1	0	0	0	0	0	0
36	1	1	0	0	0	1	0	0	0	0	0	0
37	0	0	1	0	0	1	0	0	0	0	0	0
38	1	0	1	0	0	1	0	0	0	0	0	0
39	0	1	1	0	0	1	0	0	0	0	0	0
40	1	1	1	0	0	1	0	0	0	0	0	0
41	0	0	0	1	0	1	0	0	0	0	0	0
42	1	0	0	1	0	1	0	0	0	0	0	0
43	0	1	0	1	0	1	0	0	0	0	0	0
44	1	1	0	1	0	1	0	0	0	0	0	0
45	0	0	1	1	0	1	0	0	0	0	0	0
46	1	0	1	1	0	1	0	0	0	0	0	0
47	0	1	1	1	0	1	0	0	0	0	0	0
48	1	1	1	1	0	1	0	0	0	0	0	0
49	0	0	0	0	1	1	0	0	0	0	0	0
50	1	0	0	0	1	1	0	0	0	0	0	0
51	0	1	0	0	1	1	0	0	0	0	0	0
52	1	1	0	0	1	1	0	0	0	0	0	0
53	0	0	1	0	1	1	0	0	0	0	0	0
54	1	0	1	0	1	1	0	0	0	0	0	0
55	0	1	1	0	1	1	0	0	0	0	0	0
56	1	1	1	0	1	1	0	0	0	0	0	0
57	0	0	0	1	1	1	0	0	0	0	0	0
58	1	0	0	1	1	1	0	0	0	0	0	0
59	0	1	0	1	1	1	0	0	0	0	0	0
60	1	1	0	1	1	1	0	0	0	0	0	0
61	0	0	1	1	1	1	0	0	0	0	0	0
62	1	0	1	1	1	1	0	0	0	0	0	0
63	0	1	1	1	1	1	0	0	0	0	0	0
64	1	1	1	1	1	1	0	0	0	0	0	0
65	0	0	0	0	0	0	1	0	0	0	0	0
66	1	0	0	0	0	0	1	0	0	0	0	0
67	0	1	0	0	0	0	1	0	0	0	0	0
68	1	1	0	0	0	0	1	0	0	0	0	0
69	0	0	1	0	0	0	1	0	0	0	0	0
70	1	0	1	0	0	0	1	0	0	0	0	0
71	0	1	1	0	0	0	1	0	0	0	0	0

72	1	1	1	0	0	0	1	0	0	0	0	0
73	0	0	0	1	0	0	1	0	0	0	0	0
74	1	0	0	1	0	0	1	0	0	0	0	0
75	0	1	0	1	0	0	1	0	0	0	0	0
76	1	1	0	1	0	0	1	0	0	0	0	0
77	0	0	1	1	0	0	1	0	0	0	0	0
78	1	0	1	1	0	0	1	0	0	0	0	0
79	0	1	1	1	0	0	1	0	0	0	0	0
80	1	1	1	1	0	0	1	0	0	0	0	0
81	0	0	0	0	1	0	1	0	0	0	0	0
82	1	0	0	0	1	0	1	0	0	0	0	0
83	0	1	0	0	1	0	1	0	0	0	0	0
84	1	1	0	0	1	0	1	0	0	0	0	0
85	0	0	1	0	1	0	1	0	0	0	0	0
86	1	0	1	0	1	0	1	0	0	0	0	0
87	0	1	1	0	1	0	1	0	0	0	0	0
88	1	1	1	0	1	0	1	0	0	0	0	0
89	0	0	0	1	1	0	1	0	0	0	0	0
90	1	0	0	1	1	0	1	0	0	0	0	0
91	0	1	0	1	1	0	1	0	0	0	0	0
92	1	1	0	1	1	0	1	0	0	0	0	0
93	0	0	1	1	1	0	1	0	0	0	0	0
94	1	0	1	1	1	0	1	0	0	0	0	0
95	0	1	1	1	1	0	1	0	0	0	0	0
96	1	1	1	1	1	0	1	0	0	0	0	0
97	0	0	0	0	0	1	1	0	0	0	0	0
98	1	0	0	0	0	1	1	0	0	0	0	0
99	0	1	0	0	0	1	1	0	0	0	0	0
100	1	1	0	0	0	1	1	0	0	0	0	0
101	0	0	1	0	0	1	1	0	0	0	0	0
102	1	0	1	0	0	1	1	0	0	0	0	0
103	0	1	1	0	0	1	1	0	0	0	0	0
104	1	1	1	0	0	1	1	0	0	0	0	0
105	0	0	0	1	0	1	1	0	0	0	0	0
106	1	0	0	1	0	1	1	0	0	0	0	0
107	0	1	0	1	0	1	1	0	0	0	0	0
108	1	1	0	1	0	1	1	0	0	0	0	0
109	0	0	1	1	0	1	1	0	0	2	0	0
110	1	0	1	1	0	1	1	0	0	0	0	0
111	0	1	1	1	0	1	1	0	1	0	0	0
112	1	1	1	1	0	1	1	0	0	0	0	0
113	0	0	0	0	1	1	1	0	0	0	0	0
114	1	0	0	0	1	1	1	0	0	0	0	0
115	0	1	0	0	1	1	1	0	0	0	0	0

116	1	1	0	0	1	1	1	0	0	0	0	0
117	0	0	1	0	1	1	1	0	0	0	0	0
118	1	0	1	0	1	1	1	0	0	0	0	0
119	0	1	1	0	1	1	1	0	0	0	0	0
120	1	1	1	0	1	1	1	0	0	0	0	0
121	0	0	0	1	1	1	1	0	1	0	0	0
122	1	0	0	1	1	1	1	0	0	0	0	0
123	0	1	0	1	1	1	1	0	0	0	0	0
124	1	1	0	1	1	1	1	0	0	0	0	0
125	0	0	1	1	1	1	1	0	0	0	0	0
126	1	0	1	1	1	1	1	0	0	0	0	0
127	0	1	1	1	1	1	1	0	2	0	0	0
128	1	1	1	1	1	1	1	0	0	0	0	0
129	0	0	0	0	0	0	0	1	0	0	0	0
130	1	0	0	0	0	0	0	1	0	0	0	0
131	0	1	0	0	0	0	0	1	0	0	0	0
132	1	1	0	0	0	0	0	1	0	0	0	0
133	0	0	1	0	0	0	0	1	0	0	0	0
134	1	0	1	0	0	0	0	1	0	0	0	0
135	0	1	1	0	0	0	0	1	0	0	0	0
136	1	1	1	0	0	0	0	1	0	0	0	0
137	0	0	0	1	0	0	0	1	0	0	0	0
138	1	0	0	1	0	0	0	1	0	0	0	0
139	0	1	0	1	0	0	0	1	0	0	0	0
140	1	1	0	1	0	0	0	1	0	0	0	0
141	0	0	1	1	0	0	0	1	0	0	0	0
142	1	0	1	1	0	0	0	1	0	0	0	0
143	0	1	1	1	0	0	0	1	0	0	0	0
144	1	1	1	1	0	0	0	1	0	0	0	0
145	0	0	0	0	1	0	0	1	0	0	0	0
146	1	0	0	0	1	0	0	1	0	0	0	0
147	0	1	0	0	1	0	0	1	0	0	0	0
148	1	1	0	0	1	0	0	1	0	0	0	0
149	0	0	1	0	1	0	0	1	0	0	0	0
150	1	0	1	0	1	0	0	1	0	0	0	0
151	0	1	1	0	1	0	0	1	0	0	0	0
152	1	1	1	0	1	0	0	1	0	0	0	0
153	0	0	0	1	1	0	0	1	0	0	0	0
154	1	0	0	1	1	0	0	1	0	0	0	0
155	0	1	0	1	1	0	0	1	0	0	0	0
156	1	1	0	1	1	0	0	1	0	0	0	0
157	0	0	1	1	1	0	0	1	0	0	0	0
158	1	0	1	1	1	0	0	1	0	0	0	0
159	0	1	1	1	1	0	0	1	0	0	0	0

160	1	1	1	1	1	0	0	1	0	0	0	0
161	0	0	0	0	0	1	0	1	0	0	0	0
162	1	0	0	0	0	1	0	1	0	0	0	0
163	0	1	0	0	0	1	0	1	6	4	0	0
164	1	1	0	0	0	1	0	1	0	0	0	0
165	0	0	1	0	0	1	0	1	0	0	0	0
166	1	0	1	0	0	1	0	1	0	0	0	0
167	0	1	1	0	0	1	0	1	0	0	0	0
168	1	1	1	0	0	1	0	1	0	0	0	0
169	0	0	0	1	0	1	0	1	0	0	0	0
170	1	0	0	1	0	1	0	1	0	0	0	0
171	0	1	0	1	0	1	0	1	0	0	0	0
172	1	1	0	1	0	1	0	1	0	0	0	0
173	0	0	1	1	0	1	0	1	0	0	0	0
174	1	0	1	1	0	1	0	1	0	0	0	0
175	0	1	1	1	0	1	0	1	0	0	0	0
176	1	1	1	1	0	1	0	1	0	0	0	0
177	0	0	0	0	1	1	0	1	0	0	0	0
178	1	0	0	0	1	1	0	1	0	0	0	0
179	0	1	0	0	1	1	0	1	0	0	0	0
180	1	1	0	0	1	1	0	1	0	0	0	0
181	0	0	1	0	1	1	0	1	0	0	0	0
182	1	0	1	0	1	1	0	1	0	0	0	0
183	0	1	1	0	1	1	0	1	0	0	0	0
184	1	1	1	0	1	1	0	1	0	0	0	0
185	0	0	0	1	1	1	0	1	0	0	0	0
186	1	0	0	1	1	1	0	1	0	0	0	0
187	0	1	0	1	1	1	0	1	0	0	0	0
188	1	1	0	1	1	1	0	1	0	0	0	0
189	0	0	1	1	1	1	0	1	0	0	0	0
190	1	0	1	1	1	1	0	1	0	0	0	0
191	0	1	1	1	1	1	0	1	0	0	0	0
192	1	1	1	1	1	1	0	1	0	0	0	0
193	0	0	0	0	0	0	1	1	4	2	0	0
194	1	0	0	0	0	0	1	1	0	0	0	0
195	0	1	0	0	0	0	1	1	0	0	0	0
196	1	1	0	0	0	0	1	1	0	0	0	0
197	0	0	1	0	0	0	1	1	11	9	0	0
198	1	0	1	0	0	0	1	1	0	0	0	0
199	0	1	1	0	0	0	1	1	0	0	0	0
200	1	1	1	0	0	0	1	1	0	0	0	0
201	0	0	0	1	0	0	1	1	0	0	0	0
202	1	0	0	1	0	0	1	1	0	0	0	0
203	0	1	0	1	0	0	1	1	0	0	0	0

204	1	1	0	1	0	0	1	1	0	0	0	0
205	0	0	1	1	0	0	1	1	0	0	0	0
206	1	0	1	1	0	0	1	1	0	0	0	0
207	0	1	1	1	0	0	1	1	0	0	0	0
208	1	1	1	1	0	0	1	1	0	0	0	0
209	0	0	0	0	1	0	1	1	0	0	0	0
210	1	0	0	0	1	0	1	1	0	0	0	0
211	0	1	0	0	1	0	1	1	0	0	0	0
212	1	1	0	0	1	0	1	1	0	0	0	0
213	0	0	1	0	1	0	1	1	0	0	0	0
214	1	0	1	0	1	0	1	1	0	0	0	0
215	0	1	1	0	1	0	1	1	0	0	0	0
216	1	1	1	0	1	0	1	1	0	0	0	0
217	0	0	0	1	1	0	1	1	0	0	0	0
218	1	0	0	1	1	0	1	1	0	0	0	0
219	0	1	0	1	1	0	1	1	0	0	0	0
220	1	1	0	1	1	0	1	1	0	0	0	0
221	0	0	1	1	1	0	1	1	0	0	0	0
222	1	0	1	1	1	0	1	1	0	0	0	0
223	0	1	1	1	1	0	1	1	0	0	0	0
224	1	1	1	1	1	0	1	1	0	0	0	0
225	0	0	0	0	0	1	1	1	0	0	0	0
226	1	0	0	0	0	1	1	1	0	0	0	0
227	0	1	0	0	0	1	1	1	0	0	0	0
228	1	1	0	0	0	1	1	1	0	0	0	0
229	0	0	1	0	0	1	1	1	0	0	0	0
230	1	0	1	0	0	1	1	1	0	0	0	0
231	0	1	1	0	0	1	1	1	0	0	0	0
232	1	1	1	0	0	1	1	1	7	1	0	0
233	0	0	0	1	0	1	1	1	0	0	0	0
234	1	0	0	1	0	1	1	1	0	1	0	0
235	0	1	0	1	0	1	1	1	0	0	0	0
236	1	1	0	1	0	1	1	1	0	0	0	0
237	0	0	1	1	0	1	1	1	0	0	0	0
238	1	0	1	1	0	1	1	1	0	0	0	0
239	0	1	1	1	0	1	1	1	0	0	0	0
240	1	1	1	1	0	1	1	1	7	10	0	0
241	0	0	0	0	1	1	1	1	0	0	0	0
242	1	0	0	0	1	1	1	1	0	0	0	0
243	0	1	0	0	1	1	1	1	0	0	0	0
244	1	1	0	0	1	1	1	1	0	0	0	0
245	0	0	1	0	1	1	1	1	0	0	0	0
246	1	0	1	0	1	1	1	1	0	0	0	0
247	0	1	1	0	1	1	1	1	0	0	0	0

248	1	1	1	0	1	1	1	1	0	0	0	0
249	0	0	0	1	1	1	1	1	0	0	0	0
250	1	0	0	1	1	1	1	1	0	0	0	0
251	0	1	0	1	1	1	1	1	0	0	0	0
252	1	1	0	1	1	1	1	1	0	0	0	0
253	0	0	1	1	1	1	1	1	0	0	0	0
254	1	0	1	1	1	1	1	1	0	0	0	0
255	0	1	1	1	1	1	1	1	0	0	0	0
256	1	1	1	1	1	1	1	1	44	31	0	0

Is it OK ?
No : Enter 0
Yes : Enter 1
1

Default Best Guess:

	pre1	pre2	Sp1	Sp2	Sp3	Sp4	Sp5	Sp6	Sp7	Sp8	Se1	Se2	Se3	Se4
Best Guess	0.2	0.2	0.95	0.95	0.95	0.95	0.95	0.95	0.95	0.95	0.8	0.8	0.8	0.8

	Se5	Se6	Se7	Se8
Best Guess	0.8	0.8	0.8	0.8

Do you have Best Guess?
No : Enter 0
Yes : Enter 1
0

Would you like Bootstrap Confidence intervals
(CAUTION: this may be time consumming. It is recommended to ask for it in a second step!)
No : Enter 0
Yes : Enter 1
1
How many iterations? (between 50 and 5000)
5000

DATA SUMMARY
2 Population(s); 8 Tests; 0 Reference Population(s)

df: 510 ; parameters: 18

	test1	test2	test3	test4	test5	test6	test7	test8	pop1	pop2	RefInd	RefInf
1	0	0	0	0	0	0	0	0	254	144	0	0
2	1	0	0	0	0	0	0	0	0	0	0	0

3	0	1	0	0	0	0	0	0	0	0	0	0
4	1	1	0	0	0	0	0	0	0	0	0	0
5	0	0	1	0	0	0	0	0	0	0	0	0
6	1	0	1	0	0	0	0	0	0	0	0	0
7	0	1	1	0	0	0	0	0	0	0	0	0
8	1	1	1	0	0	0	0	0	0	0	0	0
9	0	0	0	1	0	0	0	0	0	0	0	0
10	1	0	0	1	0	0	0	0	0	0	0	0
11	0	1	0	1	0	0	0	0	0	0	0	0
12	1	1	0	1	0	0	0	0	0	0	0	0
13	0	0	1	1	0	0	0	0	0	0	0	0
14	1	0	1	1	0	0	0	0	0	0	0	0
15	0	1	1	1	0	0	0	0	0	0	0	0
16	1	1	1	1	0	0	0	0	0	0	0	0
17	0	0	0	0	1	0	0	0	0	1	0	0
18	1	0	0	0	1	0	0	0	0	0	0	0
19	0	1	0	0	1	0	0	0	0	0	0	0
20	1	1	0	0	1	0	0	0	0	0	0	0
21	0	0	1	0	1	0	0	0	0	0	0	0
22	1	0	1	0	1	0	0	0	0	0	0	0
23	0	1	1	0	1	0	0	0	0	0	0	0
24	1	1	1	0	1	0	0	0	1	0	0	0
25	0	0	0	1	1	0	0	0	0	0	0	0
26	1	0	0	1	1	0	0	0	0	0	0	0
27	0	1	0	1	1	0	0	0	0	0	0	0
28	1	1	0	1	1	0	0	0	0	0	0	0
29	0	0	1	1	1	0	0	0	0	0	0	0
30	1	0	1	1	1	0	0	0	0	0	0	0
31	0	1	1	1	1	0	0	0	0	0	0	0
32	1	1	1	1	1	0	0	0	0	0	0	0
33	0	0	0	0	0	1	0	0	0	0	0	0
34	1	0	0	0	0	1	0	0	0	0	0	0
35	0	1	0	0	0	1	0	0	0	0	0	0
36	1	1	0	0	0	1	0	0	0	0	0	0
37	0	0	1	0	0	1	0	0	0	0	0	0
38	1	0	1	0	0	1	0	0	0	0	0	0
39	0	1	1	0	0	1	0	0	0	0	0	0
40	1	1	1	0	0	1	0	0	0	0	0	0
41	0	0	0	1	0	1	0	0	0	0	0	0
42	1	0	0	1	0	1	0	0	0	0	0	0
43	0	1	0	1	0	1	0	0	0	0	0	0
44	1	1	0	1	0	1	0	0	0	0	0	0
45	0	0	1	1	0	1	0	0	0	0	0	0
46	1	0	1	1	0	1	0	0	0	0	0	0

47	0	1	1	1	0	1	0	0	0	0	0	0
48	1	1	1	1	0	1	0	0	0	0	0	0
49	0	0	0	0	1	1	0	0	0	0	0	0
50	1	0	0	0	1	1	0	0	0	0	0	0
51	0	1	0	0	1	1	0	0	0	0	0	0
52	1	1	0	0	1	1	0	0	0	0	0	0
53	0	0	1	0	1	1	0	0	0	0	0	0
54	1	0	1	0	1	1	0	0	0	0	0	0
55	0	1	1	0	1	1	0	0	0	0	0	0
56	1	1	1	0	1	1	0	0	0	0	0	0
57	0	0	0	1	1	1	0	0	0	0	0	0
58	1	0	0	1	1	1	0	0	0	0	0	0
59	0	1	0	1	1	1	0	0	0	0	0	0
60	1	1	0	1	1	1	0	0	0	0	0	0
61	0	0	1	1	1	1	0	0	0	0	0	0
62	1	0	1	1	1	1	0	0	0	0	0	0
63	0	1	1	1	1	1	0	0	0	0	0	0
64	1	1	1	1	1	1	0	0	0	0	0	0
65	0	0	0	0	0	0	1	0	0	0	0	0
66	1	0	0	0	0	0	1	0	0	0	0	0
67	0	1	0	0	0	0	1	0	0	0	0	0
68	1	1	0	0	0	0	1	0	0	0	0	0
69	0	0	1	0	0	0	1	0	0	0	0	0
70	1	0	1	0	0	0	1	0	0	0	0	0
71	0	1	1	0	0	0	1	0	0	0	0	0
72	1	1	1	0	0	0	1	0	0	0	0	0
73	0	0	0	1	0	0	1	0	0	0	0	0
74	1	0	0	1	0	0	1	0	0	0	0	0
75	0	1	0	1	0	0	1	0	0	0	0	0
76	1	1	0	1	0	0	1	0	0	0	0	0
77	0	0	1	1	0	0	1	0	0	0	0	0
78	1	0	1	1	0	0	1	0	0	0	0	0
79	0	1	1	1	0	0	1	0	0	0	0	0
80	1	1	1	1	0	0	1	0	0	0	0	0
81	0	0	0	0	1	0	1	0	0	0	0	0
82	1	0	0	0	1	0	1	0	0	0	0	0
83	0	1	0	0	1	0	1	0	0	0	0	0
84	1	1	0	0	1	0	1	0	0	0	0	0
85	0	0	1	0	1	0	1	0	0	0	0	0
86	1	0	1	0	1	0	1	0	0	0	0	0
87	0	1	1	0	1	0	1	0	0	0	0	0
88	1	1	1	0	1	0	1	0	0	0	0	0
89	0	0	0	1	1	0	1	0	0	0	0	0
90	1	0	0	1	1	0	1	0	0	0	0	0

91	0	1	0	1	1	0	1	0	0	0	0	0
92	1	1	0	1	1	0	1	0	0	0	0	0
93	0	0	1	1	1	0	1	0	0	0	0	0
94	1	0	1	1	1	0	1	0	0	0	0	0
95	0	1	1	1	1	0	1	0	0	0	0	0
96	1	1	1	1	1	0	1	0	0	0	0	0
97	0	0	0	0	0	1	1	0	0	0	0	0
98	1	0	0	0	0	1	1	0	0	0	0	0
99	0	1	0	0	0	1	1	0	0	0	0	0
100	1	1	0	0	0	1	1	0	0	0	0	0
101	0	0	1	0	0	1	1	0	0	0	0	0
102	1	0	1	0	0	1	1	0	0	0	0	0
103	0	1	1	0	0	1	1	0	0	0	0	0
104	1	1	1	0	0	1	1	0	0	0	0	0
105	0	0	0	1	0	1	1	0	0	0	0	0
106	1	0	0	1	0	1	1	0	0	0	0	0
107	0	1	0	1	0	1	1	0	0	0	0	0
108	1	1	0	1	0	1	1	0	0	0	0	0
109	0	0	1	1	0	1	1	0	0	2	0	0
110	1	0	1	1	0	1	1	0	0	0	0	0
111	0	1	1	1	0	1	1	0	1	0	0	0
112	1	1	1	1	0	1	1	0	0	0	0	0
113	0	0	0	0	1	1	1	0	0	0	0	0
114	1	0	0	0	1	1	1	0	0	0	0	0
115	0	1	0	0	1	1	1	0	0	0	0	0
116	1	1	0	0	1	1	1	0	0	0	0	0
117	0	0	1	0	1	1	1	0	0	0	0	0
118	1	0	1	0	1	1	1	0	0	0	0	0
119	0	1	1	0	1	1	1	0	0	0	0	0
120	1	1	1	0	1	1	1	0	0	0	0	0
121	0	0	0	1	1	1	1	0	1	0	0	0
122	1	0	0	1	1	1	1	0	0	0	0	0
123	0	1	0	1	1	1	1	0	0	0	0	0
124	1	1	0	1	1	1	1	0	0	0	0	0
125	0	0	1	1	1	1	1	0	0	0	0	0
126	1	0	1	1	1	1	1	0	0	0	0	0
127	0	1	1	1	1	1	1	0	2	0	0	0
128	1	1	1	1	1	1	1	0	0	0	0	0
129	0	0	0	0	0	0	0	1	0	0	0	0
130	1	0	0	0	0	0	0	1	0	0	0	0
131	0	1	0	0	0	0	0	1	0	0	0	0
132	1	1	0	0	0	0	0	1	0	0	0	0
133	0	0	1	0	0	0	0	1	0	0	0	0
134	1	0	1	0	0	0	0	1	0	0	0	0

135	0	1	1	0	0	0	0	1	0	0	0	0
136	1	1	1	0	0	0	0	1	0	0	0	0
137	0	0	0	1	0	0	0	1	0	0	0	0
138	1	0	0	1	0	0	0	1	0	0	0	0
139	0	1	0	1	0	0	0	1	0	0	0	0
140	1	1	0	1	0	0	0	1	0	0	0	0
141	0	0	1	1	0	0	0	1	0	0	0	0
142	1	0	1	1	0	0	0	1	0	0	0	0
143	0	1	1	1	0	0	0	1	0	0	0	0
144	1	1	1	1	0	0	0	1	0	0	0	0
145	0	0	0	0	1	0	0	1	0	0	0	0
146	1	0	0	0	1	0	0	1	0	0	0	0
147	0	1	0	0	1	0	0	1	0	0	0	0
148	1	1	0	0	1	0	0	1	0	0	0	0
149	0	0	1	0	1	0	0	1	0	0	0	0
150	1	0	1	0	1	0	0	1	0	0	0	0
151	0	1	1	0	1	0	0	1	0	0	0	0
152	1	1	1	0	1	0	0	1	0	0	0	0
153	0	0	0	1	1	0	0	1	0	0	0	0
154	1	0	0	1	1	0	0	1	0	0	0	0
155	0	1	0	1	1	0	0	1	0	0	0	0
156	1	1	0	1	1	0	0	1	0	0	0	0
157	0	0	1	1	1	0	0	1	0	0	0	0
158	1	0	1	1	1	0	0	1	0	0	0	0
159	0	1	1	1	1	0	0	1	0	0	0	0
160	1	1	1	1	1	0	0	1	0	0	0	0
161	0	0	0	0	0	1	0	1	0	0	0	0
162	1	0	0	0	0	1	0	1	0	0	0	0
163	0	1	0	0	0	1	0	1	6	4	0	0
164	1	1	0	0	0	1	0	1	0	0	0	0
165	0	0	1	0	0	1	0	1	0	0	0	0
166	1	0	1	0	0	1	0	1	0	0	0	0
167	0	1	1	0	0	1	0	1	0	0	0	0
168	1	1	1	0	0	1	0	1	0	0	0	0
169	0	0	0	1	0	1	0	1	0	0	0	0
170	1	0	0	1	0	1	0	1	0	0	0	0
171	0	1	0	1	0	1	0	1	0	0	0	0
172	1	1	0	1	0	1	0	1	0	0	0	0
173	0	0	1	1	0	1	0	1	0	0	0	0
174	1	0	1	1	0	1	0	1	0	0	0	0
175	0	1	1	1	0	1	0	1	0	0	0	0
176	1	1	1	1	0	1	0	1	0	0	0	0
177	0	0	0	0	1	1	0	1	0	0	0	0
178	1	0	0	0	1	1	0	1	0	0	0	0

179	0	1	0	0	1	1	0	1	0	0	0	0
180	1	1	0	0	1	1	0	1	0	0	0	0
181	0	0	1	0	1	1	0	1	0	0	0	0
182	1	0	1	0	1	1	0	1	0	0	0	0
183	0	1	1	0	1	1	0	1	0	0	0	0
184	1	1	1	0	1	1	0	1	0	0	0	0
185	0	0	0	1	1	1	0	1	0	0	0	0
186	1	0	0	1	1	1	0	1	0	0	0	0
187	0	1	0	1	1	1	0	1	0	0	0	0
188	1	1	0	1	1	1	0	1	0	0	0	0
189	0	0	1	1	1	1	0	1	0	0	0	0
190	1	0	1	1	1	1	0	1	0	0	0	0
191	0	1	1	1	1	1	0	1	0	0	0	0
192	1	1	1	1	1	1	0	1	0	0	0	0
193	0	0	0	0	0	0	1	1	4	2	0	0
194	1	0	0	0	0	0	1	1	0	0	0	0
195	0	1	0	0	0	0	1	1	0	0	0	0
196	1	1	0	0	0	0	1	1	0	0	0	0
197	0	0	1	0	0	0	1	1	11	9	0	0
198	1	0	1	0	0	0	1	1	0	0	0	0
199	0	1	1	0	0	0	1	1	0	0	0	0
200	1	1	1	0	0	0	1	1	0	0	0	0
201	0	0	0	1	0	0	1	1	0	0	0	0
202	1	0	0	1	0	0	1	1	0	0	0	0
203	0	1	0	1	0	0	1	1	0	0	0	0
204	1	1	0	1	0	0	1	1	0	0	0	0
205	0	0	1	1	0	0	1	1	0	0	0	0
206	1	0	1	1	0	0	1	1	0	0	0	0
207	0	1	1	1	0	0	1	1	0	0	0	0
208	1	1	1	1	0	0	1	1	0	0	0	0
209	0	0	0	0	1	0	1	1	0	0	0	0
210	1	0	0	0	1	0	1	1	0	0	0	0
211	0	1	0	0	1	0	1	1	0	0	0	0
212	1	1	0	0	1	0	1	1	0	0	0	0
213	0	0	1	0	1	0	1	1	0	0	0	0
214	1	0	1	0	1	0	1	1	0	0	0	0
215	0	1	1	0	1	0	1	1	0	0	0	0
216	1	1	1	0	1	0	1	1	0	0	0	0
217	0	0	0	1	1	0	1	1	0	0	0	0
218	1	0	0	1	1	0	1	1	0	0	0	0
219	0	1	0	1	1	0	1	1	0	0	0	0
220	1	1	0	1	1	0	1	1	0	0	0	0
221	0	0	1	1	1	0	1	1	0	0	0	0
222	1	0	1	1	1	0	1	1	0	0	0	0

223	0	1	1	1	1	0	1	1	0	0	0	0
224	1	1	1	1	1	0	1	1	0	0	0	0
225	0	0	0	0	0	1	1	1	0	0	0	0
226	1	0	0	0	0	1	1	1	0	0	0	0
227	0	1	0	0	0	1	1	1	0	0	0	0
228	1	1	0	0	0	1	1	1	0	0	0	0
229	0	0	1	0	0	1	1	1	0	0	0	0
230	1	0	1	0	0	1	1	1	0	0	0	0
231	0	1	1	0	0	1	1	1	0	0	0	0
232	1	1	1	0	0	1	1	1	7	1	0	0
233	0	0	0	1	0	1	1	1	0	0	0	0
234	1	0	0	1	0	1	1	1	0	1	0	0
235	0	1	0	1	0	1	1	1	0	0	0	0
236	1	1	0	1	0	1	1	1	0	0	0	0
237	0	0	1	1	0	1	1	1	0	0	0	0
238	1	0	1	1	0	1	1	1	0	0	0	0
239	0	1	1	1	0	1	1	1	0	0	0	0
240	1	1	1	1	0	1	1	1	7	10	0	0
241	0	0	0	0	1	1	1	1	0	0	0	0
242	1	0	0	0	1	1	1	1	0	0	0	0
243	0	1	0	0	1	1	1	1	0	0	0	0
244	1	1	0	0	1	1	1	1	0	0	0	0
245	0	0	1	0	1	1	1	1	0	0	0	0
246	1	0	1	0	1	1	1	1	0	0	0	0
247	0	1	1	0	1	1	1	1	0	0	0	0
248	1	1	1	0	1	1	1	1	0	0	0	0
249	0	0	0	1	1	1	1	1	0	0	0	0
250	1	0	0	1	1	1	1	1	0	0	0	0
251	0	1	0	1	1	1	1	1	0	0	0	0
252	1	1	0	1	1	1	1	1	0	0	0	0
253	0	0	1	1	1	1	1	1	0	0	0	0
254	1	0	1	1	1	1	1	1	0	0	0	0
255	0	1	1	1	1	1	1	1	0	0	0	0
256	1	1	1	1	1	1	1	1	44	31	0	0

	pre1	pre2	Sp1	Sp2	Sp3	Sp4	Sp5	Sp6	Sp7	Sp8	Se1	Se2	Se3	Se4
Best Guess	0.2	0.2	0.95	0.95	0.95	0.95	0.95	0.95	0.95	0.95	0.8	0.8	0.8	0.8

	Se5	Se6	Se7	Se8
Best Guess	0.8	0.8	0.8	0.8

EXPECTATION MAXIMISATION

$Iterations

[1] 7

```
$LogLikelihood
[1] -857.8069

$Estimations
     pre1   pre2 Sp1   Sp2   Sp3 Sp4    Sp5   Sp6    Sp7    Sp8    Se1    Se2
Est 0.1864 0.2196   1 0.977 0.954   1 0.9977 0.977 0.9402 0.9173 0.9443 0.9629
      Se3    Se4    Se5    Se6    Se7    Se8
Est 0.9814 0.9165 0.7313 0.9907 0.9906 0.9352
```

NEWTON-RAPHSON

```
$Iterations
[1] 30

$LogLikelihood
[1] -857.8069

$Estimations
        pre1   pre2 Sp1    Sp2    Sp3 Sp4    Sp5    Sp6    Sp7    Sp8    Se1
Est   0.1864 0.2196   1 0.9770 0.9540   1 0.9977 0.9770 0.9402 0.9173 0.9443
CIinf 0.1484 0.1681 NaN 0.9578 0.9298 NaN 0.9839 0.9578 0.9137 0.8874 0.8814
CIsup 0.2316 0.2814 NaN 0.9876 0.9702   1 0.9997 0.9876 0.9590 0.9398 0.9748
         Se2    Se3    Se4    Se5    Se6    Se7    Se8
Est   0.9629 0.9814 0.9165 0.7313 0.9907 0.9906 0.9352
CIinf 0.9052 0.9285 0.8472 0.6401 0.9366 0.9364 0.8702
CIsup 0.9860 0.9953 0.9560 0.8064 0.9987 0.9987 0.9688
```

WARNING 1: test results are assumed to be independent conditional on infection or disease status
WARNING 2: tests are supposed to have constant sensitivity and specificity in all populations

Expected Results (NR) and Goodness-of-fit test

```
$Expected
  test1 test2 test3 test4 test5 test6 test7 test8 pop1 pop2 RefInd RefInf
1     0     0     0     0     0     0     0     0  254  144      0      0
2     1     0     0     0     0     0     0     0    0    0      0      0
3     0     1     0     0     0     0     0     0    0    0      0      0
4     1     1     0     0     0     0     0     0    0    0      0      0
5     0     0     1     0     0     0     0     0    0    0      0      0
6     1     0     1     0     0     0     0     0    0    0      0      0
7     0     1     1     0     0     0     0     0    0    0      0      0
8     1     1     1     0     0     0     0     0    0    0      0      0
9     0     0     0     1     0     0     0     0    0    0      0      0
```

10	1	0	0	1	0	0	0	0	0	0	0	0
11	0	1	0	1	0	0	0	0	0	0	0	0
12	1	1	0	1	0	0	0	0	0	0	0	0
13	0	0	1	1	0	0	0	0	0	0	0	0
14	1	0	1	1	0	0	0	0	0	0	0	0
15	0	1	1	1	0	0	0	0	0	0	0	0
16	1	1	1	1	0	0	0	0	0	0	0	0
17	0	0	0	0	1	0	0	0	0	1	0	0
18	1	0	0	0	1	0	0	0	0	0	0	0
19	0	1	0	0	1	0	0	0	0	0	0	0
20	1	1	0	0	1	0	0	0	0	0	0	0
21	0	0	1	0	1	0	0	0	0	0	0	0
22	1	0	1	0	1	0	0	0	0	0	0	0
23	0	1	1	0	1	0	0	0	0	0	0	0
24	1	1	1	0	1	0	0	0	1	0	0	0
25	0	0	0	1	1	0	0	0	0	0	0	0
26	1	0	0	1	1	0	0	0	0	0	0	0
27	0	1	0	1	1	0	0	0	0	0	0	0
28	1	1	0	1	1	0	0	0	0	0	0	0
29	0	0	1	1	1	0	0	0	0	0	0	0
30	1	0	1	1	1	0	0	0	0	0	0	0
31	0	1	1	1	1	0	0	0	0	0	0	0
32	1	1	1	1	1	0	0	0	0	0	0	0
33	0	0	0	0	0	1	0	0	0	0	0	0
34	1	0	0	0	0	1	0	0	0	0	0	0
35	0	1	0	0	0	1	0	0	0	0	0	0
36	1	1	0	0	0	1	0	0	0	0	0	0
37	0	0	1	0	0	1	0	0	0	0	0	0
38	1	0	1	0	0	1	0	0	0	0	0	0
39	0	1	1	0	0	1	0	0	0	0	0	0
40	1	1	1	0	0	1	0	0	0	0	0	0
41	0	0	0	1	0	1	0	0	0	0	0	0
42	1	0	0	1	0	1	0	0	0	0	0	0
43	0	1	0	1	0	1	0	0	0	0	0	0
44	1	1	0	1	0	1	0	0	0	0	0	0
45	0	0	1	1	0	1	0	0	0	0	0	0
46	1	0	1	1	0	1	0	0	0	0	0	0
47	0	1	1	1	0	1	0	0	0	0	0	0
48	1	1	1	1	0	1	0	0	0	0	0	0
49	0	0	0	0	1	1	0	0	0	0	0	0
50	1	0	0	0	1	1	0	0	0	0	0	0
51	0	1	0	0	1	1	0	0	0	0	0	0
52	1	1	0	0	1	1	0	0	0	0	0	0
53	0	0	1	0	1	1	0	0	0	0	0	0

54	1	0	1	0	1	1	0	0	0	0	0	0
55	0	1	1	0	1	1	0	0	0	0	0	0
56	1	1	1	0	1	1	0	0	0	0	0	0
57	0	0	0	1	1	1	0	0	0	0	0	0
58	1	0	0	1	1	1	0	0	0	0	0	0
59	0	1	0	1	1	1	0	0	0	0	0	0
60	1	1	0	1	1	1	0	0	0	0	0	0
61	0	0	1	1	1	1	0	0	0	0	0	0
62	1	0	1	1	1	1	0	0	0	0	0	0
63	0	1	1	1	1	1	0	0	0	0	0	0
64	1	1	1	1	1	1	0	0	0	0	0	0
65	0	0	0	0	0	0	1	0	0	0	0	0
66	1	0	0	0	0	0	1	0	0	0	0	0
67	0	1	0	0	0	0	1	0	0	0	0	0
68	1	1	0	0	0	0	1	0	0	0	0	0
69	0	0	1	0	0	0	1	0	0	0	0	0
70	1	0	1	0	0	0	1	0	0	0	0	0
71	0	1	1	0	0	0	1	0	0	0	0	0
72	1	1	1	0	0	0	1	0	0	0	0	0
73	0	0	0	1	0	0	1	0	0	0	0	0
74	1	0	0	1	0	0	1	0	0	0	0	0
75	0	1	0	1	0	0	1	0	0	0	0	0
76	1	1	0	1	0	0	1	0	0	0	0	0
77	0	0	1	1	0	0	1	0	0	0	0	0
78	1	0	1	1	0	0	1	0	0	0	0	0
79	0	1	1	1	0	0	1	0	0	0	0	0
80	1	1	1	1	0	0	1	0	0	0	0	0
81	0	0	0	0	1	0	1	0	0	0	0	0
82	1	0	0	0	1	0	1	0	0	0	0	0
83	0	1	0	0	1	0	1	0	0	0	0	0
84	1	1	0	0	1	0	1	0	0	0	0	0
85	0	0	1	0	1	0	1	0	0	0	0	0
86	1	0	1	0	1	0	1	0	0	0	0	0
87	0	1	1	0	1	0	1	0	0	0	0	0
88	1	1	1	0	1	0	1	0	0	0	0	0
89	0	0	0	1	1	0	1	0	0	0	0	0
90	1	0	0	1	1	0	1	0	0	0	0	0
91	0	1	0	1	1	0	1	0	0	0	0	0
92	1	1	0	1	1	0	1	0	0	0	0	0
93	0	0	1	1	1	0	1	0	0	0	0	0
94	1	0	1	1	1	0	1	0	0	0	0	0
95	0	1	1	1	1	0	1	0	0	0	0	0
96	1	1	1	1	1	0	1	0	0	0	0	0
97	0	0	0	0	0	1	1	0	0	0	0	0

98	1	0	0	0	0	1	1	0	0	0	0	0
99	0	1	0	0	0	1	1	0	0	0	0	0
100	1	1	0	0	0	1	1	0	0	0	0	0
101	0	0	1	0	0	1	1	0	0	0	0	0
102	1	0	1	0	0	1	1	0	0	0	0	0
103	0	1	1	0	0	1	1	0	0	0	0	0
104	1	1	1	0	0	1	1	0	0	0	0	0
105	0	0	0	1	0	1	1	0	0	0	0	0
106	1	0	0	1	0	1	1	0	0	0	0	0
107	0	1	0	1	0	1	1	0	0	0	0	0
108	1	1	0	1	0	1	1	0	0	0	0	0
109	0	0	1	1	0	1	1	0	0	2	0	0
110	1	0	1	1	0	1	1	0	0	0	0	0
111	0	1	1	1	0	1	1	0	1	0	0	0
112	1	1	1	1	0	1	1	0	0	0	0	0
113	0	0	0	0	1	1	1	0	0	0	0	0
114	1	0	0	0	1	1	1	0	0	0	0	0
115	0	1	0	0	1	1	1	0	0	0	0	0
116	1	1	0	0	1	1	1	0	0	0	0	0
117	0	0	1	0	1	1	1	0	0	0	0	0
118	1	0	1	0	1	1	1	0	0	0	0	0
119	0	1	1	0	1	1	1	0	0	0	0	0
120	1	1	1	0	1	1	1	0	0	0	0	0
121	0	0	0	1	1	1	1	0	1	0	0	0
122	1	0	0	1	1	1	1	0	0	0	0	0
123	0	1	0	1	1	1	1	0	0	0	0	0
124	1	1	0	1	1	1	1	0	0	0	0	0
125	0	0	1	1	1	1	1	0	0	0	0	0
126	1	0	1	1	1	1	1	0	0	0	0	0
127	0	1	1	1	1	1	1	0	2	0	0	0
128	1	1	1	1	1	1	1	0	0	0	0	0
129	0	0	0	0	0	0	0	1	0	0	0	0
130	1	0	0	0	0	0	0	1	0	0	0	0
131	0	1	0	0	0	0	0	1	0	0	0	0
132	1	1	0	0	0	0	0	1	0	0	0	0
133	0	0	1	0	0	0	0	1	0	0	0	0
134	1	0	1	0	0	0	0	1	0	0	0	0
135	0	1	1	0	0	0	0	1	0	0	0	0
136	1	1	1	0	0	0	0	1	0	0	0	0
137	0	0	0	1	0	0	0	1	0	0	0	0
138	1	0	0	1	0	0	0	1	0	0	0	0
139	0	1	0	1	0	0	0	1	0	0	0	0
140	1	1	0	1	0	0	0	1	0	0	0	0
141	0	0	1	1	0	0	0	1	0	0	0	0

142	1	0	1	1	0	0	0	1	0	0	0	0
143	0	1	1	1	0	0	0	1	0	0	0	0
144	1	1	1	1	0	0	0	1	0	0	0	0
145	0	0	0	0	1	0	0	1	0	0	0	0
146	1	0	0	0	1	0	0	1	0	0	0	0
147	0	1	0	0	1	0	0	1	0	0	0	0
148	1	1	0	0	1	0	0	1	0	0	0	0
149	0	0	1	0	1	0	0	1	0	0	0	0
150	1	0	1	0	1	0	0	1	0	0	0	0
151	0	1	1	0	1	0	0	1	0	0	0	0
152	1	1	1	0	1	0	0	1	0	0	0	0
153	0	0	0	1	1	0	0	1	0	0	0	0
154	1	0	0	1	1	0	0	1	0	0	0	0
155	0	1	0	1	1	0	0	1	0	0	0	0
156	1	1	0	1	1	0	0	1	0	0	0	0
157	0	0	1	1	1	0	0	1	0	0	0	0
158	1	0	1	1	1	0	0	1	0	0	0	0
159	0	1	1	1	1	0	0	1	0	0	0	0
160	1	1	1	1	1	0	0	1	0	0	0	0
161	0	0	0	0	0	1	0	1	0	0	0	0
162	1	0	0	0	0	1	0	1	0	0	0	0
163	0	1	0	0	0	1	0	1	6	4	0	0
164	1	1	0	0	0	1	0	1	0	0	0	0
165	0	0	1	0	0	1	0	1	0	0	0	0
166	1	0	1	0	0	1	0	1	0	0	0	0
167	0	1	1	0	0	1	0	1	0	0	0	0
168	1	1	1	0	0	1	0	1	0	0	0	0
169	0	0	0	1	0	1	0	1	0	0	0	0
170	1	0	0	1	0	1	0	1	0	0	0	0
171	0	1	0	1	0	1	0	1	0	0	0	0
172	1	1	0	1	0	1	0	1	0	0	0	0
173	0	0	1	1	0	1	0	1	0	0	0	0
174	1	0	1	1	0	1	0	1	0	0	0	0
175	0	1	1	1	0	1	0	1	0	0	0	0
176	1	1	1	1	0	1	0	1	0	0	0	0
177	0	0	0	0	1	1	0	1	0	0	0	0
178	1	0	0	0	1	1	0	1	0	0	0	0
179	0	1	0	0	1	1	0	1	0	0	0	0
180	1	1	0	0	1	1	0	1	0	0	0	0
181	0	0	1	0	1	1	0	1	0	0	0	0
182	1	0	1	0	1	1	0	1	0	0	0	0
183	0	1	1	0	1	1	0	1	0	0	0	0
184	1	1	1	0	1	1	0	1	0	0	0	0
185	0	0	0	1	1	1	0	1	0	0	0	0

186	1	0	0	1	1	1	0	1	0	0	0	0
187	0	1	0	1	1	1	0	1	0	0	0	0
188	1	1	0	1	1	1	0	1	0	0	0	0
189	0	0	1	1	1	1	0	1	0	0	0	0
190	1	0	1	1	1	1	0	1	0	0	0	0
191	0	1	1	1	1	1	0	1	0	0	0	0
192	1	1	1	1	1	1	0	1	0	0	0	0
193	0	0	0	0	0	0	1	1	4	2	0	0
194	1	0	0	0	0	0	1	1	0	0	0	0
195	0	1	0	0	0	0	1	1	0	0	0	0
196	1	1	0	0	0	0	1	1	0	0	0	0
197	0	0	1	0	0	0	1	1	11	9	0	0
198	1	0	1	0	0	0	1	1	0	0	0	0
199	0	1	1	0	0	0	1	1	0	0	0	0
200	1	1	1	0	0	0	1	1	0	0	0	0
201	0	0	0	1	0	0	1	1	0	0	0	0
202	1	0	0	1	0	0	1	1	0	0	0	0
203	0	1	0	1	0	0	1	1	0	0	0	0
204	1	1	0	1	0	0	1	1	0	0	0	0
205	0	0	1	1	0	0	1	1	0	0	0	0
206	1	0	1	1	0	0	1	1	0	0	0	0
207	0	1	1	1	0	0	1	1	0	0	0	0
208	1	1	1	1	0	0	1	1	0	0	0	0
209	0	0	0	0	1	0	1	1	0	0	0	0
210	1	0	0	0	1	0	1	1	0	0	0	0
211	0	1	0	0	1	0	1	1	0	0	0	0
212	1	1	0	0	1	0	1	1	0	0	0	0
213	0	0	1	0	1	0	1	1	0	0	0	0
214	1	0	1	0	1	0	1	1	0	0	0	0
215	0	1	1	0	1	0	1	1	0	0	0	0
216	1	1	1	0	1	0	1	1	0	0	0	0
217	0	0	0	1	1	0	1	1	0	0	0	0
218	1	0	0	1	1	0	1	1	0	0	0	0
219	0	1	0	1	1	0	1	1	0	0	0	0
220	1	1	0	1	1	0	1	1	0	0	0	0
221	0	0	1	1	1	0	1	1	0	0	0	0
222	1	0	1	1	1	0	1	1	0	0	0	0
223	0	1	1	1	1	0	1	1	0	0	0	0
224	1	1	1	1	1	0	1	1	0	0	0	0
225	0	0	0	0	0	1	1	1	0	0	0	0
226	1	0	0	0	0	1	1	1	0	0	0	0
227	0	1	0	0	0	1	1	1	0	0	0	0
228	1	1	0	0	0	1	1	1	0	0	0	0
229	0	0	1	0	0	1	1	1	0	0	0	0

230	1	0	1	0	0	1	1	1	0	0	0	0
231	0	1	1	0	0	1	1	1	0	0	0	0
232	1	1	1	0	0	1	1	1	7	1	0	0
233	0	0	0	1	0	1	1	1	0	0	0	0
234	1	0	0	1	0	1	1	1	0	1	0	0
235	0	1	0	1	0	1	1	1	0	0	0	0
236	1	1	0	1	0	1	1	1	0	0	0	0
237	0	0	1	1	0	1	1	1	0	0	0	0
238	1	0	1	1	0	1	1	1	0	0	0	0
239	0	1	1	1	0	1	1	1	0	0	0	0
240	1	1	1	1	0	1	1	1	7	10	0	0
241	0	0	0	0	1	1	1	1	0	0	0	0
242	1	0	0	0	1	1	1	1	0	0	0	0
243	0	1	0	0	1	1	1	1	0	0	0	0
244	1	1	0	0	1	1	1	1	0	0	0	0
245	0	0	1	0	1	1	1	1	0	0	0	0
246	1	0	1	0	1	1	1	1	0	0	0	0
247	0	1	1	0	1	1	1	1	0	0	0	0
248	1	1	1	0	1	1	1	1	0	0	0	0
249	0	0	0	1	1	1	1	1	0	0	0	0
250	1	0	0	1	1	1	1	1	0	0	0	0
251	0	1	0	1	1	1	1	1	0	0	0	0
252	1	1	0	1	1	1	1	1	0	0	0	0
253	0	0	1	1	1	1	1	1	0	0	0	0
254	1	0	1	1	1	1	1	1	0	0	0	0
255	0	1	1	1	1	1	1	1	0	0	0	0
256	1	1	1	1	1	1	1	1	44	31	0	0

	ExpPop1	ExpPop2	ExpRefInd	ExpRefInf
1	215.50	125.38	0	0
2	0.00	0.00	0	0
3	5.06	2.95	0	0
4	0.00	0.00	0	0
5	10.38	6.04	0	0
6	0.00	0.00	0	0
7	0.24	0.14	0	0
8	0.00	0.00	0	0
9	0.00	0.00	0	0
10	0.00	0.00	0	0
11	0.00	0.00	0	0
12	0.00	0.00	0	0
13	0.00	0.00	0	0
14	0.00	0.00	0	0
15	0.00	0.00	0	0
16	0.00	0.00	0	0

17	0.50	0.29	0	0
18	0.00	0.00	0	0
19	0.01	0.01	0	0
20	0.00	0.00	0	0
21	0.02	0.01	0	0
22	0.00	0.00	0	0
23	0.00	0.00	0	0
24	0.00	0.00	0	0
25	0.00	0.00	0	0
26	0.00	0.00	0	0
27	0.00	0.00	0	0
28	0.00	0.00	0	0
29	0.00	0.00	0	0
30	0.00	0.00	0	0
31	0.00	0.00	0	0
32	0.00	0.00	0	0
33	5.06	2.95	0	0
34	0.00	0.00	0	0
35	0.12	0.07	0	0
36	0.00	0.00	0	0
37	0.24	0.14	0	0
38	0.00	0.00	0	0
39	0.01	0.00	0	0
40	0.00	0.00	0	0
41	0.00	0.00	0	0
42	0.00	0.00	0	0
43	0.00	0.00	0	0
44	0.00	0.00	0	0
45	0.00	0.00	0	0
46	0.00	0.00	0	0
47	0.00	0.00	0	0
48	0.01	0.01	0	0
49	0.01	0.01	0	0
50	0.00	0.00	0	0
51	0.00	0.00	0	0
52	0.00	0.00	0	0
53	0.00	0.00	0	0
54	0.00	0.00	0	0
55	0.00	0.00	0	0
56	0.00	0.00	0	0
57	0.00	0.00	0	0
58	0.00	0.00	0	0
59	0.00	0.00	0	0
60	0.00	0.00	0	0

61	0.00	0.00	0	0
62	0.00	0.00	0	0
63	0.00	0.00	0	0
64	0.02	0.02	0	0
65	13.69	7.97	0	0
66	0.00	0.00	0	0
67	0.32	0.19	0	0
68	0.00	0.00	0	0
69	0.66	0.38	0	0
70	0.00	0.00	0	0
71	0.02	0.01	0	0
72	0.00	0.00	0	0
73	0.00	0.00	0	0
74	0.00	0.00	0	0
75	0.00	0.00	0	0
76	0.00	0.00	0	0
77	0.00	0.00	0	0
78	0.00	0.00	0	0
79	0.00	0.00	0	0
80	0.01	0.01	0	0
81	0.03	0.02	0	0
82	0.00	0.00	0	0
83	0.00	0.00	0	0
84	0.00	0.00	0	0
85	0.00	0.00	0	0
86	0.00	0.00	0	0
87	0.00	0.00	0	0
88	0.00	0.00	0	0
89	0.00	0.00	0	0
90	0.00	0.00	0	0
91	0.00	0.00	0	0
92	0.00	0.00	0	0
93	0.00	0.00	0	0
94	0.00	0.00	0	0
95	0.00	0.00	0	0
96	0.02	0.02	0	0
97	0.32	0.19	0	0
98	0.00	0.00	0	0
99	0.01	0.00	0	0
100	0.00	0.00	0	0
101	0.02	0.01	0	0
102	0.00	0.00	0	0
103	0.01	0.00	0	0
104	0.08	0.06	0	0

105	0.00	0.00	0	0
106	0.00	0.00	0	0
107	0.00	0.00	0	0
108	0.02	0.01	0	0
109	0.00	0.00	0	0
110	0.03	0.02	0	0
111	0.05	0.04	0	0
112	0.88	0.63	0	0
113	0.00	0.00	0	0
114	0.00	0.00	0	0
115	0.00	0.00	0	0
116	0.00	0.00	0	0
117	0.00	0.00	0	0
118	0.01	0.01	0	0
119	0.01	0.01	0	0
120	0.22	0.16	0	0
121	0.00	0.00	0	0
122	0.00	0.00	0	0
123	0.00	0.00	0	0
124	0.05	0.03	0	0
125	0.01	0.00	0	0
126	0.09	0.07	0	0
127	0.14	0.10	0	0
128	2.40	1.71	0	0
129	19.43	11.31	0	0
130	0.00	0.00	0	0
131	0.46	0.27	0	0
132	0.00	0.00	0	0
133	0.94	0.54	0	0
134	0.00	0.00	0	0
135	0.02	0.01	0	0
136	0.00	0.00	0	0
137	0.00	0.00	0	0
138	0.00	0.00	0	0
139	0.00	0.00	0	0
140	0.00	0.00	0	0
141	0.00	0.00	0	0
142	0.00	0.00	0	0
143	0.00	0.00	0	0
144	0.00	0.00	0	0
145	0.04	0.03	0	0
146	0.00	0.00	0	0
147	0.00	0.00	0	0
148	0.00	0.00	0	0

149	0.00	0.00	0	0
150	0.00	0.00	0	0
151	0.00	0.00	0	0
152	0.00	0.00	0	0
153	0.00	0.00	0	0
154	0.00	0.00	0	0
155	0.00	0.00	0	0
156	0.00	0.00	0	0
157	0.00	0.00	0	0
158	0.00	0.00	0	0
159	0.00	0.00	0	0
160	0.00	0.00	0	0
161	0.46	0.27	0	0
162	0.00	0.00	0	0
163	0.01	0.01	0	0
164	0.00	0.00	0	0
165	0.02	0.01	0	0
166	0.00	0.00	0	0
167	0.00	0.00	0	0
168	0.01	0.01	0	0
169	0.00	0.00	0	0
170	0.00	0.00	0	0
171	0.00	0.00	0	0
172	0.00	0.00	0	0
173	0.00	0.00	0	0
174	0.00	0.00	0	0
175	0.01	0.01	0	0
176	0.12	0.09	0	0
177	0.00	0.00	0	0
178	0.00	0.00	0	0
179	0.00	0.00	0	0
180	0.00	0.00	0	0
181	0.00	0.00	0	0
182	0.00	0.00	0	0
183	0.00	0.00	0	0
184	0.03	0.02	0	0
185	0.00	0.00	0	0
186	0.00	0.00	0	0
187	0.00	0.00	0	0
188	0.01	0.00	0	0
189	0.00	0.00	0	0
190	0.01	0.01	0	0
191	0.02	0.01	0	0
192	0.33	0.23	0	0

193	1.23	0.72	0	0
194	0.00	0.00	0	0
195	0.03	0.02	0	0
196	0.00	0.00	0	0
197	0.06	0.03	0	0
198	0.00	0.00	0	0
199	0.00	0.00	0	0
200	0.01	0.01	0	0
201	0.00	0.00	0	0
202	0.00	0.00	0	0
203	0.00	0.00	0	0
204	0.00	0.00	0	0
205	0.00	0.00	0	0
206	0.00	0.00	0	0
207	0.01	0.01	0	0
208	0.12	0.09	0	0
209	0.00	0.00	0	0
210	0.00	0.00	0	0
211	0.00	0.00	0	0
212	0.00	0.00	0	0
213	0.00	0.00	0	0
214	0.00	0.00	0	0
215	0.00	0.00	0	0
216	0.03	0.02	0	0
217	0.00	0.00	0	0
218	0.00	0.00	0	0
219	0.00	0.00	0	0
220	0.01	0.00	0	0
221	0.00	0.00	0	0
222	0.01	0.01	0	0
223	0.02	0.01	0	0
224	0.33	0.23	0	0
225	0.03	0.02	0	0
226	0.00	0.00	0	0
227	0.00	0.00	0	0
228	0.02	0.02	0	0
229	0.00	0.00	0	0
230	0.04	0.03	0	0
231	0.07	0.05	0	0
232	1.16	0.83	0	0
233	0.00	0.00	0	0
234	0.01	0.01	0	0
235	0.01	0.01	0	0
236	0.24	0.17	0	0

```
237   0.03   0.02     0     0
238   0.49   0.35     0     0
239   0.75   0.54     0     0
240  12.71   9.08     0     0
241   0.00   0.00     0     0
242   0.00   0.00     0     0
243   0.00   0.00     0     0
244   0.06   0.04     0     0
245   0.01   0.01     0     0
246   0.12   0.09     0     0
247   0.19   0.13     0     0
248   3.15   2.25     0     0
249   0.00   0.00     0     0
250   0.03   0.02     0     0
251   0.04   0.03     0     0
252   0.66   0.47     0     0
253   0.08   0.06     0     0
254   1.33   0.95     0     0
255   2.04   1.46     0     0
256  34.59  24.71     0     0

$Test
 Max LogLikelihood: Achievable  Obtained Deviance d.f.      p value
                -541.8923 -857.8069 631.8292  492 1.938381e-05

$Commentary
[1] "The model does not fit: Assumptions may be not justified"


Residual  correlations between test
$ResCor
         Corr1-2     Corr1-3     Corr1-4    Corr1-5     Corr1-6
pop 1 : 0.02966545  0.02748822 -0.04146374 0.02335436 -0.007204700
pop 2 : 0.03676788 -0.01556004  0.04393568 0.01275859  0.008905438
           Corr1-7    Corr1-8     Corr2-3     Corr2-4    Corr2-5    Corr2-6
pop 1 :  0.0003667263 0.06535817  0.04146528 -0.01234181 0.03409523 0.12179659
pop 2 : -0.0023577244 0.05369714 -0.02207883  0.01071037 0.01003414 0.08589943
          Corr2-7   Corr2-8     Corr3-4     Corr3-5     Corr3-6   Corr3-7
pop 1 :  0.01363943 0.1267421 -0.01302279  0.03147218  0.007666826 0.1957482
pop 2 : -0.03651559 0.1205577  0.01348754 -0.03281901 -0.024305582 0.1829973
        Corr3-8    Corr4-5     Corr4-6     Corr4-7     Corr4-8     Corr5-6
pop 1 : 0.2007084 0.11974713 -0.009463266 -0.001237415 -0.01300391  0.01924680
pop 2 : 0.1809753 0.01560615  0.039334803  0.025655508  0.02724110 -0.03674752
          Corr5-7     Corr5-8     Corr6-7   Corr6-8   Corr7-8
```

```
pop 1 :  0.02362124 0.018492288  0.01576406 0.1127440 0.2519878
pop 2 : -0.04385803 0.004320285 -0.01371877 0.0908689 0.2325526

$Commentary
[1] "The residuals should be randomly distributed around 0"


 BOOTSTRAP CONFIDENCE INTERVALS : 5000  samples
       pre1   pre2 Sp1    Sp2    Sp3 Sp4   Sp5    Sp6    Sp7    Sp8    Se1
CIinf 0.1456 0.1612   1 0.9618 0.9329   1 0.993 0.9618 0.9169 0.8894 0.8969
CIsup 0.2278 0.2781   1 0.9907 0.9726   1 1.000 0.9907 0.9614 0.9424 0.9825
        Se2    Se3    Se4    Se5    Se6    Se7    Se8
CIinf 0.9244 0.9524 0.8600 0.6442 0.9699 0.9696 0.8859
CIsup 0.9915 1.0000 0.9658 0.8130 1.0000 1.0000 0.9798
your data are stored in "new_8_double"
Note that you'll have to Save workspace image before leaving R if you want to use it in a new R
session
>
```
