# Supplementary material for: Evaluation of Simpler Criteria for Diagnosing Allergic Bronchopulmonary Aspergillosis Complicating Asthma
Source: Front Cell Infect Microbiol. 2022 Mar 25;12:861866. doi: 10.3389/fcimb.2022.861866 (PMC8990730; doi:10.3389/fcimb.2022.861866)
Supplement: Supplementary file 2 [file Table_2.pdf]

4.1.2 (2021-11-01) -- "Bird Hippie"
Copyright (C) 2021 The R Foundation for Statistical Computing
Platform: x86_64-w64-mingw32/x64 (64-bit)

R is free software and comes with ABSOLUTELY NO WARRANTY.
You are welcome to redistribute it under certain conditions.
Type 'license()' or 'licence()' for distribution details.

  Natural language support but running in an English locale

R is a collaborative project with many contributors.
Type 'contributors()' for more information and
'citation()' on how to cite R or R packages in publications.

Type 'demo()' for some demos, 'help()' for on-line help, or
'help.start()' for an HTML browser interface to help.
Type 'q()' to quit R.

[Previously saved workspace restored]

> source("D:\\Dropbox\\Documents\\TAGS.R")
> TAGS()

TAGS V.2.0 is a R program developed by
R. Pouillot and G. Gerbier, Agence Française de Sécurité Sanitaire des Aliments, France.
r.pouillot@afssa.fr

Its purpose is to evaluate diagnostic tests in the absence of a gold standard,
using Maximum Likelihood Estimation (Newton Raphson and Expectation Maximisation algorithms).

For further details: see
Pouillot R., Gerbier G. (2001)
'Tags' a program for validation of the diagnostic values of tests in the absence of a gold standard.
Proceedings of the Society for Veterinary Epidemiology and Preventive Medecine, Noordwijkerhout, The netherlands, 28th - 30th March 2001: 37-48

Reference(s) population(s) data may be used (population(s) with a known infection status)

Evaluation may be used as soon as df>=parameters
A goodness-of-fit test and residual correlations are then provided

Three sets of data may be used as examples :

Hui and Walter (Biometrics, 1980, 36:167-171): Enter hui
Saegerman et al (Vet Record, 1999, 145:214-8): Enter sae
Handelman's dentistry data (JADA, 1986, 113:751-754): Enter qu
If you want to enter new data: Enter new
If you want to use loaded data: Enter the name the program has already given to you

data Set ? new
Enter a name for your data : new_8_single

ENTER YOUR data
Number of tests (between 1 and 10) ? 8
Number of population without unknown status (between 1 and 10) ? 1

Do you have Reference Population(s) ?
No : Enter 0
Yes, Disease free : Enter 1
Yes, Infected : Enter 2
Yes, one Disease-free and one Infected : Enter 3
0

population with unknown status 1
Number of results 0 0 0 0 0 0 0 0 in population with unknown status 1 : 398
Number of results 1 0 0 0 0 0 0 0 in population with unknown status 1 : 0
Number of results 0 1 0 0 0 0 0 0 in population with unknown status 1 : 0
Number of results 1 1 0 0 0 0 0 0 in population with unknown status 1 : 0
Number of results 0 0 1 0 0 0 0 0 in population with unknown status 1 : 0
Number of results 1 0 1 0 0 0 0 0 in population with unknown status 1 : 0
Number of results 0 1 1 0 0 0 0 0 in population with unknown status 1 : 0
Number of results 1 1 1 0 0 0 0 0 in population with unknown status 1 : 0
Number of results 0 0 0 1 0 0 0 0 in population with unknown status 1 : 0
Number of results 1 0 0 1 0 0 0 0 in population with unknown status 1 : 0
Number of results 0 1 0 1 0 0 0 0 in population with unknown status 1 : 0
Number of results 1 1 0 1 0 0 0 0 in population with unknown status 1 : 0
Number of results 0 0 1 1 0 0 0 0 in population with unknown status 1 : 0
Number of results 1 0 1 1 0 0 0 0 in population with unknown status 1 : 0
Number of results 0 1 1 1 0 0 0 0 in population with unknown status 1 : 0
Number of results 1 1 1 1 0 0 0 0 in population with unknown status 1 : 0
Number of results 0 0 0 0 1 0 0 0 in population with unknown status 1 : 1
Number of results 1 0 0 0 1 0 0 0 in population with unknown status 1 : 0
Number of results 0 1 0 0 1 0 0 0 in population with unknown status 1 : 0
Number of results 1 1 0 0 1 0 0 0 in population with unknown status 1 : 0
Number of results 0 0 1 0 1 0 0 0 in population with unknown status 1 : 0
Number of results 1 0 1 0 1 0 0 0 in population with unknown status 1 : 0

Number of results 0 1 1 0 1 0 0 0 in population with unknown status 1 : 0
Number of results 1 1 1 0 1 0 0 0 in population with unknown status 1 : 1
Number of results 0 0 0 1 1 0 0 0 in population with unknown status 1 : 0
Number of results 1 0 0 1 1 0 0 0 in population with unknown status 1 : 0
Number of results 0 1 0 1 1 0 0 0 in population with unknown status 1 : 0
Number of results 1 1 0 1 1 0 0 0 in population with unknown status 1 : 0
Number of results 0 0 1 1 1 0 0 0 in population with unknown status 1 : 0
Number of results 1 0 1 1 1 0 0 0 in population with unknown status 1 : 0
Number of results 0 1 1 1 1 0 0 0 in population with unknown status 1 : 0
Number of results 1 1 1 1 1 0 0 0 in population with unknown status 1 : 0
Number of results 0 0 0 0 0 1 0 0 in population with unknown status 1 : 0
Number of results 1 0 0 0 0 1 0 0 in population with unknown status 1 : 0
Number of results 0 1 0 0 0 1 0 0 in population with unknown status 1 : 0
Number of results 1 1 0 0 0 1 0 0 in population with unknown status 1 : 0
Number of results 0 0 1 0 0 1 0 0 in population with unknown status 1 : 0
Number of results 1 0 1 0 0 1 0 0 in population with unknown status 1 : 0
Number of results 0 1 1 0 0 1 0 0 in population with unknown status 1 : 0
Number of results 1 1 1 0 0 1 0 0 in population with unknown status 1 : 0
Number of results 0 0 0 1 0 1 0 0 in population with unknown status 1 : 0
Number of results 1 0 0 1 0 1 0 0 in population with unknown status 1 : 0
Number of results 0 1 0 1 0 1 0 0 in population with unknown status 1 : 0
Number of results 1 1 0 1 0 1 0 0 in population with unknown status 1 : 0
Number of results 0 0 1 1 0 1 0 0 in population with unknown status 1 : 0
Number of results 1 0 1 1 0 1 0 0 in population with unknown status 1 : 0
Number of results 0 1 1 1 0 1 0 0 in population with unknown status 1 : 0
Number of results 1 1 1 1 0 1 0 0 in population with unknown status 1 : 0
Number of results 0 0 0 0 1 1 0 0 in population with unknown status 1 : 0
Number of results 1 0 0 0 1 1 0 0 in population with unknown status 1 : 0
Number of results 0 1 0 0 1 1 0 0 in population with unknown status 1 : 0
Number of results 1 1 0 0 1 1 0 0 in population with unknown status 1 : 0
Number of results 0 0 1 0 1 1 0 0 in population with unknown status 1 : 0
Number of results 1 0 1 0 1 1 0 0 in population with unknown status 1 : 0
Number of results 0 1 1 0 1 1 0 0 in population with unknown status 1 : 0
Number of results 1 1 1 0 1 1 0 0 in population with unknown status 1 : 0
Number of results 0 0 0 1 1 1 0 0 in population with unknown status 1 : 0
Number of results 1 0 0 1 1 1 0 0 in population with unknown status 1 : 0
Number of results 0 1 0 1 1 1 0 0 in population with unknown status 1 : 0
Number of results 1 1 0 1 1 1 0 0 in population with unknown status 1 : 0
Number of results 0 0 1 1 1 1 0 0 in population with unknown status 1 : 0
Number of results 1 0 1 1 1 1 0 0 in population with unknown status 1 : 0
Number of results 0 1 1 1 1 1 0 0 in population with unknown status 1 : 0
Number of results 1 1 1 1 1 1 0 0 in population with unknown status 1 : 0
Number of results 0 0 0 0 0 0 1 0 in population with unknown status 1 : 0
Number of results 1 0 0 0 0 0 1 0 in population with unknown status 1 : 0

Number of results 0 1 0 0 0 0 1 0 in population with unknown status 1 : 0
Number of results 1 1 0 0 0 0 1 0 in population with unknown status 1 : 0
Number of results 0 0 1 0 0 0 1 0 in population with unknown status 1 : 0
Number of results 1 0 1 0 0 0 1 0 in population with unknown status 1 : 0
Number of results 0 1 1 0 0 0 1 0 in population with unknown status 1 : 0
Number of results 1 1 1 0 0 0 1 0 in population with unknown status 1 : 0
Number of results 0 0 0 1 0 0 1 0 in population with unknown status 1 : 0
Number of results 1 0 0 1 0 0 1 0 in population with unknown status 1 : 0
Number of results 0 1 0 1 0 0 1 0 in population with unknown status 1 : 0
Number of results 1 1 0 1 0 0 1 0 in population with unknown status 1 : 0
Number of results 0 0 1 1 0 0 1 0 in population with unknown status 1 : 0
Number of results 1 0 1 1 0 0 1 0 in population with unknown status 1 : 0
Number of results 0 1 1 1 0 0 1 0 in population with unknown status 1 : 0
Number of results 1 1 1 1 0 0 1 0 in population with unknown status 1 : 0
Number of results 0 0 0 0 1 0 1 0 in population with unknown status 1 : 0
Number of results 1 0 0 0 1 0 1 0 in population with unknown status 1 : 0
Number of results 0 1 0 0 1 0 1 0 in population with unknown status 1 : 0
Number of results 1 1 0 0 1 0 1 0 in population with unknown status 1 : 0
Number of results 0 0 1 0 1 0 1 0 in population with unknown status 1 : 0
Number of results 1 0 1 0 1 0 1 0 in population with unknown status 1 : 0
Number of results 0 1 1 0 1 0 1 0 in population with unknown status 1 : 0
Number of results 1 1 1 0 1 0 1 0 in population with unknown status 1 : 0
Number of results 0 0 0 1 1 0 1 0 in population with unknown status 1 : 0
Number of results 1 0 0 1 1 0 1 0 in population with unknown status 1 : 0
Number of results 0 1 0 1 1 0 1 0 in population with unknown status 1 : 0
Number of results 1 1 0 1 1 0 1 0 in population with unknown status 1 : 0
Number of results 0 0 1 1 1 0 1 0 in population with unknown status 1 : 0
Number of results 1 0 1 1 1 0 1 0 in population with unknown status 1 : 0
Number of results 0 1 1 1 1 0 1 0 in population with unknown status 1 : 0
Number of results 1 1 1 1 1 0 1 0 in population with unknown status 1 : 0
Number of results 0 0 0 0 0 1 1 0 in population with unknown status 1 : 0
Number of results 1 0 0 0 0 1 1 0 in population with unknown status 1 : 0
Number of results 0 1 0 0 0 1 1 0 in population with unknown status 1 : 0
Number of results 1 1 0 0 0 1 1 0 in population with unknown status 1 : 0
Number of results 0 0 1 0 0 1 1 0 in population with unknown status 1 : 0
Number of results 1 0 1 0 0 1 1 0 in population with unknown status 1 : 0
Number of results 0 1 1 0 0 1 1 0 in population with unknown status 1 : 0
Number of results 1 1 1 0 0 1 1 0 in population with unknown status 1 : 0
Number of results 0 0 0 1 0 1 1 0 in population with unknown status 1 : 0
Number of results 1 0 0 1 0 1 1 0 in population with unknown status 1 : 0
Number of results 0 1 0 1 0 1 1 0 in population with unknown status 1 : 0
Number of results 1 1 0 1 0 1 1 0 in population with unknown status 1 : 0
Number of results 0 0 1 1 0 1 1 0 in population with unknown status 1 : 3
Number of results 1 0 1 1 0 1 1 0 in population with unknown status 1 : 0

Number of results 0 1 1 1 0 1 1 0 in population with unknown status 1 : 0
Number of results 1 1 1 1 0 1 1 0 in population with unknown status 1 : 0
Number of results 0 0 0 0 1 1 1 0 in population with unknown status 1 : 0
Number of results 1 0 0 0 1 1 1 0 in population with unknown status 1 : 0
Number of results 0 1 0 0 1 1 1 0 in population with unknown status 1 : 0
Number of results 1 1 0 0 1 1 1 0 in population with unknown status 1 : 0
Number of results 0 0 1 0 1 1 1 0 in population with unknown status 1 : 0
Number of results 1 0 1 0 1 1 1 0 in population with unknown status 1 : 0
Number of results 0 1 1 0 1 1 1 0 in population with unknown status 1 : 0
Number of results 1 1 1 0 1 1 1 0 in population with unknown status 1 : 0
Number of results 0 0 0 1 1 1 1 0 in population with unknown status 1 : 1
Number of results 1 0 0 1 1 1 1 0 in population with unknown status 1 : 0
Number of results 0 1 0 1 1 1 1 0 in population with unknown status 1 : 0
Number of results 1 1 0 1 1 1 1 0 in population with unknown status 1 : 0
Number of results 0 0 1 1 1 1 1 0 in population with unknown status 1 : 2
Number of results 1 0 1 1 1 1 1 0 in population with unknown status 1 : 0
Number of results 0 1 1 1 1 1 1 0 in population with unknown status 1 : 0
Number of results 1 1 1 1 1 1 1 0 in population with unknown status 1 : 0
Number of results 0 0 0 0 0 0 0 1 in population with unknown status 1 : 0
Number of results 1 0 0 0 0 0 0 1 in population with unknown status 1 : 0
Number of results 0 1 0 0 0 0 0 1 in population with unknown status 1 : 0
Number of results 1 1 0 0 0 0 0 1 in population with unknown status 1 : 0
Number of results 0 0 1 0 0 0 0 1 in population with unknown status 1 : 0
Number of results 1 0 1 0 0 0 0 1 in population with unknown status 1 : 0
Number of results 0 1 1 0 0 0 0 1 in population with unknown status 1 : 0
Number of results 1 1 1 0 0 0 0 1 in population with unknown status 1 : 0
Number of results 0 0 0 1 0 0 0 1 in population with unknown status 1 : 0
Number of results 1 0 0 1 0 0 0 1 in population with unknown status 1 : 0
Number of results 0 1 0 1 0 0 0 1 in population with unknown status 1 : 0
Number of results 1 1 0 1 0 0 0 1 in population with unknown status 1 : 0
Number of results 0 0 1 1 0 0 0 1 in population with unknown status 1 : 0
Number of results 1 0 1 1 0 0 0 1 in population with unknown status 1 : 0
Number of results 0 1 1 1 0 0 0 1 in population with unknown status 1 : 0
Number of results 1 1 1 1 0 0 0 1 in population with unknown status 1 : 0
Number of results 0 0 0 0 1 0 0 1 in population with unknown status 1 : 0
Number of results 1 0 0 0 1 0 0 1 in population with unknown status 1 : 0
Number of results 0 1 0 0 1 0 0 1 in population with unknown status 1 : 0
Number of results 1 1 0 0 1 0 0 1 in population with unknown status 1 : 0
Number of results 0 0 1 0 1 0 0 1 in population with unknown status 1 : 0
Number of results 1 0 1 0 1 0 0 1 in population with unknown status 1 : 0
Number of results 0 1 1 0 1 0 0 1 in population with unknown status 1 : 0
Number of results 1 1 1 0 1 0 0 1 in population with unknown status 1 : 0
Number of results 0 0 0 1 1 0 0 1 in population with unknown status 1 : 0
Number of results 1 0 0 1 1 0 0 1 in population with unknown status 1 : 0

Number of results 0 1 0 1 1 0 0 1 in population with unknown status 1 : 0
Number of results 1 1 0 1 1 0 0 1 in population with unknown status 1 : 0
Number of results 0 0 1 1 1 0 0 1 in population with unknown status 1 : 0
Number of results 1 0 1 1 1 0 0 1 in population with unknown status 1 : 0
Number of results 0 1 1 1 1 0 0 1 in population with unknown status 1 : 0
Number of results 1 1 1 1 1 0 0 1 in population with unknown status 1 : 0
Number of results 0 0 0 0 0 1 0 1 in population with unknown status 1 : 0
Number of results 1 0 0 0 0 1 0 1 in population with unknown status 1 : 0
Number of results 0 1 0 0 0 1 0 1 in population with unknown status 1 : 10
Number of results 1 1 0 0 0 1 0 1 in population with unknown status 1 : 0
Number of results 0 0 1 0 0 1 0 1 in population with unknown status 1 : 0
Number of results 1 0 1 0 0 1 0 1 in population with unknown status 1 : 0
Number of results 0 1 1 0 0 1 0 1 in population with unknown status 1 : 0
Number of results 1 1 1 0 0 1 0 1 in population with unknown status 1 : 0
Number of results 0 0 0 1 0 1 0 1 in population with unknown status 1 : 0
Number of results 1 0 0 1 0 1 0 1 in population with unknown status 1 : 0
Number of results 0 1 0 1 0 1 0 1 in population with unknown status 1 : 0
Number of results 1 1 0 1 0 1 0 1 in population with unknown status 1 : 0
Number of results 0 0 1 1 0 1 0 1 in population with unknown status 1 : 0
Number of results 1 0 1 1 0 1 0 1 in population with unknown status 1 : 0
Number of results 0 1 1 1 0 1 0 1 in population with unknown status 1 : 0
Number of results 1 1 1 1 0 1 0 1 in population with unknown status 1 : 0
Number of results 0 0 0 0 1 1 0 1 in population with unknown status 1 : 0
Number of results 1 0 0 0 1 1 0 1 in population with unknown status 1 : 0
Number of results 0 1 0 0 1 1 0 1 in population with unknown status 1 : 0
Number of results 1 1 0 0 1 1 0 1 in population with unknown status 1 : 0
Number of results 0 0 1 0 1 1 0 1 in population with unknown status 1 : 0
Number of results 1 0 1 0 1 1 0 1 in population with unknown status 1 : 0
Number of results 0 1 1 0 1 1 0 1 in population with unknown status 1 : 0
Number of results 1 1 1 0 1 1 0 1 in population with unknown status 1 : 0
Number of results 0 0 0 1 1 1 0 1 in population with unknown status 1 : 0
Number of results 1 0 0 1 1 1 0 1 in population with unknown status 1 : 0
Number of results 0 1 0 1 1 1 0 1 in population with unknown status 1 : 0
Number of results 1 1 0 1 1 1 0 1 in population with unknown status 1 : 0
Number of results 0 0 1 1 1 1 0 1 in population with unknown status 1 : 0
Number of results 1 0 1 1 1 1 0 1 in population with unknown status 1 : 0
Number of results 0 1 1 1 1 1 0 1 in population with unknown status 1 : 0
Number of results 1 1 1 1 1 1 0 1 in population with unknown status 1 : 0
Number of results 0 0 0 0 0 0 1 1 in population with unknown status 1 : 6
Number of results 1 0 0 0 0 0 1 1 in population with unknown status 1 : 0
Number of results 0 1 0 0 0 0 1 1 in population with unknown status 1 : 0
Number of results 1 1 0 0 0 0 1 1 in population with unknown status 1 : 0
Number of results 0 0 1 0 0 0 1 1 in population with unknown status 1 : 20
Number of results 1 0 1 0 0 0 1 1 in population with unknown status 1 : 0

Number of results 0 1 1 0 0 0 1 1 in population with unknown status 1 : 0
Number of results 1 1 1 0 0 0 1 1 in population with unknown status 1 : 0
Number of results 0 0 0 1 0 0 1 1 in population with unknown status 1 : 0
Number of results 1 0 0 1 0 0 1 1 in population with unknown status 1 : 0
Number of results 0 1 0 1 0 0 1 1 in population with unknown status 1 : 0
Number of results 1 1 0 1 0 0 1 1 in population with unknown status 1 : 0
Number of results 0 0 1 1 0 0 1 1 in population with unknown status 1 : 0
Number of results 1 0 1 1 0 0 1 1 in population with unknown status 1 : 0
Number of results 0 1 1 1 0 0 1 1 in population with unknown status 1 : 0
Number of results 1 1 1 1 0 0 1 1 in population with unknown status 1 : 0
Number of results 0 0 0 0 1 0 1 1 in population with unknown status 1 : 0
Number of results 1 0 0 0 1 0 1 1 in population with unknown status 1 : 0
Number of results 0 1 0 0 1 0 1 1 in population with unknown status 1 : 0
Number of results 1 1 0 0 1 0 1 1 in population with unknown status 1 : 0
Number of results 0 0 1 0 1 0 1 1 in population with unknown status 1 : 0
Number of results 1 0 1 0 1 0 1 1 in population with unknown status 1 : 0
Number of results 0 1 1 0 1 0 1 1 in population with unknown status 1 : 0
Number of results 1 1 1 0 1 0 1 1 in population with unknown status 1 : 0
Number of results 0 0 0 1 1 0 1 1 in population with unknown status 1 : 0
Number of results 1 0 0 1 1 0 1 1 in population with unknown status 1 : 0
Number of results 0 1 0 1 1 0 1 1 in population with unknown status 1 : 0
Number of results 1 1 0 1 1 0 1 1 in population with unknown status 1 : 0
Number of results 0 0 1 1 1 0 1 1 in population with unknown status 1 : 0
Number of results 1 0 1 1 1 0 1 1 in population with unknown status 1 : 0
Number of results 0 1 1 1 1 0 1 1 in population with unknown status 1 : 0
Number of results 1 1 1 1 1 0 1 1 in population with unknown status 1 : 0
Number of results 0 0 0 0 0 1 1 1 in population with unknown status 1 : 0
Number of results 1 0 0 0 0 1 1 1 in population with unknown status 1 : 0
Number of results 0 1 0 0 0 1 1 1 in population with unknown status 1 : 0
Number of results 1 1 0 0 0 1 1 1 in population with unknown status 1 : 0
Number of results 0 0 1 0 0 1 1 1 in population with unknown status 1 : 0
Number of results 1 0 1 0 0 1 1 1 in population with unknown status 1 : 0
Number of results 0 1 1 0 0 1 1 1 in population with unknown status 1 : 0
Number of results 1 1 1 0 0 1 1 1 in population with unknown status 1 : 8
Number of results 0 0 0 1 0 1 1 1 in population with unknown status 1 : 0
Number of results 1 0 0 1 0 1 1 1 in population with unknown status 1 : 1
Number of results 0 1 0 1 0 1 1 1 in population with unknown status 1 : 0
Number of results 1 1 0 1 0 1 1 1 in population with unknown status 1 : 0
Number of results 0 0 1 1 0 1 1 1 in population with unknown status 1 : 0
Number of results 1 0 1 1 0 1 1 1 in population with unknown status 1 : 0
Number of results 0 1 1 1 0 1 1 1 in population with unknown status 1 : 0
Number of results 1 1 1 1 0 1 1 1 in population with unknown status 1 : 17
Number of results 0 0 0 0 1 1 1 1 in population with unknown status 1 : 0
Number of results 1 0 0 0 1 1 1 1 in population with unknown status 1 : 0

Number of results 0 1 0 0 1 1 1 1 in population with unknown status 1 : 0
Number of results 1 1 0 0 1 1 1 1 in population with unknown status 1 : 0
Number of results 0 0 1 0 1 1 1 1 in population with unknown status 1 : 0
Number of results 1 0 1 0 1 1 1 1 in population with unknown status 1 : 0
Number of results 0 1 1 0 1 1 1 1 in population with unknown status 1 : 0
Number of results 1 1 1 0 1 1 1 1 in population with unknown status 1 : 0
Number of results 0 0 0 1 1 1 1 1 in population with unknown status 1 : 0
Number of results 1 0 0 1 1 1 1 1 in population with unknown status 1 : 0
Number of results 0 1 0 1 1 1 1 1 in population with unknown status 1 : 0
Number of results 1 1 0 1 1 1 1 1 in population with unknown status 1 : 0
Number of results 0 0 1 1 1 1 1 1 in population with unknown status 1 : 0
Number of results 1 0 1 1 1 1 1 1 in population with unknown status 1 : 0
Number of results 0 1 1 1 1 1 1 1 in population with unknown status 1 : 0
Number of results 1 1 1 1 1 1 1 1 in population with unknown status 1 : 75

DATA SUMMARY
1 Population(s); 8 Tests; 0 Reference Population(s)

df: 255 ; parameters: 17

	test1	test2	test3	test4	test5	test6	test7	test8	pop1	RefInd	RefInf
1	0	0	0	0	0	0	0	0	398	0	0
2	1	0	0	0	0	0	0	0	0	0	0
3	0	1	0	0	0	0	0	0	0	0	0
4	1	1	0	0	0	0	0	0	0	0	0
5	0	0	1	0	0	0	0	0	0	0	0
6	1	0	1	0	0	0	0	0	0	0	0
7	0	1	1	0	0	0	0	0	0	0	0
8	1	1	1	0	0	0	0	0	0	0	0
9	0	0	0	1	0	0	0	0	0	0	0
10	1	0	0	1	0	0	0	0	0	0	0
11	0	1	0	1	0	0	0	0	0	0	0
12	1	1	0	1	0	0	0	0	0	0	0
13	0	0	1	1	0	0	0	0	0	0	0
14	1	0	1	1	0	0	0	0	0	0	0
15	0	1	1	1	0	0	0	0	0	0	0
16	1	1	1	1	0	0	0	0	0	0	0
17	0	0	0	0	1	0	0	0	1	0	0
18	1	0	0	0	1	0	0	0	0	0	0
19	0	1	0	0	1	0	0	0	0	0	0
20	1	1	0	0	1	0	0	0	0	0	0
21	0	0	1	0	1	0	0	0	0	0	0
22	1	0	1	0	1	0	0	0	0	0	0

23	0	1	1	0	1	0	0	0	0	0	0
24	1	1	1	0	1	0	0	0	1	0	0
25	0	0	0	1	1	0	0	0	0	0	0
26	1	0	0	1	1	0	0	0	0	0	0
27	0	1	0	1	1	0	0	0	0	0	0
28	1	1	0	1	1	0	0	0	0	0	0
29	0	0	1	1	1	0	0	0	0	0	0
30	1	0	1	1	1	0	0	0	0	0	0
31	0	1	1	1	1	0	0	0	0	0	0
32	1	1	1	1	1	0	0	0	0	0	0
33	0	0	0	0	0	1	0	0	0	0	0
34	1	0	0	0	0	1	0	0	0	0	0
35	0	1	0	0	0	1	0	0	0	0	0
36	1	1	0	0	0	1	0	0	0	0	0
37	0	0	1	0	0	1	0	0	0	0	0
38	1	0	1	0	0	1	0	0	0	0	0
39	0	1	1	0	0	1	0	0	0	0	0
40	1	1	1	0	0	1	0	0	0	0	0
41	0	0	0	1	0	1	0	0	0	0	0
42	1	0	0	1	0	1	0	0	0	0	0
43	0	1	0	1	0	1	0	0	0	0	0
44	1	1	0	1	0	1	0	0	0	0	0
45	0	0	1	1	0	1	0	0	0	0	0
46	1	0	1	1	0	1	0	0	0	0	0
47	0	1	1	1	0	1	0	0	0	0	0
48	1	1	1	1	0	1	0	0	0	0	0
49	0	0	0	0	1	1	0	0	0	0	0
50	1	0	0	0	1	1	0	0	0	0	0
51	0	1	0	0	1	1	0	0	0	0	0
52	1	1	0	0	1	1	0	0	0	0	0
53	0	0	1	0	1	1	0	0	0	0	0
54	1	0	1	0	1	1	0	0	0	0	0
55	0	1	1	0	1	1	0	0	0	0	0
56	1	1	1	0	1	1	0	0	0	0	0
57	0	0	0	1	1	1	0	0	0	0	0
58	1	0	0	1	1	1	0	0	0	0	0
59	0	1	0	1	1	1	0	0	0	0	0
60	1	1	0	1	1	1	0	0	0	0	0
61	0	0	1	1	1	1	0	0	0	0	0
62	1	0	1	1	1	1	0	0	0	0	0
63	0	1	1	1	1	1	0	0	0	0	0
64	1	1	1	1	1	1	0	0	0	0	0
65	0	0	0	0	0	0	1	0	0	0	0
66	1	0	0	0	0	0	1	0	0	0	0

67	0	1	0	0	0	0	1	0	0	0	0
68	1	1	0	0	0	0	1	0	0	0	0
69	0	0	1	0	0	0	1	0	0	0	0
70	1	0	1	0	0	0	1	0	0	0	0
71	0	1	1	0	0	0	1	0	0	0	0
72	1	1	1	0	0	0	1	0	0	0	0
73	0	0	0	1	0	0	1	0	0	0	0
74	1	0	0	1	0	0	1	0	0	0	0
75	0	1	0	1	0	0	1	0	0	0	0
76	1	1	0	1	0	0	1	0	0	0	0
77	0	0	1	1	0	0	1	0	0	0	0
78	1	0	1	1	0	0	1	0	0	0	0
79	0	1	1	1	0	0	1	0	0	0	0
80	1	1	1	1	0	0	1	0	0	0	0
81	0	0	0	0	1	0	1	0	0	0	0
82	1	0	0	0	1	0	1	0	0	0	0
83	0	1	0	0	1	0	1	0	0	0	0
84	1	1	0	0	1	0	1	0	0	0	0
85	0	0	1	0	1	0	1	0	0	0	0
86	1	0	1	0	1	0	1	0	0	0	0
87	0	1	1	0	1	0	1	0	0	0	0
88	1	1	1	0	1	0	1	0	0	0	0
89	0	0	0	1	1	0	1	0	0	0	0
90	1	0	0	1	1	0	1	0	0	0	0
91	0	1	0	1	1	0	1	0	0	0	0
92	1	1	0	1	1	0	1	0	0	0	0
93	0	0	1	1	1	0	1	0	0	0	0
94	1	0	1	1	1	0	1	0	0	0	0
95	0	1	1	1	1	0	1	0	0	0	0
96	1	1	1	1	1	0	1	0	0	0	0
97	0	0	0	0	0	1	1	0	0	0	0
98	1	0	0	0	0	1	1	0	0	0	0
99	0	1	0	0	0	1	1	0	0	0	0
100	1	1	0	0	0	1	1	0	0	0	0
101	0	0	1	0	0	1	1	0	0	0	0
102	1	0	1	0	0	1	1	0	0	0	0
103	0	1	1	0	0	1	1	0	0	0	0
104	1	1	1	0	0	1	1	0	0	0	0
105	0	0	0	1	0	1	1	0	0	0	0
106	1	0	0	1	0	1	1	0	0	0	0
107	0	1	0	1	0	1	1	0	0	0	0
108	1	1	0	1	0	1	1	0	0	0	0
109	0	0	1	1	0	1	1	0	3	0	0
110	1	0	1	1	0	1	1	0	0	0	0

111	0	1	1	1	0	1	1	0	0	0	0
112	1	1	1	1	0	1	1	0	0	0	0
113	0	0	0	0	1	1	1	0	0	0	0
114	1	0	0	0	1	1	1	0	0	0	0
115	0	1	0	0	1	1	1	0	0	0	0
116	1	1	0	0	1	1	1	0	0	0	0
117	0	0	1	0	1	1	1	0	0	0	0
118	1	0	1	0	1	1	1	0	0	0	0
119	0	1	1	0	1	1	1	0	0	0	0
120	1	1	1	0	1	1	1	0	0	0	0
121	0	0	0	1	1	1	1	0	1	0	0
122	1	0	0	1	1	1	1	0	0	0	0
123	0	1	0	1	1	1	1	0	0	0	0
124	1	1	0	1	1	1	1	0	0	0	0
125	0	0	1	1	1	1	1	0	2	0	0
126	1	0	1	1	1	1	1	0	0	0	0
127	0	1	1	1	1	1	1	0	0	0	0
128	1	1	1	1	1	1	1	0	0	0	0
129	0	0	0	0	0	0	0	1	0	0	0
130	1	0	0	0	0	0	0	1	0	0	0
131	0	1	0	0	0	0	0	1	0	0	0
132	1	1	0	0	0	0	0	1	0	0	0
133	0	0	1	0	0	0	0	1	0	0	0
134	1	0	1	0	0	0	0	1	0	0	0
135	0	1	1	0	0	0	0	1	0	0	0
136	1	1	1	0	0	0	0	1	0	0	0
137	0	0	0	1	0	0	0	1	0	0	0
138	1	0	0	1	0	0	0	1	0	0	0
139	0	1	0	1	0	0	0	1	0	0	0
140	1	1	0	1	0	0	0	1	0	0	0
141	0	0	1	1	0	0	0	1	0	0	0
142	1	0	1	1	0	0	0	1	0	0	0
143	0	1	1	1	0	0	0	1	0	0	0
144	1	1	1	1	0	0	0	1	0	0	0
145	0	0	0	0	1	0	0	1	0	0	0
146	1	0	0	0	1	0	0	1	0	0	0
147	0	1	0	0	1	0	0	1	0	0	0
148	1	1	0	0	1	0	0	1	0	0	0
149	0	0	1	0	1	0	0	1	0	0	0
150	1	0	1	0	1	0	0	1	0	0	0
151	0	1	1	0	1	0	0	1	0	0	0
152	1	1	1	0	1	0	0	1	0	0	0
153	0	0	0	1	1	0	0	1	0	0	0
154	1	0	0	1	1	0	0	1	0	0	0

155	0	1	0	1	1	0	0	1	0	0	0
156	1	1	0	1	1	0	0	1	0	0	0
157	0	0	1	1	1	0	0	1	0	0	0
158	1	0	1	1	1	0	0	1	0	0	0
159	0	1	1	1	1	0	0	1	0	0	0
160	1	1	1	1	1	0	0	1	0	0	0
161	0	0	0	0	0	1	0	1	0	0	0
162	1	0	0	0	0	1	0	1	0	0	0
163	0	1	0	0	0	1	0	1	10	0	0
164	1	1	0	0	0	1	0	1	0	0	0
165	0	0	1	0	0	1	0	1	0	0	0
166	1	0	1	0	0	1	0	1	0	0	0
167	0	1	1	0	0	1	0	1	0	0	0
168	1	1	1	0	0	1	0	1	0	0	0
169	0	0	0	1	0	1	0	1	0	0	0
170	1	0	0	1	0	1	0	1	0	0	0
171	0	1	0	1	0	1	0	1	0	0	0
172	1	1	0	1	0	1	0	1	0	0	0
173	0	0	1	1	0	1	0	1	0	0	0
174	1	0	1	1	0	1	0	1	0	0	0
175	0	1	1	1	0	1	0	1	0	0	0
176	1	1	1	1	0	1	0	1	0	0	0
177	0	0	0	0	1	1	0	1	0	0	0
178	1	0	0	0	1	1	0	1	0	0	0
179	0	1	0	0	1	1	0	1	0	0	0
180	1	1	0	0	1	1	0	1	0	0	0
181	0	0	1	0	1	1	0	1	0	0	0
182	1	0	1	0	1	1	0	1	0	0	0
183	0	1	1	0	1	1	0	1	0	0	0
184	1	1	1	0	1	1	0	1	0	0	0
185	0	0	0	1	1	1	0	1	0	0	0
186	1	0	0	1	1	1	0	1	0	0	0
187	0	1	0	1	1	1	0	1	0	0	0
188	1	1	0	1	1	1	0	1	0	0	0
189	0	0	1	1	1	1	0	1	0	0	0
190	1	0	1	1	1	1	0	1	0	0	0
191	0	1	1	1	1	1	0	1	0	0	0
192	1	1	1	1	1	1	0	1	0	0	0
193	0	0	0	0	0	0	1	1	6	0	0
194	1	0	0	0	0	0	1	1	0	0	0
195	0	1	0	0	0	0	1	1	0	0	0
196	1	1	0	0	0	0	1	1	0	0	0
197	0	0	1	0	0	0	1	1	20	0	0
198	1	0	1	0	0	0	1	1	0	0	0

199	0	1	1	0	0	0	1	1	0	0	0
200	1	1	1	0	0	0	1	1	0	0	0
201	0	0	0	1	0	0	1	1	0	0	0
202	1	0	0	1	0	0	1	1	0	0	0
203	0	1	0	1	0	0	1	1	0	0	0
204	1	1	0	1	0	0	1	1	0	0	0
205	0	0	1	1	0	0	1	1	0	0	0
206	1	0	1	1	0	0	1	1	0	0	0
207	0	1	1	1	0	0	1	1	0	0	0
208	1	1	1	1	0	0	1	1	0	0	0
209	0	0	0	0	1	0	1	1	0	0	0
210	1	0	0	0	1	0	1	1	0	0	0
211	0	1	0	0	1	0	1	1	0	0	0
212	1	1	0	0	1	0	1	1	0	0	0
213	0	0	1	0	1	0	1	1	0	0	0
214	1	0	1	0	1	0	1	1	0	0	0
215	0	1	1	0	1	0	1	1	0	0	0
216	1	1	1	0	1	0	1	1	0	0	0
217	0	0	0	1	1	0	1	1	0	0	0
218	1	0	0	1	1	0	1	1	0	0	0
219	0	1	0	1	1	0	1	1	0	0	0
220	1	1	0	1	1	0	1	1	0	0	0
221	0	0	1	1	1	0	1	1	0	0	0
222	1	0	1	1	1	0	1	1	0	0	0
223	0	1	1	1	1	0	1	1	0	0	0
224	1	1	1	1	1	0	1	1	0	0	0
225	0	0	0	0	0	1	1	1	0	0	0
226	1	0	0	0	0	1	1	1	0	0	0
227	0	1	0	0	0	1	1	1	0	0	0
228	1	1	0	0	0	1	1	1	0	0	0
229	0	0	1	0	0	1	1	1	0	0	0
230	1	0	1	0	0	1	1	1	0	0	0
231	0	1	1	0	0	1	1	1	0	0	0
232	1	1	1	0	0	1	1	1	8	0	0
233	0	0	0	1	0	1	1	1	0	0	0
234	1	0	0	1	0	1	1	1	1	0	0
235	0	1	0	1	0	1	1	1	0	0	0
236	1	1	0	1	0	1	1	1	0	0	0
237	0	0	1	1	0	1	1	1	0	0	0
238	1	0	1	1	0	1	1	1	0	0	0
239	0	1	1	1	0	1	1	1	0	0	0
240	1	1	1	1	0	1	1	1	17	0	0
241	0	0	0	0	1	1	1	1	0	0	0
242	1	0	0	0	1	1	1	1	0	0	0

243	0	1	0	0	1	1	1	1	0	0	0
244	1	1	0	0	1	1	1	1	0	0	0
245	0	0	1	0	1	1	1	1	0	0	0
246	1	0	1	0	1	1	1	1	0	0	0
247	0	1	1	0	1	1	1	1	0	0	0
248	1	1	1	0	1	1	1	1	0	0	0
249	0	0	0	1	1	1	1	1	0	0	0
250	1	0	0	1	1	1	1	1	0	0	0
251	0	1	0	1	1	1	1	1	0	0	0
252	1	1	0	1	1	1	1	1	0	0	0
253	0	0	1	1	1	1	1	1	0	0	0
254	1	0	1	1	1	1	1	1	0	0	0
255	0	1	1	1	1	1	1	1	0	0	0
256	1	1	1	1	1	1	1	1	75	0	0

Is it OK ?
No : Enter 0
Yes : Enter 1
1

Default Best Guess:

	pre1	Sp1	Sp2	Sp3	Sp4	Sp5	Sp6	Sp7	Sp8	Se1	Se2	Se3	Se4	Se5	Se6
Best Guess	0.2	0.95	0.95	0.95	0.95	0.95	0.95	0.95	0.95	0.8	0.8	0.8	0.8	0.8	0.8

	Se7	Se8
Best Guess	0.8	0.8

Do you have Best Guess?
No : Enter 0
Yes : Enter 1
0

Would you like Bootstrap Confidence intervals
(CAUTION: this may be time consumming. It is recommended to ask for it in a second step!)
No : Enter 0
Yes : Enter 1
1
How many iterations? (between 50 and 5000)
5000

DATA SUMMARY
1 Population(s); 8 Tests; 0 Reference Population(s)

df: 255 ; parameters: 17

	test1	test2	test3	test4	test5	test6	test7	test8	pop1	RefInd	RefInf
1	0	0	0	0	0	0	0	0	398	0	0
2	1	0	0	0	0	0	0	0	0	0	0
3	0	1	0	0	0	0	0	0	0	0	0
4	1	1	0	0	0	0	0	0	0	0	0
5	0	0	1	0	0	0	0	0	0	0	0
6	1	0	1	0	0	0	0	0	0	0	0
7	0	1	1	0	0	0	0	0	0	0	0
8	1	1	1	0	0	0	0	0	0	0	0
9	0	0	0	1	0	0	0	0	0	0	0
10	1	0	0	1	0	0	0	0	0	0	0
11	0	1	0	1	0	0	0	0	0	0	0
12	1	1	0	1	0	0	0	0	0	0	0
13	0	0	1	1	0	0	0	0	0	0	0
14	1	0	1	1	0	0	0	0	0	0	0
15	0	1	1	1	0	0	0	0	0	0	0
16	1	1	1	1	0	0	0	0	0	0	0
17	0	0	0	0	1	0	0	0	1	0	0
18	1	0	0	0	1	0	0	0	0	0	0
19	0	1	0	0	1	0	0	0	0	0	0
20	1	1	0	0	1	0	0	0	0	0	0
21	0	0	1	0	1	0	0	0	0	0	0
22	1	0	1	0	1	0	0	0	0	0	0
23	0	1	1	0	1	0	0	0	0	0	0
24	1	1	1	0	1	0	0	0	1	0	0
25	0	0	0	1	1	0	0	0	0	0	0
26	1	0	0	1	1	0	0	0	0	0	0
27	0	1	0	1	1	0	0	0	0	0	0
28	1	1	0	1	1	0	0	0	0	0	0
29	0	0	1	1	1	0	0	0	0	0	0
30	1	0	1	1	1	0	0	0	0	0	0
31	0	1	1	1	1	0	0	0	0	0	0
32	1	1	1	1	1	0	0	0	0	0	0
33	0	0	0	0	0	1	0	0	0	0	0
34	1	0	0	0	0	1	0	0	0	0	0
35	0	1	0	0	0	1	0	0	0	0	0
36	1	1	0	0	0	1	0	0	0	0	0
37	0	0	1	0	0	1	0	0	0	0	0
38	1	0	1	0	0	1	0	0	0	0	0
39	0	1	1	0	0	1	0	0	0	0	0
40	1	1	1	0	0	1	0	0	0	0	0
41	0	0	0	1	0	1	0	0	0	0	0

42	1	0	0	1	0	1	0	0	0	0	0
43	0	1	0	1	0	1	0	0	0	0	0
44	1	1	0	1	0	1	0	0	0	0	0
45	0	0	1	1	0	1	0	0	0	0	0
46	1	0	1	1	0	1	0	0	0	0	0
47	0	1	1	1	0	1	0	0	0	0	0
48	1	1	1	1	0	1	0	0	0	0	0
49	0	0	0	0	1	1	0	0	0	0	0
50	1	0	0	0	1	1	0	0	0	0	0
51	0	1	0	0	1	1	0	0	0	0	0
52	1	1	0	0	1	1	0	0	0	0	0
53	0	0	1	0	1	1	0	0	0	0	0
54	1	0	1	0	1	1	0	0	0	0	0
55	0	1	1	0	1	1	0	0	0	0	0
56	1	1	1	0	1	1	0	0	0	0	0
57	0	0	0	1	1	1	0	0	0	0	0
58	1	0	0	1	1	1	0	0	0	0	0
59	0	1	0	1	1	1	0	0	0	0	0
60	1	1	0	1	1	1	0	0	0	0	0
61	0	0	1	1	1	1	0	0	0	0	0
62	1	0	1	1	1	1	0	0	0	0	0
63	0	1	1	1	1	1	0	0	0	0	0
64	1	1	1	1	1	1	0	0	0	0	0
65	0	0	0	0	0	0	1	0	0	0	0
66	1	0	0	0	0	0	1	0	0	0	0
67	0	1	0	0	0	0	1	0	0	0	0
68	1	1	0	0	0	0	1	0	0	0	0
69	0	0	1	0	0	0	1	0	0	0	0
70	1	0	1	0	0	0	1	0	0	0	0
71	0	1	1	0	0	0	1	0	0	0	0
72	1	1	1	0	0	0	1	0	0	0	0
73	0	0	0	1	0	0	1	0	0	0	0
74	1	0	0	1	0	0	1	0	0	0	0
75	0	1	0	1	0	0	1	0	0	0	0
76	1	1	0	1	0	0	1	0	0	0	0
77	0	0	1	1	0	0	1	0	0	0	0
78	1	0	1	1	0	0	1	0	0	0	0
79	0	1	1	1	0	0	1	0	0	0	0
80	1	1	1	1	0	0	1	0	0	0	0
81	0	0	0	0	1	0	1	0	0	0	0
82	1	0	0	0	1	0	1	0	0	0	0
83	0	1	0	0	1	0	1	0	0	0	0
84	1	1	0	0	1	0	1	0	0	0	0
85	0	0	1	0	1	0	1	0	0	0	0

86	1	0	1	0	1	0	1	0	0	0	0
87	0	1	1	0	1	0	1	0	0	0	0
88	1	1	1	0	1	0	1	0	0	0	0
89	0	0	0	1	1	0	1	0	0	0	0
90	1	0	0	1	1	0	1	0	0	0	0
91	0	1	0	1	1	0	1	0	0	0	0
92	1	1	0	1	1	0	1	0	0	0	0
93	0	0	1	1	1	0	1	0	0	0	0
94	1	0	1	1	1	0	1	0	0	0	0
95	0	1	1	1	1	0	1	0	0	0	0
96	1	1	1	1	1	0	1	0	0	0	0
97	0	0	0	0	0	1	1	0	0	0	0
98	1	0	0	0	0	1	1	0	0	0	0
99	0	1	0	0	0	1	1	0	0	0	0
100	1	1	0	0	0	1	1	0	0	0	0
101	0	0	1	0	0	1	1	0	0	0	0
102	1	0	1	0	0	1	1	0	0	0	0
103	0	1	1	0	0	1	1	0	0	0	0
104	1	1	1	0	0	1	1	0	0	0	0
105	0	0	0	1	0	1	1	0	0	0	0
106	1	0	0	1	0	1	1	0	0	0	0
107	0	1	0	1	0	1	1	0	0	0	0
108	1	1	0	1	0	1	1	0	0	0	0
109	0	0	1	1	0	1	1	0	3	0	0
110	1	0	1	1	0	1	1	0	0	0	0
111	0	1	1	1	0	1	1	0	0	0	0
112	1	1	1	1	0	1	1	0	0	0	0
113	0	0	0	0	1	1	1	0	0	0	0
114	1	0	0	0	1	1	1	0	0	0	0
115	0	1	0	0	1	1	1	0	0	0	0
116	1	1	0	0	1	1	1	0	0	0	0
117	0	0	1	0	1	1	1	0	0	0	0
118	1	0	1	0	1	1	1	0	0	0	0
119	0	1	1	0	1	1	1	0	0	0	0
120	1	1	1	0	1	1	1	0	0	0	0
121	0	0	0	1	1	1	1	0	1	0	0
122	1	0	0	1	1	1	1	0	0	0	0
123	0	1	0	1	1	1	1	0	0	0	0
124	1	1	0	1	1	1	1	0	0	0	0
125	0	0	1	1	1	1	1	0	2	0	0
126	1	0	1	1	1	1	1	0	0	0	0
127	0	1	1	1	1	1	1	0	0	0	0
128	1	1	1	1	1	1	1	0	0	0	0
129	0	0	0	0	0	0	0	1	0	0	0

130	1	0	0	0	0	0	0	1	0	0	0
131	0	1	0	0	0	0	0	1	0	0	0
132	1	1	0	0	0	0	0	1	0	0	0
133	0	0	1	0	0	0	0	1	0	0	0
134	1	0	1	0	0	0	0	1	0	0	0
135	0	1	1	0	0	0	0	1	0	0	0
136	1	1	1	0	0	0	0	1	0	0	0
137	0	0	0	1	0	0	0	1	0	0	0
138	1	0	0	1	0	0	0	1	0	0	0
139	0	1	0	1	0	0	0	1	0	0	0
140	1	1	0	1	0	0	0	1	0	0	0
141	0	0	1	1	0	0	0	1	0	0	0
142	1	0	1	1	0	0	0	1	0	0	0
143	0	1	1	1	0	0	0	1	0	0	0
144	1	1	1	1	0	0	0	1	0	0	0
145	0	0	0	0	1	0	0	1	0	0	0
146	1	0	0	0	1	0	0	1	0	0	0
147	0	1	0	0	1	0	0	1	0	0	0
148	1	1	0	0	1	0	0	1	0	0	0
149	0	0	1	0	1	0	0	1	0	0	0
150	1	0	1	0	1	0	0	1	0	0	0
151	0	1	1	0	1	0	0	1	0	0	0
152	1	1	1	0	1	0	0	1	0	0	0
153	0	0	0	1	1	0	0	1	0	0	0
154	1	0	0	1	1	0	0	1	0	0	0
155	0	1	0	1	1	0	0	1	0	0	0
156	1	1	0	1	1	0	0	1	0	0	0
157	0	0	1	1	1	0	0	1	0	0	0
158	1	0	1	1	1	0	0	1	0	0	0
159	0	1	1	1	1	0	0	1	0	0	0
160	1	1	1	1	1	0	0	1	0	0	0
161	0	0	0	0	0	1	0	1	0	0	0
162	1	0	0	0	0	1	0	1	0	0	0
163	0	1	0	0	0	1	0	1	10	0	0
164	1	1	0	0	0	1	0	1	0	0	0
165	0	0	1	0	0	1	0	1	0	0	0
166	1	0	1	0	0	1	0	1	0	0	0
167	0	1	1	0	0	1	0	1	0	0	0
168	1	1	1	0	0	1	0	1	0	0	0
169	0	0	0	1	0	1	0	1	0	0	0
170	1	0	0	1	0	1	0	1	0	0	0
171	0	1	0	1	0	1	0	1	0	0	0
172	1	1	0	1	0	1	0	1	0	0	0
173	0	0	1	1	0	1	0	1	0	0	0

174	1	0	1	1	0	1	0	1	0	0	0
175	0	1	1	1	0	1	0	1	0	0	0
176	1	1	1	1	0	1	0	1	0	0	0
177	0	0	0	0	1	1	0	1	0	0	0
178	1	0	0	0	1	1	0	1	0	0	0
179	0	1	0	0	1	1	0	1	0	0	0
180	1	1	0	0	1	1	0	1	0	0	0
181	0	0	1	0	1	1	0	1	0	0	0
182	1	0	1	0	1	1	0	1	0	0	0
183	0	1	1	0	1	1	0	1	0	0	0
184	1	1	1	0	1	1	0	1	0	0	0
185	0	0	0	1	1	1	0	1	0	0	0
186	1	0	0	1	1	1	0	1	0	0	0
187	0	1	0	1	1	1	0	1	0	0	0
188	1	1	0	1	1	1	0	1	0	0	0
189	0	0	1	1	1	1	0	1	0	0	0
190	1	0	1	1	1	1	0	1	0	0	0
191	0	1	1	1	1	1	0	1	0	0	0
192	1	1	1	1	1	1	0	1	0	0	0
193	0	0	0	0	0	0	1	1	6	0	0
194	1	0	0	0	0	0	1	1	0	0	0
195	0	1	0	0	0	0	1	1	0	0	0
196	1	1	0	0	0	0	1	1	0	0	0
197	0	0	1	0	0	0	1	1	20	0	0
198	1	0	1	0	0	0	1	1	0	0	0
199	0	1	1	0	0	0	1	1	0	0	0
200	1	1	1	0	0	0	1	1	0	0	0
201	0	0	0	1	0	0	1	1	0	0	0
202	1	0	0	1	0	0	1	1	0	0	0
203	0	1	0	1	0	0	1	1	0	0	0
204	1	1	0	1	0	0	1	1	0	0	0
205	0	0	1	1	0	0	1	1	0	0	0
206	1	0	1	1	0	0	1	1	0	0	0
207	0	1	1	1	0	0	1	1	0	0	0
208	1	1	1	1	0	0	1	1	0	0	0
209	0	0	0	0	1	0	1	1	0	0	0
210	1	0	0	0	1	0	1	1	0	0	0
211	0	1	0	0	1	0	1	1	0	0	0
212	1	1	0	0	1	0	1	1	0	0	0
213	0	0	1	0	1	0	1	1	0	0	0
214	1	0	1	0	1	0	1	1	0	0	0
215	0	1	1	0	1	0	1	1	0	0	0
216	1	1	1	0	1	0	1	1	0	0	0
217	0	0	0	1	1	0	1	1	0	0	0

218	1	0	0	1	1	0	1	1	0	0	0
219	0	1	0	1	1	0	1	1	0	0	0
220	1	1	0	1	1	0	1	1	0	0	0
221	0	0	1	1	1	0	1	1	0	0	0
222	1	0	1	1	1	0	1	1	0	0	0
223	0	1	1	1	1	0	1	1	0	0	0
224	1	1	1	1	1	0	1	1	0	0	0
225	0	0	0	0	0	1	1	1	0	0	0
226	1	0	0	0	0	1	1	1	0	0	0
227	0	1	0	0	0	1	1	1	0	0	0
228	1	1	0	0	0	1	1	1	0	0	0
229	0	0	1	0	0	1	1	1	0	0	0
230	1	0	1	0	0	1	1	1	0	0	0
231	0	1	1	0	0	1	1	1	0	0	0
232	1	1	1	0	0	1	1	1	8	0	0
233	0	0	0	1	0	1	1	1	0	0	0
234	1	0	0	1	0	1	1	1	1	0	0
235	0	1	0	1	0	1	1	1	0	0	0
236	1	1	0	1	0	1	1	1	0	0	0
237	0	0	1	1	0	1	1	1	0	0	0
238	1	0	1	1	0	1	1	1	0	0	0
239	0	1	1	1	0	1	1	1	0	0	0
240	1	1	1	1	0	1	1	1	17	0	0
241	0	0	0	0	1	1	1	1	0	0	0
242	1	0	0	0	1	1	1	1	0	0	0
243	0	1	0	0	1	1	1	1	0	0	0
244	1	1	0	0	1	1	1	1	0	0	0
245	0	0	1	0	1	1	1	1	0	0	0
246	1	0	1	0	1	1	1	1	0	0	0
247	0	1	1	0	1	1	1	1	0	0	0
248	1	1	1	0	1	1	1	1	0	0	0
249	0	0	0	1	1	1	1	1	0	0	0
250	1	0	0	1	1	1	1	1	0	0	0
251	0	1	0	1	1	1	1	1	0	0	0
252	1	1	0	1	1	1	1	1	0	0	0
253	0	0	1	1	1	1	1	1	0	0	0
254	1	0	1	1	1	1	1	1	0	0	0
255	0	1	1	1	1	1	1	1	0	0	0
256	1	1	1	1	1	1	1	1	75	0	0

	pre1	Sp1	Sp2	Sp3	Sp4	Sp5	Sp6	Sp7	Sp8	Se1	Se2	Se3	Se4	Se5	Se6
Best Guess	0.2	0.95	0.95	0.95	0.95	0.95	0.95	0.95	0.95	0.8	0.8	0.8	0.8	0.8	0.8

	Se7	Se8
Best Guess	0.8	0.8

EXPECTATION MAXIMISATION

```
$Iterations
[1] 8

$LogLikelihood
[1] -867.0493

$Estimations
     pre1 Sp1   Sp2    Sp3 Sp4    Sp5   Sp6    Sp7    Sp8    Se1    Se2    Se3
Est 0.1989   1 0.977 0.9541   1 0.9977 0.977 0.9403 0.9173 0.9442 0.9351 0.9814
       Se4    Se5    Se6    Se7    Se8
Est 0.9164 0.7313 0.9906 0.9906 0.9352
```

NEWTON-RAPHSON

```
$Iterations
[1] 24

$LogLikelihood
[1] -867.0493

$Estimations
        pre1 Sp1    Sp2    Sp3 Sp4    Sp5    Sp6    Sp7    Sp8    Se1    Se2
Est   0.1989   1 0.9770 0.9541   1 0.9977 0.9770 0.9403 0.9173 0.9442 0.9351
CIinf 0.1675 NaN 0.9578 0.9298 NaN 0.9839 0.9578 0.9137 0.8875 0.8813 0.8700
CIsup 0.2347   1 0.9876 0.9702   1 0.9997 0.9876 0.9590 0.9398 0.9747 0.9687
         Se3    Se4    Se5    Se6    Se7    Se8
Est   0.9814 0.9164 0.7313 0.9906 0.9906 0.9352
CIinf 0.9285 0.8471 0.6400 0.9362 0.9364 0.8702
CIsup 0.9953 0.9560 0.8064 0.9987 0.9987 0.9688
```

WARNING 1: test results are assumed to be independent conditional on infection or disease status

Expected Results (NR) and Goodness-of-fit test

$Expected

	test1	test2	test3	test4	test5	test6	test7	test8	pop1	RefInd	RefInf	ExpPop1
1	0	0	0	0	0	0	0	0	398	0	0	340.89
2	1	0	0	0	0	0	0	0	0	0	0	0.00
3	0	1	0	0	0	0	0	0	0	0	0	8.01
4	1	1	0	0	0	0	0	0	0	0	0	0.00
5	0	0	1	0	0	0	0	0	0	0	0	16.42

6	1	0	1	0	0	0	0	0	0	0	0	0.00
7	0	1	1	0	0	0	0	0	0	0	0	0.39
8	1	1	1	0	0	0	0	0	0	0	0	0.00
9	0	0	0	1	0	0	0	0	0	0	0	0.00
10	1	0	0	1	0	0	0	0	0	0	0	0.00
11	0	1	0	1	0	0	0	0	0	0	0	0.00
12	1	1	0	1	0	0	0	0	0	0	0	0.00
13	0	0	1	1	0	0	0	0	0	0	0	0.00
14	1	0	1	1	0	0	0	0	0	0	0	0.00
15	0	1	1	1	0	0	0	0	0	0	0	0.00
16	1	1	1	1	0	0	0	0	0	0	0	0.00
17	0	0	0	0	1	0	0	0	1	0	0	0.79
18	1	0	0	0	1	0	0	0	0	0	0	0.00
19	0	1	0	0	1	0	0	0	0	0	0	0.02
20	1	1	0	0	1	0	0	0	0	0	0	0.00
21	0	0	1	0	1	0	0	0	0	0	0	0.04
22	1	0	1	0	1	0	0	0	0	0	0	0.00
23	0	1	1	0	1	0	0	0	0	0	0	0.00
24	1	1	1	0	1	0	0	0	1	0	0	0.00
25	0	0	0	1	1	0	0	0	0	0	0	0.00
26	1	0	0	1	1	0	0	0	0	0	0	0.00
27	0	1	0	1	1	0	0	0	0	0	0	0.00
28	1	1	0	1	1	0	0	0	0	0	0	0.00
29	0	0	1	1	1	0	0	0	0	0	0	0.00
30	1	0	1	1	1	0	0	0	0	0	0	0.00
31	0	1	1	1	1	0	0	0	0	0	0	0.00
32	1	1	1	1	1	0	0	0	0	0	0	0.00
33	0	0	0	0	0	1	0	0	0	0	0	8.01
34	1	0	0	0	0	1	0	0	0	0	0	0.00
35	0	1	0	0	0	1	0	0	0	0	0	0.19
36	1	1	0	0	0	1	0	0	0	0	0	0.00
37	0	0	1	0	0	1	0	0	0	0	0	0.39
38	1	0	1	0	0	1	0	0	0	0	0	0.00
39	0	1	1	0	0	1	0	0	0	0	0	0.01
40	1	1	1	0	0	1	0	0	0	0	0	0.00
41	0	0	0	1	0	1	0	0	0	0	0	0.00
42	1	0	0	1	0	1	0	0	0	0	0	0.00
43	0	1	0	1	0	1	0	0	0	0	0	0.00
44	1	1	0	1	0	1	0	0	0	0	0	0.00
45	0	0	1	1	0	1	0	0	0	0	0	0.00
46	1	0	1	1	0	1	0	0	0	0	0	0.00
47	0	1	1	1	0	1	0	0	0	0	0	0.00
48	1	1	1	1	0	1	0	0	0	0	0	0.01
49	0	0	0	0	1	1	0	0	0	0	0	0.02

50	1	0	0	0	1	1	0	0	0	0	0	0.00
51	0	1	0	0	1	1	0	0	0	0	0	0.00
52	1	1	0	0	1	1	0	0	0	0	0	0.00
53	0	0	1	0	1	1	0	0	0	0	0	0.00
54	1	0	1	0	1	1	0	0	0	0	0	0.00
55	0	1	1	0	1	1	0	0	0	0	0	0.00
56	1	1	1	0	1	1	0	0	0	0	0	0.00
57	0	0	0	1	1	1	0	0	0	0	0	0.00
58	1	0	0	1	1	1	0	0	0	0	0	0.00
59	0	1	0	1	1	1	0	0	0	0	0	0.00
60	1	1	0	1	1	1	0	0	0	0	0	0.00
61	0	0	1	1	1	1	0	0	0	0	0	0.00
62	1	0	1	1	1	1	0	0	0	0	0	0.00
63	0	1	1	1	1	1	0	0	0	0	0	0.00
64	1	1	1	1	1	1	0	0	0	0	0	0.04
65	0	0	0	0	0	0	1	0	0	0	0	21.66
66	1	0	0	0	0	0	1	0	0	0	0	0.00
67	0	1	0	0	0	0	1	0	0	0	0	0.51
68	1	1	0	0	0	0	1	0	0	0	0	0.00
69	0	0	1	0	0	0	1	0	0	0	0	1.04
70	1	0	1	0	0	0	1	0	0	0	0	0.00
71	0	1	1	0	0	0	1	0	0	0	0	0.02
72	1	1	1	0	0	0	1	0	0	0	0	0.00
73	0	0	0	1	0	0	1	0	0	0	0	0.00
74	1	0	0	1	0	0	1	0	0	0	0	0.00
75	0	1	0	1	0	0	1	0	0	0	0	0.00
76	1	1	0	1	0	0	1	0	0	0	0	0.00
77	0	0	1	1	0	0	1	0	0	0	0	0.00
78	1	0	1	1	0	0	1	0	0	0	0	0.00
79	0	1	1	1	0	0	1	0	0	0	0	0.00
80	1	1	1	1	0	0	1	0	0	0	0	0.01
81	0	0	0	0	1	0	1	0	0	0	0	0.05
82	1	0	0	0	1	0	1	0	0	0	0	0.00
83	0	1	0	0	1	0	1	0	0	0	0	0.00
84	1	1	0	0	1	0	1	0	0	0	0	0.00
85	0	0	1	0	1	0	1	0	0	0	0	0.00
86	1	0	1	0	1	0	1	0	0	0	0	0.00
87	0	1	1	0	1	0	1	0	0	0	0	0.00
88	1	1	1	0	1	0	1	0	0	0	0	0.00
89	0	0	0	1	1	0	1	0	0	0	0	0.00
90	1	0	0	1	1	0	1	0	0	0	0	0.00
91	0	1	0	1	1	0	1	0	0	0	0	0.00
92	1	1	0	1	1	0	1	0	0	0	0	0.00
93	0	0	1	1	1	0	1	0	0	0	0	0.00

94	1	0	1	1	1	0	1	0	0	0	0	0.00
95	0	1	1	1	1	0	1	0	0	0	0	0.00
96	1	1	1	1	1	0	1	0	0	0	0	0.04
97	0	0	0	0	0	1	1	0	0	0	0	0.51
98	1	0	0	0	0	1	1	0	0	0	0	0.00
99	0	1	0	0	0	1	1	0	0	0	0	0.01
100	1	1	0	0	0	1	1	0	0	0	0	0.00
101	0	0	1	0	0	1	1	0	0	0	0	0.03
102	1	0	1	0	0	1	1	0	0	0	0	0.01
103	0	1	1	0	0	1	1	0	0	0	0	0.01
104	1	1	1	0	0	1	1	0	0	0	0	0.13
105	0	0	0	1	0	1	1	0	0	0	0	0.00
106	1	0	0	1	0	1	1	0	0	0	0	0.00
107	0	1	0	1	0	1	1	0	0	0	0	0.00
108	1	1	0	1	0	1	1	0	0	0	0	0.03
109	0	0	1	1	0	1	1	0	3	0	0	0.01
110	1	0	1	1	0	1	1	0	0	0	0	0.10
111	0	1	1	1	0	1	1	0	0	0	0	0.09
112	1	1	1	1	0	1	1	0	0	0	0	1.47
113	0	0	0	0	1	1	1	0	0	0	0	0.00
114	1	0	0	0	1	1	1	0	0	0	0	0.00
115	0	1	0	0	1	1	1	0	0	0	0	0.00
116	1	1	0	0	1	1	1	0	0	0	0	0.01
117	0	0	1	0	1	1	1	0	0	0	0	0.00
118	1	0	1	0	1	1	1	0	0	0	0	0.03
119	0	1	1	0	1	1	1	0	0	0	0	0.02
120	1	1	1	0	1	1	1	0	0	0	0	0.36
121	0	0	0	1	1	1	1	0	1	0	0	0.00
122	1	0	0	1	1	1	1	0	0	0	0	0.01
123	0	1	0	1	1	1	1	0	0	0	0	0.00
124	1	1	0	1	1	1	1	0	0	0	0	0.08
125	0	0	1	1	1	1	1	0	2	0	0	0.02
126	1	0	1	1	1	1	1	0	0	0	0	0.28
127	0	1	1	1	1	1	1	0	0	0	0	0.24
128	1	1	1	1	1	1	1	0	0	0	0	3.99
129	0	0	0	0	0	0	0	1	0	0	0	30.73
130	1	0	0	0	0	0	0	1	0	0	0	0.00
131	0	1	0	0	0	0	0	1	0	0	0	0.72
132	1	1	0	0	0	0	0	1	0	0	0	0.00
133	0	0	1	0	0	0	0	1	0	0	0	1.48
134	1	0	1	0	0	0	0	1	0	0	0	0.00
135	0	1	1	0	0	0	0	1	0	0	0	0.03
136	1	1	1	0	0	0	0	1	0	0	0	0.00
137	0	0	0	1	0	0	0	1	0	0	0	0.00

138	1	0	0	1	0	0	0	1	0	0	0	0.00
139	0	1	0	1	0	0	0	1	0	0	0	0.00
140	1	1	0	1	0	0	0	1	0	0	0	0.00
141	0	0	1	1	0	0	0	1	0	0	0	0.00
142	1	0	1	1	0	0	0	1	0	0	0	0.00
143	0	1	1	1	0	0	0	1	0	0	0	0.00
144	1	1	1	1	0	0	0	1	0	0	0	0.00
145	0	0	0	0	1	0	0	1	0	0	0	0.07
146	1	0	0	0	1	0	0	1	0	0	0	0.00
147	0	1	0	0	1	0	0	1	0	0	0	0.00
148	1	1	0	0	1	0	0	1	0	0	0	0.00
149	0	0	1	0	1	0	0	1	0	0	0	0.00
150	1	0	1	0	1	0	0	1	0	0	0	0.00
151	0	1	1	0	1	0	0	1	0	0	0	0.00
152	1	1	1	0	1	0	0	1	0	0	0	0.00
153	0	0	0	1	1	0	0	1	0	0	0	0.00
154	1	0	0	1	1	0	0	1	0	0	0	0.00
155	0	1	0	1	1	0	0	1	0	0	0	0.00
156	1	1	0	1	1	0	0	1	0	0	0	0.00
157	0	0	1	1	1	0	0	1	0	0	0	0.00
158	1	0	1	1	1	0	0	1	0	0	0	0.00
159	0	1	1	1	1	0	0	1	0	0	0	0.00
160	1	1	1	1	1	0	0	1	0	0	0	0.01
161	0	0	0	0	0	1	0	1	0	0	0	0.72
162	1	0	0	0	0	1	0	1	0	0	0	0.00
163	0	1	0	0	0	1	0	1	10	0	0	0.02
164	1	1	0	0	0	1	0	1	0	0	0	0.00
165	0	0	1	0	0	1	0	1	0	0	0	0.03
166	1	0	1	0	0	1	0	1	0	0	0	0.00
167	0	1	1	0	0	1	0	1	0	0	0	0.00
168	1	1	1	0	0	1	0	1	0	0	0	0.02
169	0	0	0	1	0	1	0	1	0	0	0	0.00
170	1	0	0	1	0	1	0	1	0	0	0	0.00
171	0	1	0	1	0	1	0	1	0	0	0	0.00
172	1	1	0	1	0	1	0	1	0	0	0	0.00
173	0	0	1	1	0	1	0	1	0	0	0	0.00
174	1	0	1	1	0	1	0	1	0	0	0	0.01
175	0	1	1	1	0	1	0	1	0	0	0	0.01
176	1	1	1	1	0	1	0	1	0	0	0	0.20
177	0	0	0	0	1	1	0	1	0	0	0	0.00
178	1	0	0	0	1	1	0	1	0	0	0	0.00
179	0	1	0	0	1	1	0	1	0	0	0	0.00
180	1	1	0	0	1	1	0	1	0	0	0	0.00
181	0	0	1	0	1	1	0	1	0	0	0	0.00

182	1	0	1	0	1	1	0	1	0	0	0	0.00
183	0	1	1	0	1	1	0	1	0	0	0	0.00
184	1	1	1	0	1	1	0	1	0	0	0	0.05
185	0	0	0	1	1	1	0	1	0	0	0	0.00
186	1	0	0	1	1	1	0	1	0	0	0	0.00
187	0	1	0	1	1	1	0	1	0	0	0	0.00
188	1	1	0	1	1	1	0	1	0	0	0	0.01
189	0	0	1	1	1	1	0	1	0	0	0	0.00
190	1	0	1	1	1	1	0	1	0	0	0	0.04
191	0	1	1	1	1	1	0	1	0	0	0	0.03
192	1	1	1	1	1	1	0	1	0	0	0	0.54
193	0	0	0	0	0	0	1	1	6	0	0	1.95
194	1	0	0	0	0	0	1	1	0	0	0	0.00
195	0	1	0	0	0	0	1	1	0	0	0	0.05
196	1	1	0	0	0	0	1	1	0	0	0	0.00
197	0	0	1	0	0	0	1	1	20	0	0	0.09
198	1	0	1	0	0	0	1	1	0	0	0	0.00
199	0	1	1	0	0	0	1	1	0	0	0	0.00
200	1	1	1	0	0	0	1	1	0	0	0	0.02
201	0	0	0	1	0	0	1	1	0	0	0	0.00
202	1	0	0	1	0	0	1	1	0	0	0	0.00
203	0	1	0	1	0	0	1	1	0	0	0	0.00
204	1	1	0	1	0	0	1	1	0	0	0	0.00
205	0	0	1	1	0	0	1	1	0	0	0	0.00
206	1	0	1	1	0	0	1	1	0	0	0	0.01
207	0	1	1	1	0	0	1	1	0	0	0	0.01
208	1	1	1	1	0	0	1	1	0	0	0	0.20
209	0	0	0	0	1	0	1	1	0	0	0	0.00
210	1	0	0	0	1	0	1	1	0	0	0	0.00
211	0	1	0	0	1	0	1	1	0	0	0	0.00
212	1	1	0	0	1	0	1	1	0	0	0	0.00
213	0	0	1	0	1	0	1	1	0	0	0	0.00
214	1	0	1	0	1	0	1	1	0	0	0	0.00
215	0	1	1	0	1	0	1	1	0	0	0	0.00
216	1	1	1	0	1	0	1	1	0	0	0	0.05
217	0	0	0	1	1	0	1	1	0	0	0	0.00
218	1	0	0	1	1	0	1	1	0	0	0	0.00
219	0	1	0	1	1	0	1	1	0	0	0	0.00
220	1	1	0	1	1	0	1	1	0	0	0	0.01
221	0	0	1	1	1	0	1	1	0	0	0	0.00
222	1	0	1	1	1	0	1	1	0	0	0	0.04
223	0	1	1	1	1	0	1	1	0	0	0	0.03
224	1	1	1	1	1	0	1	1	0	0	0	0.55
225	0	0	0	0	0	1	1	1	0	0	0	0.05

226	1	0	0	0	0	1	1	1	0	0	0	0.00
227	0	1	0	0	0	1	1	1	0	0	0	0.00
228	1	1	0	0	0	1	1	1	0	0	0	0.04
229	0	0	1	0	0	1	1	1	0	0	0	0.01
230	1	0	1	0	0	1	1	1	0	0	0	0.13
231	0	1	1	0	0	1	1	1	0	0	0	0.11
232	1	1	1	0	0	1	1	1	8	0	0	1.93
233	0	0	0	1	0	1	1	1	0	0	0	0.00
234	1	0	0	1	0	1	1	1	1	0	0	0.03
235	0	1	0	1	0	1	1	1	0	0	0	0.02
236	1	1	0	1	0	1	1	1	0	0	0	0.40
237	0	0	1	1	0	1	1	1	0	0	0	0.09
238	1	0	1	1	0	1	1	1	0	0	0	1.47
239	0	1	1	1	0	1	1	1	0	0	0	1.25
240	1	1	1	1	0	1	1	1	17	0	0	21.15
241	0	0	0	0	1	1	1	1	0	0	0	0.00
242	1	0	0	0	1	1	1	1	0	0	0	0.01
243	0	1	0	0	1	1	1	1	0	0	0	0.01
244	1	1	0	0	1	1	1	1	0	0	0	0.10
245	0	0	1	0	1	1	1	1	0	0	0	0.02
246	1	0	1	0	1	1	1	1	0	0	0	0.36
247	0	1	1	0	1	1	1	1	0	0	0	0.31
248	1	1	1	0	1	1	1	1	0	0	0	5.25
249	0	0	0	1	1	1	1	1	0	0	0	0.00
250	1	0	0	1	1	1	1	1	0	0	0	0.08
251	0	1	0	1	1	1	1	1	0	0	0	0.06
252	1	1	0	1	1	1	1	1	0	0	0	1.09
253	0	0	1	1	1	1	1	1	0	0	0	0.24
254	1	0	1	1	1	1	1	1	0	0	0	4.00
255	0	1	1	1	1	1	1	1	0	0	0	3.40
256	1	1	1	1	1	1	1	1	75	0	0	57.57

	ExpRefInd	ExpRefInf
1	0	0
2	0	0
3	0	0
4	0	0
5	0	0
6	0	0
7	0	0
8	0	0
9	0	0
10	0	0
11	0	0
12	0	0

13	0	0
14	0	0
15	0	0
16	0	0
17	0	0
18	0	0
19	0	0
20	0	0
21	0	0
22	0	0
23	0	0
24	0	0
25	0	0
26	0	0
27	0	0
28	0	0
29	0	0
30	0	0
31	0	0
32	0	0
33	0	0
34	0	0
35	0	0
36	0	0
37	0	0
38	0	0
39	0	0
40	0	0
41	0	0
42	0	0
43	0	0
44	0	0
45	0	0
46	0	0
47	0	0
48	0	0
49	0	0
50	0	0
51	0	0
52	0	0
53	0	0
54	0	0
55	0	0
56	0	0

57	0	0
58	0	0
59	0	0
60	0	0
61	0	0
62	0	0
63	0	0
64	0	0
65	0	0
66	0	0
67	0	0
68	0	0
69	0	0
70	0	0
71	0	0
72	0	0
73	0	0
74	0	0
75	0	0
76	0	0
77	0	0
78	0	0
79	0	0
80	0	0
81	0	0
82	0	0
83	0	0
84	0	0
85	0	0
86	0	0
87	0	0
88	0	0
89	0	0
90	0	0
91	0	0
92	0	0
93	0	0
94	0	0
95	0	0
96	0	0
97	0	0
98	0	0
99	0	0
100	0	0

101	0	0
102	0	0
103	0	0
104	0	0
105	0	0
106	0	0
107	0	0
108	0	0
109	0	0
110	0	0
111	0	0
112	0	0
113	0	0
114	0	0
115	0	0
116	0	0
117	0	0
118	0	0
119	0	0
120	0	0
121	0	0
122	0	0
123	0	0
124	0	0
125	0	0
126	0	0
127	0	0
128	0	0
129	0	0
130	0	0
131	0	0
132	0	0
133	0	0
134	0	0
135	0	0
136	0	0
137	0	0
138	0	0
139	0	0
140	0	0
141	0	0
142	0	0
143	0	0
144	0	0

145	0	0
146	0	0
147	0	0
148	0	0
149	0	0
150	0	0
151	0	0
152	0	0
153	0	0
154	0	0
155	0	0
156	0	0
157	0	0
158	0	0
159	0	0
160	0	0
161	0	0
162	0	0
163	0	0
164	0	0
165	0	0
166	0	0
167	0	0
168	0	0
169	0	0
170	0	0
171	0	0
172	0	0
173	0	0
174	0	0
175	0	0
176	0	0
177	0	0
178	0	0
179	0	0
180	0	0
181	0	0
182	0	0
183	0	0
184	0	0
185	0	0
186	0	0
187	0	0
188	0	0

189	0	0
190	0	0
191	0	0
192	0	0
193	0	0
194	0	0
195	0	0
196	0	0
197	0	0
198	0	0
199	0	0
200	0	0
201	0	0
202	0	0
203	0	0
204	0	0
205	0	0
206	0	0
207	0	0
208	0	0
209	0	0
210	0	0
211	0	0
212	0	0
213	0	0
214	0	0
215	0	0
216	0	0
217	0	0
218	0	0
219	0	0
220	0	0
221	0	0
222	0	0
223	0	0
224	0	0
225	0	0
226	0	0
227	0	0
228	0	0
229	0	0
230	0	0
231	0	0
232	0	0

233	0	0
234	0	0
235	0	0
236	0	0
237	0	0
238	0	0
239	0	0
240	0	0
241	0	0
242	0	0
243	0	0
244	0	0
245	0	0
246	0	0
247	0	0
248	0	0
249	0	0
250	0	0
251	0	0
252	0	0
253	0	0
254	0	0
255	0	0
256	0	0

```
$Test
 Max LogLikelihood: Achievable  Obtained Deviance d.f. p value
                  -549.7375 -867.0493 634.6235  238       0

$Commentary
[1] "The model does not fit: Assumptions may be not justified"


Residual  correlations between test
$ResCor
          Corr1-2    Corr1-3     Corr1-4    Corr1-5      Corr1-6
pop 1 : 0.06575963 0.01004889 -0.005808462 0.01873478 -0.0004674799
            Corr1-7    Corr1-8    Corr2-3      Corr2-4    Corr2-5   Corr2-6
pop 1 : -0.0004883415 0.06089272 0.01525626 -0.006745719 0.02715726 0.1078324
           Corr2-7   Corr2-8      Corr3-4     Corr3-5      Corr3-6   Corr3-7
pop 1 : -0.007035815 0.1546329 -0.001764633 0.005236108 -0.005072248 0.1905448
         Corr3-8    Corr4-5    Corr4-6    Corr4-7     Corr4-8      Corr5-6
pop 1 : 0.1928594 0.07539012 0.01080085 0.01028548 0.004556416 -0.003538629
           Corr5-7    Corr5-8     Corr6-7   Corr6-8   Corr7-8
```

pop 1 : -0.003862773 0.01239704 0.004099339 0.1041175 0.2442151

$Commentary
[1] "The residuals should be randomly distributed around 0"

```
 BOOTSTRAP CONFIDENCE INTERVALS : 5000  samples
       pre1 Sp1    Sp2    Sp3 Sp4   Sp5    Sp6    Sp7    Sp8    Se1    Se2
CIinf 0.1644   1 0.9621 0.9339   1 0.993 0.9621 0.9173 0.8912 0.8968 0.8860
CIsup 0.2339   1 0.9907 0.9729   1 1.000 0.9907 0.9619 0.9427 0.9829 0.9794
       Se3    Se4    Se5   Se6   Se7    Se8
CIinf 0.95 0.8600 0.6465 0.967 0.967 0.8854
CIsup 1.00 0.9639 0.8132 1.000 1.000 0.9794
```

your data are stored in "new_8_single"
Note that you'll have to Save workspace image before leaving R if you want to use it in a new R session
> save.image("D:\\Dropbox\\Publications\\ABPA LCA salami\\n11n1n2n5n10n12n13_single"
